# Collaborative Hierarchical Multi-Agent Learning for RIS-Assisted Multi-Cell Integrated Sensing and Communication

**DOI:** 10.3390/s26216887

**Published:** 2026-10-30

**Authors:** Ammar Summaq, Mukkara Prasanna Kumar, Karthikeyan Elumalai, Poongundran Selvaprabhu, Sunil Chinnadurai

**Affiliations:** 1 Department of Electronics and Communication Engineering, School of Engineering and Science, SRM University–AP, Amaravathi 522240, Andhra Pradesh, India; ammar_summaq@srmap.edu.in (A.S.); mukkaraprasanna_kumar@srmap.edu.in (M.P.K.); 2 Department of Electronics and Communication Engineering, IIT Bhilai, Bhilai 491002, Chhattisgarh, India; karthikeyan@iitbhilai.ac.in; 3 Department of Communication Engineering, School of Electronics and Engineering, Vellore Institute of Technology, Vellore 632014, Tamil Nadu, India; poongundran.selvaprabhu@vit.ac.in

**Keywords:** integrated sensing and communications (ISAC), RIS, multi-cell coordination, hierarchical DRL, centralized training and decentralized execution (CTDE), hierarchical multi-agent reinforcement learning (MARL), user association, 6G

## Abstract

The convergence of integrated sensing and communication (ISAC) and reconfigurable intelligent surfaces (RISs) offers new opportunities for building adaptive and energy-efficient 6G wireless networks. By jointly exploiting sensing information and programmable wireless propagation, these technologies can support intelligent network operation in highly dynamic environments. However, realizing these benefits in multi-user and multi-cell networks requires jointly controlling several strongly interconnected variables, including transmit beamforming, RIS configurations, power allocation, and user association. This problem becomes increasingly difficult in the presence of user mobility, imperfect channel state information (CSI), and strong interference among neighboring cells. Existing iterative optimization algorithms can become computationally demanding for large-scale networks, whereas conventional flat multi-agent reinforcement learning (MARL) approaches often struggle with the rapidly growing action space and limited inter-agent coordination. To address these limitations, this study develops an HDRL-ISAC framework based on hierarchical multi-agent learning and centralized training with decentralized execution (CTDE). The proposed framework assigns different decision-making roles across the hierarchy, allowing high-level agents to coordinate network-wide cellular decisions while lower-level agents perform user-specific resource optimization. This hierarchical decomposition improves the learning efficiency and enables scalable coordination in multi-cell environments. Furthermore, sensing information is incorporated into the learning process through EKF-based mobility prediction, enabling agents to anticipate user movement and adapt their resource allocation decisions accordingly. Lightweight information exchange among neighboring cells is also introduced to facilitate interference-aware coordination under imperfect CSI. Simulation results demonstrate that the proposed HDRL-ISAC approach improves energy efficiency by up to 35%, reduces inter-cell interference leakage by up to 6 dB, and decreases ping-pong handovers by up to 40% compared to benchmark schemes. Crucially, the proposed architecture achieves approximately 1.8× faster training convergence than conventional flat MARL approaches while avoiding the prohibitive online computational delays associated with conventional iterative optimization methods. These findings indicate that hierarchical and sensing-aware multi-agent learning provides a promising approach for scalable resource management in future RIS-assisted multi-cell ISAC networks.

## 1. Introduction

Sixth-generation (6G) wireless networks are envisioned to transcend the capacity ceiling of 5G by simultaneously delivering sub-millisecond latency, terabit-per-second peak throughput, carrier-grade reliability ($10^{-5}$ outage), and massive spatial densification while operating under energy budgets that are an order of magnitude tighter than their predecessors [1,2,3]. Beyond raw throughput, 6G must natively support a spectrum of heterogeneous services, ranging from enhanced mobile broadband (eMBB) to ultra-reliable low-latency communications (URLLC) and massive machine-type communications (mMTC), each imposing distinct and often conflicting resource allocation demands [4,5]. Meeting these requirements in dense urban deployments, where hundreds of base stations (BSs), thousands of user equipment (UEs), and an ever-growing ecosystem of sensing nodes coexist, demands a fundamental re-architecture of how spectrum, power, and space are jointly managed [1,6].

Integrated Sensing and Communication (ISAC)has emerged as one of the most impactful enablers of this re-architecture by unifying radar-sensing functionality with data transmission in a shared spectrum and hardware platform [4,7]. ISAC exploits echoes from communication waveforms to simultaneously estimate user kinematics (angle-of-arrival, time-of-arrival, Doppler), enabling environment-aware beam management, proactive handover prediction, and interference coordination without dedicated sensing overhead [2,7,8]. In vehicular and high-mobility scenarios, ISAC-derived state estimates fed into tracking filters such as the extended Kalman filter (EKF) have been shown to substantially reduce ping-pong handovers and improve edge-user throughput, thereby validating the cross-layer benefit of tightly co-designed sensing and communication control [5,7]. However, the sensing–communication trade-off becomes sharply non-convex in multi-cell, multi-user regimes, where inter-cell interference couples beamforming vectors across BSs and a purely per-cell signal-to-interference-plus-noise ratio (SINR) maximization approach fails to exploit cooperative gains [1,2].

Reconfigurable Intelligent Surfaces (RISs) provide a complementary and cost-efficient lever for programmable propagation: by electronically tuning the phase (and optionally amplitude) of a large passive reflective array, an RIS can constructively steer desired signals toward intended receivers, null interference toward cell-edge neighbors, and even extend the bistatic sensing aperture at negligible additional power cost [2,6,9]. Recent studies on RIS-assisted cooperative multi-cell ISAC have characterized multi-user and multi-target regimes, revealing that ignoring inter-cell reflections can degrade both sensing accuracy and communication SINR by several decibels, and that symbol-level precoding in coordinated multi-point (CoMP) architectures can better exploit constructive interference for joint performance albeit at higher coordination overhead [1,2]. Extensions to near-field, circular, and multi-RIS configurations further demonstrate gains in target indication, three-dimensional localization, and synchronization while simultaneously highlighting mutual coupling effects that complicate echo-path interpretation and Fisher information computation [6]. Hardware-aware constraints, including *B*-bit discrete phase alphabets and non-uniform quantization grids, impose combinatorial feasibility requirements on RIS optimization, motivating vector-quantized phase designs and learning-compatible projection layers that preserve interference suppression even at coarse resolution [9].

Learning-based optimization has become indispensable for tackling the resulting joint non-convex, combinatorial, and partially observable design problem at scale. Deep reinforcement learning (DRL) has been successfully applied to RIS-assisted ISAC for beamforming and phase control, age-of-information (AoI)-aware resource allocation in RIS-aided vehicular networks, and full-duplex quantized-RIS systems, collectively demonstrating that policy-gradient methods can match or exceed alternating-optimization baselines without requiring explicit closed-form channel models [4,5,7]. Despite these advances, single-agent DRL methods do not scale gracefully to multi-cell topologies because the joint state-action space grows exponentially with the number of BSs and RIS elements, and because the environment becomes non-stationary from the perspective of any individual agent as co-located agents simultaneously update their policies [1,3].

Multi-Agent Reinforcement Learning (MARL) directly addresses this scalability bottleneck by distributing decision-making across per-cell or per-user agents [3]. Among MARL paradigms, centralized training and decentralized execution (CTDE) has established itself as the dominant architecture for cooperative wireless control: a centralized critic conditions on joint observations during offline training to stabilize value estimation, whereas decentralized actors execute using only local observations and limited inter-cell messages at runtime [3,7]. Hierarchical extensions of MARL further decompose temporally and spatially coupled decisions into a master–slave structure, where coarse cell-level coordination (RIS codebook selection, inter-cell interference budgets) is handled at a slower timescale by master agents, and fine user-level actuation (per-user power, beam index, association) is handled at a faster timescale by slave agents [2,3]. Empirical results from adjacent wireless and cyber-physical domains confirm that hierarchical CTDE achieves faster convergence, better interference fairness, and lower ping-pong rates than flat MARL with the same action budgets [3,7].

Research gap and motivation. Despite this encouraging body of literature, several critical gaps remain unaddressed. First, the existing RIS-assisted ISAC studies predominantly target single-cell or two-cell scenarios with fixed user association [4,7]; scaling to C>3 cells with dynamic handover-aware association and heterogeneous mobility has not been systematically treated, leaving the inter-cell interference and ping-pong suppression problem open. Second, multi-cell ISAC precoding works such as [1,2] adopt centralized optimization frameworks that assume perfect or near-perfect channel state information (CSI) and do not incorporate reinforcement learning or EKF-based tracking, making them fragile under imperfect CSI and non-stationary traffic. Third, the existing MARL frameworks for wireless networks [3] rarely integrate sensing-derived state information into an agent’s observation space, discarding the kinematic awareness that ISAC can uniquely provide for proactive beam management and handover control. Fourth, hardware constraints such as discrete RIS phase alphabets and quantized BS beams are typically relaxed or handled in post-processing, whereas practical deployment requires embedding feasibility projections and codebook-based actions natively within the learning loop. Fifth, joint energy efficiency, spectral efficiency, sensing accuracy, and handover stability have not been simultaneously co-optimized within a single hierarchical CTDE framework for multi-cell RIS-ISAC networks, and existing work optimizes at most two of these objectives and often ignores inter-cell coordination overhead budgets.

### 1.1. Related Work

Multi-cell ISAC precoding has advanced from coordinated beamforming (CBF) to CoMP, balancing communication SINR and sensing accuracy via Cramér–Rao bound (CRB)-based formulations and robust optimization under CSI uncertainty. Prior studies show that ignoring inter-cell reflections degrades both sensing and communication, and that symbol-level precoding in CoMP can better exploit constructive interference for joint performance, albeit with higher coordination overhead [1].

RIS-assisted cooperative multi-cell ISAC systems leverage programmable environments to enhance joint transmission and bistatic/monostatic sensing, with recent work characterizing multi-user and multi-target regimes and revealing coordination–overhead trade-offs [2]. Extensions toward near-field and multi-RIS configurations demonstrate gains in target indication, 3D localization, and synchronization, highlighting the importance of array geometry and calibration for practical deployment [6,10]. Furthermore, coupling effects at RIS panels motivate localization methods that account for mutual coupling when interpreting echo-path structure and Fisher information [11].

Learning-based ISAC with RIS has emerged as a way to tame nonconvex joint design at scale. Deep RL has been applied to RIS-assisted ISAC for beamforming/phase control, and AoI-aware resource allocation in RIS-aided vehicular networks underscores the benefit of policy learning under dynamic traffic [4,5]. Recent DRL formulations also explored joint beam–phase optimization using SAC-like updates, reporting improved rate–sensing trade-offs with reduced model dependence compared to alternating optimization baselines [12].

On the control architecture side, hierarchical and MARL provide a scalable path for decentralized wireless control. CTDE improves sample efficiency by conditioning centralized critics on joint observations while actors remain local [3]. Empirical HMARL results in adjacent wireless and cyber-physical domains (e.g., coordinated spatial reuse and cyber defense) show faster convergence and better interference/fairness management, motivating hierarchical designs for multi-cell coordination [13,14].

Hardware-aware constraints at RIS further motivate codebook-based actions: vector-quantized phase design and non-uniform phase quantization improve discrete-phase implementations and guide feasibility projections in learning loops [9,15]. Complementary ISAC precoding advances for multiple-input multiple-output orthogonal frequency-division multiplexing (MIMO-OFDM) and symbol-level ISAC underscore the importance of waveform/precoder co-design under multi-cell interference but typically assume centralized optimization without hierarchical CTDE and handover-aware association [16,17].

In contrast to the above, this work develops a CTDE-hierarchical deep reinforcement learning for ISAC (HDRL-ISAC) tailored for multi-user, multi-cell RIS-assisted networks that combines master–slave agents, inter-cell message passing, and ISAC/EKF-informed state representations to jointly coordinate beams, RIS phases, association, and power under imperfect CSI and mobility, addressing co-channel interference and handover stability at scale [1,2,3,4].

### 1.2. Contributions

To address the aforementioned research gaps, this paper makes the following key contributions:

- We developed a hierarchical CTDE-based multi-agent reinforcement learning framework, called HDRL-ISAC, for collaborative RIS-assisted multi-cell ISAC networks. Unlike conventional flat MARL architectures, the proposed master–slave framework decomposes global cell coordination and local user resource optimization, enabling the joint optimization of collaborative RIS configuration, coordinated beamforming, user association, and transmit-power allocation under imperfect CSI and dynamic user mobility while substantially improving scalability and learning stability [1,2,3,7].
- We proposed an ISAC-aware hierarchical decision architecture that tightly integrates communication, sensing, and mobility prediction. The framework combines EKF-based user-state estimation, ISAC-derived sensing information, and lightweight neighboring-cell message exchange to provide mobility-aware resource allocation, proactive handover management, and cooperative inter-cell interference mitigation with a low signaling overhead [18].
- We designed a practical multi-objective reinforcement learning formulation for multi-cell ISAC optimization. A normalized entropy-regularized reward jointly optimizes the energy efficiency, spectral efficiency, inter-cell interference suppression, localization accuracy, and handover stability while incorporating practical RIS hardware constraints, finite-resolution phase codebooks, and quality of service (QoS) requirements into the learning process.
- We validated the proposed HDRL-ISAC framework through comprehensive simulations under realistic 3GPP-inspired multi-cell scenarios. The proposed method consistently outperforms conventional optimization methods and flat MARL baselines, achieving up to 35% higher energy efficiency, reducing inter-cell interference leakage by up to 6 dB, decreasing ping-pong handovers by 40%, and converging approximately 1.8× faster under imperfect CSI and user mobility [1,2,3,5,6,7,9].

### 1.3. Organization of the Paper

The remainder of this paper is organized as follows. Section 2 presents the system model, including the RIS codebook, channel and signal models, ISAC-assisted state estimation, mobility model, and the problem formulation. Section 3 introduces the proposed HDRL-ISAC framework, including the hierarchical CTDE architecture, state and action design, constraint-aware action enforcement, and reward formulation. Section 4 provides a theoretical analysis of the proposed framework, including Bellman-operator convergence, policy improvement, and hierarchical learning stability. Section 5 presents the proposed HDRL-ISAC algorithm together with the inter-cell message exchange and aggregation procedures. Section 6 presents an analysis of the computational complexity and communication overhead. Section 7 describes the simulation setup, performance metrics, and the benchmark schemes. Section 8 presents and discusses the simulation results under different network conditions. Finally, Section 9 concludes the study and outlines future research directions.

## 2. System Model

Consider a multi-cell RIS-assisted ISAC network with *C* cells. Each cell, i∈{1,…,C}, contains an *M*-antenna base station (BS), an *N*-element reconfigurable intelligent surface (RIS), and a set of single-antenna users $U_{i}$. The active-user set is

(1) $$U=\overset{C}{\underset{i=1}{⋃}}U_{i},\;U=\left|U\right|.$$

The BSs simultaneously support downlink communication and sensing while users move across neighboring cells, causing time-varying channels, association, interference, and sensing observations. The proposed framework jointly optimizes communication, sensing, RIS configuration, power allocation, and mobility-aware coordination subject to the physical and QoS constraints defined below.

**Figure 1:**
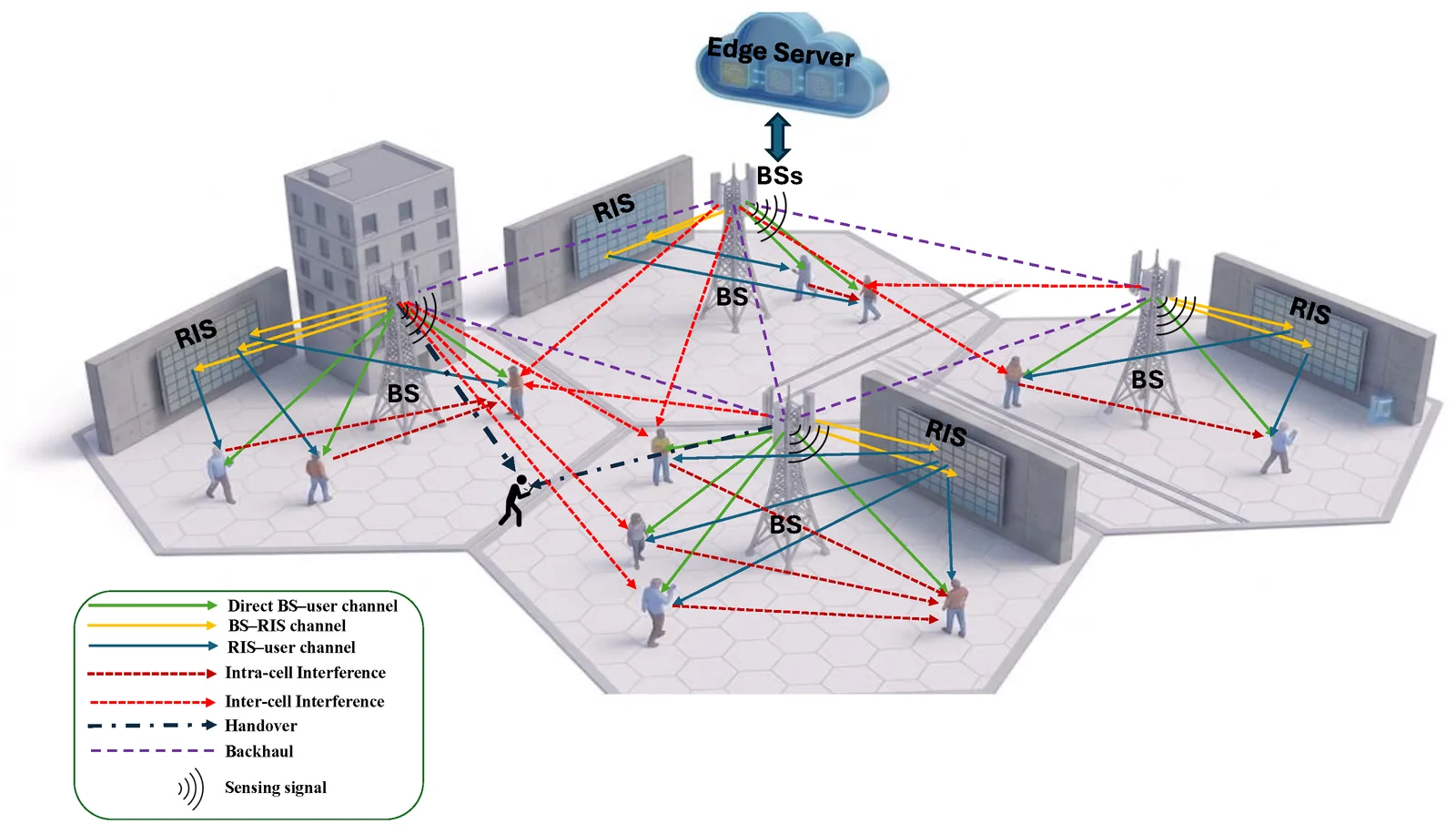
Multi-cell RIS-assisted ISAC system architecture. Each cell contains a multi-antenna base station (BS), a reconfigurable intelligent surface (RIS), and multiple mobile users. The BS performs simultaneous communication and sensing, while neighboring BSs exchange compact coordination information through the Xn/backhaul interface. The system includes direct BS–UE, BS–RIS, and RIS–UE links, intra-cell and inter-cell interference, user mobility and handovers, and cooperative inter-cell coordination.

Figure 1 illustrates the architecture considered. During centralized training, global information is exploited according to the CTDE, whereas decentralized execution uses local observations and the latest valid neighboring-cell summaries.

### 2.1. Finite-Resolution RIS Codebook

The RIS associated with BS *i* comprises *N* passive reflecting elements with unit-amplitude and finite-resolution phase shifts.

(2) $$\Theta_{i,t}=\mathrm{diag}\left(e^{j\theta_{i,1,t}},\ldots ,e^{j\theta_{i,N,t}}\right).$$

where the feasible phase set is

(3) $$Q_{B_{\mathrm{RIS}}}=\left\{\frac{2\pi m}{2^{B_{\mathrm{RIS}}}}\;|\;m=0,\ldots ,2^{B_{\mathrm{RIS}}}-1\right\}.$$

Thus, each element supports $2^{B_{\mathrm{RIS}}}$ phase levels; in the numerical evaluation, $B_{\mathrm{RIS}}=3$ provides eight levels.

Element-wise optimization would yield

(4) $$2^{B_{\mathrm{RIS}}N}$$

possible phase configurations. To reduce this control space, a finite set of precomputed RIS configurations is used:

(5) $$C_{\Theta }=\left\{\Theta^{\left(1\right)},\ldots ,\Theta^{\left(B_{\Theta }\right)}\right\},\;B_{\Theta }=\left|C_{\Theta }\right|.$$

where each codeword satisfies $\theta_{n}^{\left(b\right)}\in Q_{B_{\mathrm{RIS}}}$. The codebook is constructed offline and stored at the BS, whereas the policy directly selects its codeword index $c_{i,t}^{\Theta }$ during execution:

(6) $$\Theta_{i,t}=\Theta^{\left(c_{i,t}^{\Theta }\right)}.$$

Hence, the RIS control is formulated as a discrete codeword selection rather than an element-wise continuous phase optimization.

### 2.2. Channel Model

For cell *i*, let $G_{i,t}\in C^{N\times M}$ denote the BS–RIS channel, $h_{i,k,t}\in C^{N\times 1}$ the RIS–UE channel, and $d_{i,k,t}\in C^{M\times 1}$ the direct BS–UE channel. Under the adopted column-vector convention, the equivalent BS-to-user channel is

(7) $$h_{i,k,t}^{\mathrm{eff}}=d_{i,k,t}+G_{i,t}^{H}\Theta_{i,t}^{H}h_{i,k,t}.$$

which incorporates both direct and RIS-assisted propagation.

For user (i,k) served by BS *i*, the effective channel from an interfering BS j≠i is

(8) $$h_{j\to i,k,t}^{\mathrm{eff}}=d_{j\to i,k,t}+G_{j,t}^{H}\Theta_{j,t}^{H}h_{j\to i,k,t}.$$

where $d_{j\to i,k,t}\in C^{M\times 1}$ and $h_{j\to i,k,t}\in C^{N\times 1}$ denote the direct BS–UE and RIS–UE channels associated with BS *j*. Thus, (8) captures both the direct and RIS-reflected inter-cell interference.

### 2.3. Rician Propagation Model

All the wireless links follow distance-dependent Rician fading. The Rician factor is denoted by $K_{R}$, where $K_{u}$ denotes the number of active users per cell. The generic channel vector is modeled as follows:

(9) $$h\left(d\right)=\sqrt{\beta (d)}\left(\sqrt{\frac{K_{R}}{K_{R}+1}}h^{\mathrm{LoS}}+\sqrt{\frac{1}{K_{R}+1}}h^{\mathrm{NLoS}}\right).$$

where β(d) is the large-scale path-loss coefficient, and $h^{\mathrm{LoS}}$ and $h^{\mathrm{NLoS}}$ denote the LoS and NLoS components, respectively. The path-loss model is

(10) $$\beta \left(d\right)=C_{0}d^{-\alpha_{\mathrm{PL}}},\;h^{\mathrm{NLoS}}∼\mathrm{CN}\left(0,I\right).$$

where $C_{0}$ is the reference channel-power coefficient, and $\alpha_{\mathrm{PL}}$ is the path-loss exponent.

For a ULA, the normalized LoS steering vector is

(11) $$a_{M}\left(\phi \right)=\frac{1}{\sqrt{M}}{\left[1,e^{j\pi \sin\phi },\ldots ,e^{j\pi (M-1)\sin\phi }\right]}^{T}.$$

where ϕ is the corresponding departure or arrival angle. The simulation values of $K_{R}$ are specified in Table 1.

### 2.4. Received Signal Model

Let $p_{i,k,t}$ denote the transmit power allocated to user *k* by BS *i*, and let $w_{i,k,t}\in C^{M\times 1}$ denote the corresponding normalized beamforming vector. The transmitted signal from BS *i* is

(12) $$x_{i,t}=\sum_{k\in U_{i}\left(t\right)}\sqrt{p_{i,k,t}}\;w_{i,k,t}s_{i,k,t}.$$

where $s_{i,k,t}$ are the normalized information symbols satisfying $E[|s_{i,k,t}{|}^{2}]=1$.

For user (i,k), the received baseband signal is

(13) $$\begin{array}{cc} y_{i,k,t} & =\underset{\mathrm{Desired}\;\mathrm{signal}}{\underset{︸}{\sqrt{p_{i,k,t}}\;h_{i,k,t}^{\mathrm{eff},H}w_{i,k,t}s_{i,k,t}}}\\ \; & \;+\underset{\text{Intra-cell}\;\mathrm{interference}}{\underset{︸}{\sum_{\begin{array}{c} \ell \in U_{i}\left(t\right)\\ \ell \neq k \end{array}}\sqrt{p_{i,\ell ,t}}\;h_{i,k,t}^{\mathrm{eff},H}w_{i,\ell ,t}s_{i,\ell ,t}}}\\ \; & \;+\underset{\text{Inter-cell}\;\mathrm{interference}}{\underset{︸}{\sum_{j\neq i}\sum_{m\in U_{j}\left(t\right)}\sqrt{p_{j,m,t}}\;h_{j\to i,k,t}^{\mathrm{eff},H}w_{j,m,t}s_{j,m,t}}}+n_{i,k,t}. \end{array}$$

The receiver noise is modeled as circularly symmetric complex Gaussian noise,

(14) $$n_{i,k,t}∼\mathrm{CN}\left(0,N_{0}W\right).$$

where $N_{0}$ is the noise power spectral density, and *W* is the system bandwidth.

### 2.5. SINR and Achievable Rate

The instantaneous SINR of user (i,k) is

(15) $$\gamma_{i,k,t}=\frac{a_{i,k,t}p_{i,k,t}{\left|h_{i,k,t}^{\mathrm{eff},H}w_{i,k,t}\right|}^{2}}{I_{i,k,t}^{\mathrm{intra}}+I_{i,k,t}^{\mathrm{inter}}+N_{0}W}.$$

where the intra-cell interference is

(16) $$I_{i,k,t}^{\mathrm{intra}}=\sum_{\begin{array}{c} \ell \in U_{i}\left(t\right)\\ \ell \neq k \end{array}}a_{i,\ell ,t}p_{i,\ell ,t}{\left|h_{i,k,t}^{\mathrm{eff},H}w_{i,\ell ,t}\right|}^{2}.$$

and the inter-cell interference is

(17) $$I_{i,k,t}^{\mathrm{inter}}=\sum_{j\neq i}\sum_{m\in U_{j}\left(t\right)}a_{j,m,t}p_{j,m,t}{\left|h_{j\to i,k,t}^{\mathrm{eff},H}w_{j,m,t}\right|}^{2}.$$

The achievable downlink rate is then

(18) $$R_{i,k,t}=W\log_{2}\left(1+\gamma_{i,k,t}\right).$$

The association indicators in (15)–(17) ensure that only active serving links contribute to the desired signal and intra-/inter-cell transmission terms.

### 2.6. Association and Resource Constraints

Let $a_{i,k,t}\in \left\{0,1\right\}$ denote the association indicator, where $a_{i,k,t}=1$ indicates that user *k* is served by BS *i*. Each active user is associated with exactly one BS:

(19) $$\sum_{i=1}^{C}a_{i,k,t}=1,\;\forall k\in U,\;\forall t.$$

The transmit power satisfies

(20) $$P_{i}^{\min}\leq \sum_{k\in U_{i}\left(t\right)}a_{i,k,t}p_{i,k,t}\leq P_{i}^{\max},\;p_{i,k,t}\geq 0,\;\forall i,t.$$

while the communication QoS and inter-cell interference constraints are

(21) $$R_{i,k,t}\geq R_{k}^{\min},\;\forall k,t\;\mathrm{with}\;a_{i,k,t}=1.$$

(22) $$I_{i}^{\mathrm{inter}}\left(t\right)\leq \Gamma_{i},\;\forall i,t.$$

where $\Gamma_{i}$ denotes the prescribed interference threshold. These constraints define the feasible-action region enforced by the deterministic feasibility layer described in Section 3.3.

### 2.7. Energy Efficiency

The user-level energy efficiency is defined as

(23) $$EE_{i,k,t}=\frac{a_{i,k,t}R_{i,k,t}}{a_{i,k,t}p_{i,k,t}+P_{i,k,t}^{c}}.$$

where $P_{i,k,t}^{c}$ are the effective circuit power contributions of user *k*. When BS circuit power $P_{i}^{c}$ is equally distributed among its associated users,

(24) $$P_{i,k,t}^{c}=\frac{P_{i}^{c}}{|U_{i}\left(t\right)|},\;k\in U_{i}\left(t\right).$$

The corresponding network-level energy efficiency is

(25) $$EE_{\mathrm{net}}\left(t\right)=\frac{\sum_{i=1}^{C}\sum_{k\in U_{i}\left(t\right)}a_{i,k,t}R_{i,k,t}}{\sum_{i=1}^{C}\left(\sum_{k\in U_{i}\left(t\right)}a_{i,k,t}p_{i,k,t}+P_{i}^{c}\right)}.$$

### 2.8. ISAC-Assisted State Estimation

To support mobility-aware resource optimization, each BS periodically transmits an ISAC probing waveform while serving the communication users [19,20]. The received sensing signal is modeled as follows:

(26) $$y_{i}^{s}\left(t\right)=h_{i,\mathrm{tgt}}^{H}\left(t\right)\Theta_{i,t}x_{0}\left(t-\tau_{i,t}\right)+n_{\mathrm{rx},i}\left(t\right).$$

where $x_{0}\left(t\right)$ is the sensing waveform, $h_{i,\mathrm{tgt}}\left(t\right)$ is the effective BS–RIS–target channel, $\Theta_{i,t}$ is the RIS reflection matrix, and $\tau_{i,t}$ is the round-trip delay, with

(27) $$n_{\mathrm{rx},i}\left(t\right)∼\mathrm{CN}\left(0,\sigma_{\mathrm{rx}}^{2}\right).$$

From the sensing observations, the AoA, ToA, and Doppler measurements were extracted and fused using an EKF. The two-dimensional mobility state of user (i,k) is

(28) $$x_{i,k,t}={\left[\begin{array}{cccc} x_{i,k,t} & y_{i,k,t} & v_{x,i,k,t} & v_{y,i,k,t} \end{array}\right]}^{T}.$$

with position $\left(x_{i,k,t},y_{i,k,t}\right)$ and velocity $\left(v_{x,i,k,t},v_{y,i,k,t}\right)$. The constant-velocity prediction model is

(29) $$x_{i,k,t}=Fx_{i,k,t-1}+w_{i,k,t},\;w_{i,k,t}∼N\left(0,Q\right).$$

where

(30) $$F=\left[\begin{array}{cccc} 1 & 0 & \Delta t & 0\\ 0 & 1 & 0 & \Delta t\\ 0 & 0 & 1 & 0\\ 0 & 0 & 0 & 1 \end{array}\right].$$

where Δt denotes the EKF update interval. This constant-velocity model provides the local predictor, whereas the Gauss–Markov model in Section 2.9 generates temporally correlated trajectories for simulation.

The nonlinear ISAC measurement model is [21,22]

(31) $$z_{i,k,t}=h_{i,k}\left(x_{i,k,t}\right)+\nu_{i,k,t},\;z_{i,k,t}=\left[\begin{array}{c} {\hat{\alpha }}_{i,k,t}\\ {\hat{\tau }}_{i,k,t}\\ {\hat{f}}_{D,i,k,t} \end{array}\right].$$

where the measurement vector contains the AoA, ToA, and Doppler frequency, $h_{i,k}\left(\cdot \right)$, maps the mobility state to these measurements, and $\nu_{i,k,t}$ denotes the measurement noise. The sensing-resource-dependent covariance is modeled as

(32) $$R_{i,t}^{s}=\sigma_{s}^{2}\exp\left(-k_{s}u_{i,t}^{s}\right)I,\;u_{i,t}^{s}\in \left[0,1\right].$$

where $\sigma_{s}^{2}$ is the reference measurement-noise variance, and $k_{s}$ controls the sensitivity to the allocated sensing resource. Hence, a larger $u_{i,t}^{s}$ corresponds to lower measurement uncertainty.

The EKF prediction is [23]

(33) $$\begin{array}{cccc} {\hat{x}}_{i,k,t|t-1} & =F{\hat{x}}_{i,k,t-1|t-1},\; & \Sigma_{i,k,t|t-1} & =F\Sigma_{i,k,t-1|t-1}F^{T}+Q. \end{array}$$

and the measurement Jacobian is

(34) $$H_{i,k,t}={\left.\frac{\partial h_{i,k}\left(x\right)}{\partial x}\right|}_{x={\hat{x}}_{i,k,t|t-1}}.$$

The corresponding Kalman gain is [23]

(35) $$K_{i,k,t}=\Sigma_{i,k,t|t-1}H_{i,k,t}^{T}{\left(H_{i,k,t}\Sigma_{i,k,t|t-1}H_{i,k,t}^{T}+R_{i,t}^{s}\right)}^{-1}.$$

The measurement update is

(36) $$\begin{array}{cc} {\hat{x}}_{i,k,t|t} & ={\hat{x}}_{i,k,t|t-1}+K_{i,k,t}\left[z_{i,k,t}-h_{i,k}\left({\hat{x}}_{i,k,t|t-1}\right)\right]. \end{array}$$

(37) $$\begin{array}{cc} \Sigma_{i,k,t|t} & =\left(I-K_{i,k,t}H_{i,k,t}\right)\Sigma_{i,k,t|t-1}. \end{array}$$

The Joseph covariance form may alternatively be used when required for numerical robustness.

The position-estimation error is

(38) $$e_{i,k,t}^{\mathrm{loc}}={∥p_{i,k,t}-{\hat{p}}_{i,k,t}∥}_{2},\;p_{i,k,t}=\left[\begin{array}{c} x_{i,k,t}\\ y_{i,k,t} \end{array}\right],\;{\hat{p}}_{i,k,t}=\left[\begin{array}{c} {\hat{x}}_{i,k,t|t}\\ {\hat{y}}_{i,k,t|t} \end{array}\right].$$

while the covariance trace

(39) $$e_{i,k,t}^{\mathrm{cov}}=\mathrm{Tr}\left(\Sigma_{i,k,t|t}\right)$$

served as an uncertainty metric in the reward. The estimated state ${\hat{x}}_{i,k,t|t}$ and covariance $\Sigma_{i,k,t|t}$ are subsequently included in the HDRL-ISAC observations, enabling mobility- and uncertainty-aware beamforming, RIS configuration, association, power allocation, and sensing-resource control.

### 2.9. Mobility Model

To generate temporally correlated user trajectories, we adopt a Gauss–Markov mobility model [24,25]. The model provides smooth trajectories while retaining stochastic variations in user speed and movement direction, which is appropriate for evaluating mobility-aware RIS-assisted ISAC systems.

Let $v_{n}$ and $\theta_{n}$ denote the speed and direction of movement of a user at time slot *n*, respectively. Their evolution is modeled as follows:

(40) $$\begin{array}{cc} v_{n} & =\alpha v_{n-1}+\left(1-\alpha \right)\bar{v}+\sqrt{1-\alpha^{2}}\;v_{\mathrm{rand},n}. \end{array}$$

(41) $$\begin{array}{cc} \theta_{n} & =\alpha \theta_{n-1}+\left(1-\alpha \right)\bar{\theta }+\sqrt{1-\alpha^{2}}\;\theta_{\mathrm{rand},n}. \end{array}$$

where α∈[0,1] is the temporal correlation coefficient, $\bar{v}$ and $\bar{\theta }$ are the mean speed and mean direction, respectively, and $v_{\mathrm{rand},n}$ and $\theta_{\mathrm{rand},n}$ are zero-mean Gaussian random variables.

The corresponding two-dimensional position is updated according to

(42) $$\begin{array}{cc} x_{n} & =x_{n-1}+v_{n}\cos\left(\theta_{n}\right)\Delta t, \end{array}$$

(43) $$\begin{array}{cc} y_{n} & =y_{n-1}+v_{n}\sin\left(\theta_{n}\right)\Delta t. \end{array}$$

A larger value of α produces trajectories with a stronger temporal correlation and smoother motion [26,27], whereas smaller values result in more rapidly varying movement. Unless otherwise specified, the simulations use α=0.75 and user speeds in the range of 5–50 km/h.

The Gauss–Markov model was used to generate the ground-truth mobility trajectories, whereas the constant-velocity state model in (29) was used by the EKF as a computationally tractable local prediction model. This distinction allows the evaluation to capture the model mismatch between the actual user motion and the estimator while maintaining a lightweight state-tracking mechanism.

### 2.10. Problem Formulation

The objective is to jointly optimize the BS beamforming configuration, RIS phase configuration, user association, transmit-power allocation, and sensing-resource allocation to improve communication, energy efficiency, interference management, and localization performance while satisfying the practical network constraints defined in Section 2. Accordingly, the underlying resource optimization problem is formulated as a multi-objective optimization problem:

(44) $$\begin{array}{cc} \max_{W,\;\Theta ,\;A,\;p,\;u^{s}}\;\; & [\frac{1}{U}\sum_{i=1}^{C}\sum_{k\in U_{i}\left(t\right)}EE_{i,k,t},\;\frac{1}{U}\sum_{i=1}^{C}\sum_{k\in U_{i}\left(t\right)}a_{i,k,t}R_{i,k,t},\\ \; & -\frac{1}{U}\sum_{i=1}^{C}\sum_{k\in U_{i}\left(t\right)}a_{i,k,t}I_{i,k,t}^{\mathrm{inter}},\;-\frac{1}{U}\sum_{i=1}^{C}\sum_{k\in U_{i}\left(t\right)}a_{i,k,t}\mathrm{Tr}\left(\Sigma_{i,k,t|t}\right)]. \end{array}$$

The optimization is subject to the following constraints:

(45a) $$\begin{array}{cccc} \sum_{k\in U_{i}\left(t\right)}a_{i,k,t}p_{i,k,t} & \leq P_{i}^{\max},\; & \; & \forall i,t, \end{array}$$

(45b) $$\begin{array}{cccc} \theta_{i,n,t} & \in Q_{B_{\mathrm{RIS}}},\; & \; & \forall i,n,t, \end{array}$$

(45c) $$\begin{array}{cccc} R_{i,k,t} & \geq R_{k}^{\min},\; & \; & \forall i,k,t:a_{i,k,t}=1, \end{array}$$

(45d) $$\begin{array}{cccc} I_{i}^{\mathrm{inter}}\left(t\right) & \leq \Gamma_{i},\; & \; & \forall i,t, \end{array}$$

(45e) $$\begin{array}{cccc} \sum_{i=1}^{C}a_{i,k,t} & =1,\; & \; & \forall k,t, \end{array}$$

(45f) $$\begin{array}{cccc} a_{i,k,t} & \in \{0,1\},\; & \; & \forall i,k,t, \end{array}$$

(45g) $$\begin{array}{cccc} P_{i,k}^{\min}\leq p_{i,k,t} & \leq P_{i,k}^{\max}, & \; & \forall i,k,t, \end{array}$$

(45h) $$\begin{array}{cccc} 0\leq u_{i,t}^{s} & \leq 1, & \; & \forall i,t. \end{array}$$

Constraint (45a) limits the aggregate transmit power of each BS. Constraint (45b) enforces the finite-resolution RIS hardware constraint defined by the phase set $Q_{B_{\mathrm{RIS}}}$. Constraint (45c) guarantees the minimum QoS requirement for every associated user.

The inter-cell interference constraint is expressed through (45d), where $I_{i}^{\mathrm{inter}}\left(t\right)$ denotes the aggregate inter-cell interference affecting the protected receivers in cell *i* and $\Gamma_{i}$ is the corresponding interference threshold. The association constraints in (45e,45f) ensure that every active user has one serving BS. The transmit power bounds in (45g) restrict each power-control variable to its feasible operating range, while (45h) restricts the cell-level sensing-resource allocation to the normalized interval [0,1].

The sensing-resource variable $u_{i,t}^{s}$ affects the measurement quality through the sensing-noise model in (32) and consequently influences the EKF posterior covariance $\Sigma_{i,k,t|t}$. Therefore, the formulation captures the communication–sensing trade-off by jointly optimizing communication resources and sensing allocation.

In the proposed hierarchical architecture, the cell-level sensing decision is generated by the master agent together with the RIS and beamforming configuration:

(46) $$a_{i,t}^{M}=\left(c_{i,t}^{\Theta },c_{i,t}^{\mathrm{BF}},u_{i,t}^{s},\delta_{i,t}^{\mathrm{nbr}}\right).$$

where $c_{i,t}^{\Theta }$ denotes the selected RIS codeword index, $c_{i,t}^{\mathrm{BF}}$ denotes the beamforming configuration index, $u_{i,t}^{s}$ is the sensing-resource allocation factor, and $\delta_{i,t}^{\mathrm{nbr}}$ represents the neighboring-cell coordination variable. The user-level association and transmit-power decisions are generated by the corresponding slave agents as

(47) $$a_{i,k,t}^{S}=\left(a_{i,k,t},p_{i,k,t},c_{i,k,t}^{\mathrm{UE}}\right).$$

where $c_{i,k,t}^{\mathrm{UE}}$ represents the user-level control variable defined in Section 3.2.

The multi-objective formulation in (44) is not directly optimized as a single weighted objective by the physical system. Instead, it defines the competing performance criteria that the learning framework seeks to balance. During policy optimization, the objectives were normalized and incorporated into the scalar reward defined in Section 3.4. The corresponding interference constraint is additionally handled through the constraint-aware execution mechanism and, where enabled, the adaptive Lagrangian multiplier.

The resulting problem is a mixed discrete–continuous and non-convex optimization problem. The discrete variables include user association and finite-resolution RIS/beamforming configuration indices, whereas the continuous variables include transmit powers, beamforming parameters, and sensing-resource allocation. Moreover, the objective and constraints are coupled through time-varying wireless channels, imperfect CSI, user mobility, inter-cell interference, and EKF localization uncertainty. The resulting optimization must therefore be solved repeatedly under dynamically changing network states.

These characteristics make direct exhaustive or conventional iterative optimization computationally demanding for real-time multi-cell operation. The proposed hierarchical CTDE-based multi-agent reinforcement learning framework therefore learns decentralized policies that approximate the solution of the above constrained multi-objective problem while enforcing the physical feasibility conditions during execution.

## 3. Proposed HDRL-ISAC Framework

This section presents the proposed HDRL-ISAC framework for coordinated resource optimization in multi-cell RIS-assisted ISAC networks. The framework adopts a centralized-training-and-decentralized-execution (CTDE) paradigm with a hierarchical master–slave multi-agent architecture. The master agents perform cell-level coordination of RIS configuration, beamforming, sensing resources, and neighboring-cell coordination, whereas the slave agents perform user-level association, power allocation, and fine beam selection. This hierarchical decomposition reduces the dimensionality of the joint decision space while retaining inter-cell coordination during centralized training. During deployment, only decentralized actors and compact neighboring-cell information are used, enabling local decision-making under dynamic user mobility and imperfect CSI.

### 3.1. Hierarchical CTDE Architecture

Let *C* denote the number of coordinated cells and let $U_{i}$ denote the set of active users associated with cell *i*. A master agent is deployed at each BS, while a slave agent is associated with each active user. The master agents perform cell-level coordination, whereas the slave agents perform user-level resource allocation.

During centralized training, the critics have access to the global observations and joint actions of the participating agents. This additional information allows the critics to account for inter-cell coupling and reduces the non-stationarity encountered by independently trained agents. However, the actors retain decentralized information during execution. Specifically, a master actor uses the local cell observation together with the latest valid neighboring-cell summary, whereas a slave actor uses its local user observation and the decision provided by its corresponding master.

The hierarchical decision process is therefore decomposed into two levels. At the cell level, the master determines the coarse coordination variables that influence the feasible operating region of the corresponding slave agents. At the user level, the slave agents select the association, transmit power, and fine beamforming actions conditioned on the master decision. The actor–critic network architecture used in the proposed HDRL-ISAC framework, including the hidden-layer configuration and output activations, is summarized in Appendix A (Table A1). The resulting hierarchical policy reduces direct coupling among user-level decisions while retaining the global coordination capability provided by CTDE.

### 3.2. State and Action Design

The proposed HDRL-ISAC framework employs a hierarchical state representation that contains communication, sensing, mobility, load, and neighboring-cell information. During centralized training, the global state is represented as

(48) $$s_{t}=\left[{\left\{{\hat{x}}_{i,k,t},{\tilde{G}}_{i},{\tilde{h}}_{i,k},d_{i,k}\right\}}_{i,k},z_{t}^{\mathrm{nbr}},q_{t}^{\mathrm{load}},a_{t-1}\right].$$

where the individual components provide complementary information for coordinated communication and sensing control.

The mobility state of user *k* in cell *i* is obtained from the EKF described in Section 2.8 and is given by

(49) $${\hat{x}}_{i,k,t}={\left[\begin{array}{cccc} {\hat{x}}_{i,k,t} & {\hat{y}}_{i,k,t} & {\hat{v}}_{x,i,k,t} & {\hat{v}}_{y,i,k,t} \end{array}\right]}^{T}.$$

The normalized BS–RIS channel matrix is denoted by ${\tilde{G}}_{i}$, while ${\tilde{h}}_{i,k}$ represents the normalized RIS–user cascaded-channel features derived from the equivalent communication channel in (7). The direct BS–user channel estimated from the pilot transmission is denoted by $d_{i,k}$.

The neighboring-cell summary $z_{t}^{\mathrm{nbr}}$ contains compact coordination information received from adjacent cells, including border-user mobility estimates, uncertainty information, cell load, and predicted inter-cell interference. The construction, delay handling, and fusion of these messages are described with the neighboring-cell communication procedures described in Section 4.8. This avoids the requirement of decentralized actors to exchange full CSI during execution.

The local load vector $q_{t}^{\mathrm{load}}$ summarizes the current traffic and resource state of the serving cell, including the number of active users, queue occupancy, and normalized resource utilization. The previous action $a_{t-1}$ provides a temporal context and helps the policy maintain consistency across consecutive decision intervals.

During centralized training, the critic observes the complete global state in (48). During decentralized execution, the master actor observes only its local cell information and the latest valid neighboring-cell summary. Each slave actor uses its local mobility and channel information together with the serving-cell indicators and the coordination decision received from its master.

The master actor performs cell-level coordination through

(50) $$a_{i,t}^{M}=\left(c_{i,t}^{\Theta },c_{i,t}^{\mathrm{BF}},u_{i,t}^{s},\delta_{i,t}^{\mathrm{nbr}}\right).$$

where $c_{i,t}^{\Theta }$ are the indices of the finite-resolution RIS phase-shift codebook defined in (5), $c_{i,t}^{\mathrm{BF}}$ is the coordinated beamforming codebook index, $u_{i,t}^{s}\in \left[0,1\right]$ is the sensing-resource allocation factor, and $\delta_{i,t}^{\mathrm{nbr}}$ denotes the neighboring-cell coordination variables, including the interference budget and compact coordination information exchanged with adjacent cells.

The corresponding master policy is

(51) $$a_{i,t}^{M}=\pi_{\theta_{i}}^{M}\left(o_{i,t}^{M}\right).$$

Based on the master decision, the slave agent allocates user-level resources according to

(52) $$a_{i,k,t}^{S}=\left(a_{i,k,t},p_{i,k,t},c_{i,k,t}^{\mathrm{ue}}\right).$$

where $a_{i,k,t}\in \left\{0,1\right\}$ denotes the association decision, $p_{i,k,t}$ is the transmit power, and $c_{i,k,t}^{\mathrm{ue}}$ is the fine beamforming codebook index. The slave policy is given by

(53) $$a_{i,k,t}^{S}=\pi_{\psi_{i,k}}^{S}\left(o_{i,k,t}^{S},a_{i,t}^{M}\right).$$

The discrete components are represented using a differentiable Gumbel–Softmax relaxation during training and are converted to a discrete codebook or association selections during execution. Continuous power and sensing variables are generated using the bounded continuous policy parameterizations described in Section 3.4.

### 3.3. Constraint-Aware Decentralized Action Enforcement

The proposed HDRL-ISAC framework does not rely solely on neural actors to satisfy the hard physical and resource constraints. Instead, actors generate candidate actions that are subsequently processed by a deterministic feasibility layer before being applied to the environment. The feasibility layer is executed locally at each BS using its local observations, the current policy output, and the latest valid neighboring-cell summary. This design separates policy generation from constraint enforcement and enables decentralized execution under imperfect CSI and time-varying inter-cell information.

The enforcement mechanism consists of four main operations: (i) one association by construction; (ii) decentralized inter-cell interference control through link-budget decomposition; (iii) local verification and repair of power, QoS, beamforming, and sensing decisions; and (iv) a deterministic fallback when no fully feasible configuration is available. The RIS phase action was directly selected from the predefined finite-resolution codebook.

#### 3.3.1. Exactly-One Association by Construction

Each user *k* was controlled by a single slave agent. Hence, this association is not independently generated by multiple BS-side agents. For user *k*, the reachable candidate serving-cell set is defined as

(54) $$C_{k,t}=\left\{i_{\mathrm{serv}}\left(k,t-1\right)\right\}\cup \left\{j\in N\left(i_{\mathrm{serv}}\left(k,t-1\right)\right):k\in B_{i↔j}\right\}.$$

where $i_{\mathrm{serv}}\left(k,t-1\right)$ denotes the serving BS of user *k* in the previous slot, N(i) is the neighboring-cell set of BS *i*, and $B_{i↔j}$ denotes the set of border users for which inter-cell coordination between BSs *i* and *j* is available. Thus, a user can remain with their current serving BS or be considered for a reachable neighboring cell.

The slave agent produces one association-score vector

(55) $$z_{k,t}={\left(z_{i,k,t}\right)}_{i\in C_{k,t}}.$$

During training, the discrete association decision is relaxed using the Gumbel–Softmax operation as follows:

(56) $${\tilde{a}}_{i,k,t}=\frac{\exp\left[\left(z_{i,k,t}+g_{i,k,t}\right)/\tau_{g}\right]}{\sum_{j\in C_{k,t}}\exp\left[\left(z_{j,k,t}+g_{j,k,t}\right)/\tau_{g}\right]},\;i\in C_{k,t}.$$

where $g_{i,k,t}$ are independently sampled Gumbel variables, and $\tau_{g}>0$ is the Gumbel–Softmax temperature. Consequently,

(57) $$\sum_{i\in C_{k,t}}{\tilde{a}}_{i,k,t}=1,$$

Therefore, the relaxed association remains on the probability simplex.

During execution, the candidate cells are first evaluated by the deterministic feasibility layer described below. Let $F_{k,t}\subseteq C_{k,t}$ denote the set of candidate cells for which an admissible configuration can be constructed while satisfying applicable power, QoS, and interference conditions. If $F_{k,t}\neq \emptyset$, the serving cell is selected as follows:

(58) $$i_{k}^{★}=\arg\max_{i\in F_{k,t}}z_{i,k,t},\;a_{i,k,t}=1\left(i=i_{k}^{★}\right).$$

If $F_{k,t}=\emptyset$, the previous serving cell $i_{\mathrm{serv}}\left(k,t-1\right)$ is retained according to the fallback mechanism in Section 3.3.5. Therefore, exactly one serving cell is maintained for every user and time slot:

(59) $$\sum_{i=1}^{C}a_{i,k,t}=1,\;\forall k,t.$$

The handover follows a deterministic proposal–admit–commit procedure. The user first proposes the highest-ranked feasible candidate. The target BS evaluates the corresponding configuration using its local state and the latest valid neighboring-cell information. The association is committed only when the candidate satisfies the required feasibility conditions; otherwise, the next-ranked candidate is examined. The source BS releases the user only after the target configuration has been admitted. Thus, a user is never simultaneously served by two BSs, and association changes cannot be independently committed by multiple BS-side agents.

#### 3.3.2. Decentralized Interference Enforcement via Budget Decomposition

The aggregate inter-cell interference observed by cell *i* is written as

(60) $$I_{i}^{\mathrm{inter}}\left(t\right)=\sum_{j\neq i}I_{j\to i}\left(t\right).$$

where the interference contribution from neighboring BS *j* to the users served by cell *i* is

(61) $$I_{j\to i}\left(t\right)=\sum_{k\in U_{i}\left(t\right)}a_{i,k,t}\sum_{m\in U_{j}\left(t\right)}a_{j,m,t}\;p_{j,m,t}{\left|h_{j\to i,k,t}^{\mathrm{eff},H}w_{j,m,t}\right|}^{2}.$$

where $U_{i}\left(t\right)$ is the set of users associated with cell *i*, $h_{j\to i,k,t}^{\mathrm{eff}}$ is the effective channel from BS *j* to user *k* in cell *i*, and $w_{j,m,t}$ is the beamforming vector used by BS *j* for user *m*.

To enable decentralized enforcement of the aggregate interference constraint

(62) $$I_{i}^{\mathrm{inter}}\left(t\right)\leq \Gamma_{i}.$$

The threshold $\Gamma_{i}$ is decomposed into non-negative pairwise interference budgets. Specifically,

(63) $$\delta_{j\to i,t}=\Gamma_{i}\omega_{j\to i,t},\;\omega_{j\to i,t}\geq 0,\;\sum_{j\in N(i)}\omega_{j\to i,t}=1.$$

Therefore,

(64) $$\sum_{j\in N(i)}\delta_{j\to i,t}=\Gamma_{i}.$$

The allocation weights $\omega_{j\to i,t}$ are generated by the master agent of cell *i* through a normalized output, such as a softmax layer, and form part of the neighboring-cell coordination variable $\delta_{i,t}^{\mathrm{nbr}}$ in the master action. The resulting interference grants are exchanged between neighboring BSs through the Xn-like interface as compact coordination information.

If a received interference grant is older than the maximum permitted information age $\Delta_{\max}$ or fails the associated validity check, the receiving BS uses a conservative equal-share budget

(65) $$\delta_{j\to i}^{\mathrm{def}}=\frac{\Gamma_{i}}{\left|N(i)\right|}.$$

Thus, the budget decomposition remains well-defined even when the latest coordination message is temporarily unavailable.

Each BS then verifies its outgoing interference contribution against the corresponding neighbor budgets. Specifically, BS *j* accepts its configuration only when

(66) $${\hat{I}}_{j\to i}\left(t\right)\leq \left(1-\epsilon_{I}\right)\delta_{j\to i,t},\;\forall i\in N\left(j\right).$$

where ${\hat{I}}_{j\to i}\left(t\right)$ is the execution-time estimate of the interference generated by BS *j* toward cell *i*, and $\epsilon_{I}\in \left[0,1\right)$ is a safety margin.

Summing the local conditions over all neighboring BSs gives

(67) $$\begin{array}{cc} {\hat{I}}_{i}^{\mathrm{inter}}\left(t\right)\; & =\sum_{j\in N(i)}{\hat{I}}_{j\to i}\left(t\right)\\ \; & \leq \left(1-\epsilon_{I}\right)\sum_{j\in N(i)}\delta_{j\to i,t}\\ \; & =\left(1-\epsilon_{I}\right)\Gamma_{i}\leq \Gamma_{i}. \end{array}$$

Hence, local compliance is sufficient to satisfy the aggregate inter-cell interference constraint with respect to the execution-time interference estimates, without requiring per-slot centralized negotiation among BSs.

The interference estimates are obtained from locally available transmit decisions together with the latest accepted neighboring-cell summary. Thus, the mechanism preserves decentralized execution while providing a deterministic safety margin against estimation and coordination uncertainty.

#### 3.3.3. Local Verification and Repair

After association selection, each BS evaluates the corresponding candidate configuration using its local state, policy outputs, and neighboring-cell coordination information. The transmit power of each associated user is first projected onto its admissible interval:

(68) $${\tilde{p}}_{i,k,t}=\Pi_{\left[P^{\min},P^{\max}\right]}\left(p_{i,k,t}\right).$$

where $\Pi_{\left[P^{\min},P^{\max}\right]}\left(\cdot \right)$ denotes Euclidean projection onto the specified interval.

Because multiple slave agents associated with the same BS may act independently, their individual power decisions can collectively exceed the BS-level power budget. Therefore, a cell-level scaling operation is applied as follows:

(69) $${\hat{p}}_{i,k,t}={\tilde{p}}_{i,k,t}\min\left\{1,\;\frac{P_{i}^{\max}}{\sum_{\ell }a_{i,\ell ,t}{\tilde{p}}_{i,\ell ,t}}\right\}.$$

which guarantees

(70) $$\sum_{k}a_{i,k,t}{\hat{p}}_{i,k,t}\leq P_{i}^{\max}.$$

If the scaling operation reduces the power of an admitted user to below $P^{\min}$, that candidate configuration is not accepted directly. Instead, alternative feasible power allocations and fine-beam configurations were examined during the admission test.

For a candidate serving cell, *i*, the configuration is admitted only if an admissible combination of transmit power and fine-beam index exists such that the following conditions hold simultaneously:

(71) $$\begin{array}{cccc} \; & \left(\mathrm{power}\right)\; & \; & P^{\min}\leq {\hat{p}}_{i,k,t}\leq P^{\max},\;\sum_{\ell }a_{i,\ell ,t}{\hat{p}}_{i,\ell ,t}\leq P_{i}^{\max},\\ \; & \left(\mathrm{QoS}\right)\; & \; & {\hat{R}}_{i,k,t}=W\log_{2}\left(1+{\hat{\gamma }}_{i,k,t}\right)\geq R_{k}^{\min}, \end{array}$$

(72) $$\begin{array}{cccc} \; & \; & & {\hat{R}}_{i,\ell ,t}\geq R_{\ell }^{\min},\;\forall \ell \in U_{i}\left(t\right), \end{array}$$

(73) $$\begin{array}{cccc} \; & \left(\mathrm{interference}\right)\; & \; & {\hat{I}}_{i\to j}\left(t\right)\leq \left(1-\epsilon_{I}\right)\delta_{i\to j,t},\;\forall j\in N\left(i\right). \end{array}$$

Condition (72) includes both the candidate user and the users already served by cell *i*. Consequently, admitting a new user is not permitted to degrade the estimated QoS of the existing users below their required thresholds.

If the QoS condition is violated, feasible power and fine-beam configurations are searched. If the interference condition is violated, lower-power or alternative-beam configurations are examined provided that the QoS conditions remain satisfied. A candidate cell for which no admissible configuration can be constructed is removed from $F_{k,t}$.

The sensing-resource allocation is generated by the master agent and is directly projected onto its feasible interval:

(74) $${\tilde{u}}_{i,t}^{s}=\Pi_{\left[0,1\right]}\left(u_{i,t}^{s}\right).$$

which guarantees

(75) $$0\leq {\tilde{u}}_{i,t}^{s}\leq 1.$$

The resulting sensing allocation determines the fraction of resources assigned to sensing and consequently affects the quality of the measurements used by the EKF-based tracking process.

The overall execution operation can therefore be represented by a deterministic feasibility operator:

(76) $$a_{t}^{\mathrm{exec}}=F_{t}\left(a_{t}^{\mathrm{RL}},{\hat{s}}_{t},z_{t}^{\mathrm{nbr}}\right).$$

where $a_{t}^{\mathrm{RL}}$ denotes the joint candidate action generated by the decentralized actors, ${\hat{s}}_{t}$ contains the locally estimated system quantities, and $z_{t}^{\mathrm{nbr}}$ is the latest accepted neighboring-cell summary. Unlike a conventional Euclidean projection, the operator $F_{t}$ may perform candidate selection, power scaling, codebook selection, and discrete repair; hence, it is referred to as a feasibility operator rather than a purely geometric projection.

#### 3.3.4. RIS and Beamforming Codebook Enforcement

The master agent selects the RIS phase configuration using the predefined finite-resolution RIS codebook. If $c_{i,t}^{\Theta }$ denotes the selected codeword index, the executed RIS configuration is

(77) $${\tilde{\Theta }}_{i,t}=\Theta^{\left(c_{i,t}^{\Theta }\right)},\;\Theta^{\left(c_{i,t}^{\Theta }\right)}\in Q_{B_{\mathrm{RIS}}}.$$

where $Q_{B_{\mathrm{RIS}}}$ is the predefined $B_{\mathrm{RIS}}$-bit RIS phase codebook. Thus, the executed RIS configuration automatically satisfies the prescribed finite phase resolution without requiring a continuous-to-discrete projection.

Similarly, the master agent selects the coarse coordinated beamforming codeword, while the slave agent selects the corresponding user-specific fine-beam index. The feasibility layer rejects or replaces a fine-beam choice when it violates the QoS or interference conditions in (72) and (73). Therefore, beam selection remains consistent with the hierarchical master–slave action decomposition.

#### 3.3.5. Fallback When No Feasible Configuration Exists

If

(78) $$F_{k,t}=\emptyset ,$$

the user retains its previous serving cell $i_{\mathrm{serv}}\left(k,t-1\right)$, thereby preserving the exactly-one association property. The serving BS then attempts a conservative fallback configuration using the lowest-interference available beam and the minimum admissible transmission power.

If this configuration still violates the applicable execution-time interference or QoS conditions, transmission to user *k* is deferred for the current slot:

(79) $${\hat{p}}_{i,k,t}=0.$$

The association is retained, but the deferred transmission is counted as a QoS outage event. In this exceptional case, the per-user lower power bound is relaxed only for the deferred slot because no admissible transmission configuration exists. The event is explicitly recorded and included in the QoS-outage statistics.

Accordingly, the feasibility layer distinguishes between an admitted transmission, for which the applicable power, QoS, interference, RIS, and association constraints are enforced, and a deferred transmission, which is explicitly identified as an infeasible service event rather than being treated as a successful constraint-satisfying transmission.

#### 3.3.6. Scope of the Enforcement Guarantee

The deterministic guarantees of the feasibility layer hold with respect to the quantities available at execution time, including the estimated CSI, EKF-predicted border-user states, local transmit decisions, and the latest accepted neighboring-cell summaries. Under imperfect CSI, user mobility, and stale neighboring-cell information, the actual physical interference and achievable rate can deviate from their execution-time estimates.

To reduce the resulting mismatch, interference enforcement uses the safety margin $\epsilon_{I}$, while the measured interference is incorporated into the adaptive Lagrangian mechanism through $\lambda_{I}$ in (85). Thus, the deterministic feasibility layer provides instantaneous protection with respect to the available execution information, whereas the adaptive interference penalty provides additional learning-time pressure against persistent constraint violations.

The effect of the information age and imperfect neighboring-cell information was evaluated in the corresponding theoretical and simulation analyses. Therefore, the constraint-enforcement mechanism should not be interpreted as guaranteeing the exact physical constraints under arbitrary CSI or communication errors; rather, it provides deterministic feasibility with respect to the information available at the execution time.

Overall, the feasibility layer separates policy generation from hard constraint enforcement. Neural actors generate candidate hierarchical actions, whereas deterministic local operations select, repair, or reject configurations according to applicable constraints. This design preserves decentralized execution while enforcing exactly-one association, BS transmit-power limits, finite-resolution RIS operation, sensing-resource bounds, QoS requirements, and inter-cell interference budgets whenever an admissible configuration exists.

### 3.4. Reward Function and Policy Optimization

The HDRL-ISAC framework jointly optimizes communication and sensing objectives using a normalized scalar reward with adaptive interference constraint guidance. The four primary objectives, namely energy efficiency, achievable rate, inter-cell interference suppression, and localization accuracy, are scalarized using nonnegative weights. The objective components are normalized before scalarization, whereas the hard physical constraints are enforced separately by the deterministic feasibility layer described in Section 3.3. The normalization operator is defined as follows:

(80) $$N\left(x\right)=\mathrm{clip}\left(\frac{x-x_{\min}}{x_{\max}-x_{\min}+\varepsilon },0,1\right).\;$$

where ε>0 is a small constant introduced for numerical stability. The normalization statistics were obtained offline from representative training scenarios and remained fixed during training and evaluation.

The scalar objective reward is defined as

(81) $$\begin{array}{cc} r_{t}= & \;\eta_{1}\frac{1}{U}\sum_{i,k}N\left(EE_{i,k,t}\right)+\eta_{2}\frac{1}{U}\sum_{i,k}N\left(R_{i,k,t}\right)\\ \; & +\eta_{3}\left[1-\frac{1}{U}\sum_{i,k}N\left(I_{i,k,t}^{\mathrm{inter}}\right)\right]\\ \; & +\eta_{4}\left[1-\frac{1}{U}\sum_{i,k}N\left(\mathrm{Tr}\left(\Sigma_{i,k,t}^{\mathrm{pos}}\right)\right)\right]-\lambda_{\mathrm{HO}}\frac{1}{U}\sum_{k}H_{k}\left(t\right).\; \end{array}$$

where $\eta_{1},\ldots ,\eta_{4}\geq 0$ are the scalarization weights associated with energy efficiency, achievable rate, inter-cell interference suppression, and localization accuracy, respectively. The term $\lambda_{\mathrm{HO}}\geq 0$ penalizes association changes through the handover indicator $H_{k}\left(t\right)$, where $\Sigma_{i,k,t}^{\mathrm{pos}}$ denotes the position-error covariance obtained using the EKF.

The scalarization weights satisfy

(82) $$\eta_{j}\geq 0,\;\sum_{j=1}^{4}\eta_{j}=1.$$

which fixes the scale of the four-objective scalarization while allowing their relative importance to vary. Therefore, the selected weight vector represents a design preference rather than a universally optimal weighting.

#### 3.4.1. Reward-Weight Sensitivity Analysis

The influence of the scalarization weights is evaluated by varying one weight at a time while jointly renormalizing the complete four-dimensional weight vector to preserve (82). Specifically, for a varied component, *m*, with test value *v*, the unnormalized weights are defined as

(83) $${\tilde{\eta }}_{m}\left(v\right)=v,\;{\tilde{\eta }}_{j}\left(v\right)=\eta_{j}^{0},\;j\neq m.$$

and the actual weights used during training are

(84) $$\eta_{j}\left(v\right)=\frac{{\tilde{\eta }}_{j}\left(v\right)}{\sum_{\ell =1}^{4}{\tilde{\eta }}_{\ell }\left(v\right)},\;j=1,\ldots ,4.$$

Thus, each sensitivity experiment preserves $\sum_{j=1}^{4}\eta_{j}=1$ while varying the relative emphasis of the selected objective. The handover penalty $\lambda_{\mathrm{HO}}$ is treated separately and is not included in this four-term normalization. The resulting configurations were evaluated using the same network, channel, mobility, and training settings, and the corresponding results are reported in Appendix B.

#### 3.4.2. Adaptive Interference Constraint

To provide adaptive constraint guidance during policy optimization, an interference Lagrange multiplier $\lambda_{I}$ was introduced. The Lagrangian-augmented reward is defined as

(85) $$r_{t}^{L}=r_{t}-\lambda_{I}\left({\bar{I}}_{t}^{\mathrm{inter}}-\Gamma \right).$$

where ${\bar{I}}_{t}^{\mathrm{inter}}$ denotes the measured average inter-cell interference, and Γ is the prescribed interference threshold.

The multiplier is updated using

(86) $$\lambda_{I}\leftarrow {\left[\lambda_{I}+\eta_{\lambda }\left({\hat{I}}^{\mathrm{inter}}-\Gamma \right)\right]}_{0}^{\lambda_{\max}}.$$

where $\eta_{\lambda }$ is the multiplier learning rate [28], ${\hat{I}}^{\mathrm{inter}}$ is the estimated interference level over the current update window, and ${\left[\cdot \right]}_{0}^{\lambda_{\max}}$ denotes projection onto $\left[0,\lambda_{\max}\right]$. When the estimated interference exceeds the prescribed threshold, $\lambda_{I}$ increases and strengthens the corresponding penalty. Conversely, when the interference remains below the threshold, the multiplier decreases toward zero.

The adaptive Lagrangian term provides constraint guidance during policy optimization, whereas the deterministic feasibility layer in Section 3.3 remains responsible for execution-time enforcement of the applicable hard physical constraints. The handover penalty provides a dense learning signal for association changes. The resulting ping-pong handover rate is instead evaluated explicitly using the PPHR metric defined in Equation (101).

#### 3.4.3. Centralized Observation, Joint Action, and Policy

Within the CTDE, the centralized critic has access to global observations and joint actions during training, whereas decentralized actors operate using their local observations and the latest valid neighboring-cell information during execution:

(87) $$O_{t}={\left\{o_{i,t}^{M},o_{i,k,t}^{S}\right\}}_{i,k},\;A_{t}={\left\{a_{i,t}^{M},a_{i,k,t}^{S}\right\}}_{i,k}.$$

The joint hierarchical policy is factorized as

(88) $$\pi_{\theta ,\psi }\left(A_{t}|O_{t}\right)=\prod_{i}\pi_{\theta_{i}}^{M}\left(a_{i,t}^{M}|o_{i,t}^{M}\right)\prod_{i,k}\pi_{\psi_{i,k}}^{S}\left(a_{i,k,t}^{S}|o_{i,k,t}^{S},a_{i,t}^{M}\right).$$

#### 3.4.4. Hybrid Action Parameterization

Gumbel–Softmax reparameterization [29] was used during training for the discrete RIS codeword, beamforming codeword, and association decisions. For logits z, the relaxed sample is

(89) $$\tilde{z}=\mathrm{softmax}\left(\frac{z+g}{\tau_{g}}\right).$$

where g is an i.i.d. Gumbel noise vector, and $\tau_{g}$ is the Gumbel–Softmax temperature. During decentralized execution, the corresponding discrete candidate is selected deterministically and then processed by the feasibility layer.

For continuous transmit-power allocation [30], a squashed Gaussian policy [31] is used:

(90) $$p_{i,k,t}=P^{\min}+\frac{P^{\max}-P^{\min}}{2}\left[1+\tanh\left(\mu_{i,k,t}+\sigma_{i,k,t}\epsilon \right)\right],\;\epsilon ∼N\left(0,1\right).$$

which guarantees $P^{\min}\leq p_{i,k,t}\leq P^{\max}$. Similarly, the sensing-resource allocation is generated as

(91) $$u_{i,t}^{s}=\frac{1}{2}\left[1+\tanh\left(\mu_{i,t}^{s}+\sigma_{i,t}^{s}\epsilon \right)\right].$$

which satisfies $0\leq u_{i,t}^{s}\leq 1$. The sensing allocation affects the measurement quality according to

(92) $$R_{i,t}^{s}=\sigma_{s}^{2}\exp\left(-k_{s}u_{i,t}^{s}\right)I.$$

where $k_{s}$ controls the sensitivity of the modeled measurement uncertainty to the allocated sensing resource.

#### 3.4.5. Entropy-Regularized Actor–Critic

The entropy-regularized objective used for policy optimization [32,33] is

(93) $${\tilde{r}}_{t}=r_{t}^{L}+\tau H\left(\pi (\cdot |o_{t})\right),\;H\left(\pi (\cdot |o_{t})\right)=E_{a_{t}∼\pi }\left[-\log\pi (a_{t}|o_{t})\right].$$

where τ>0 denotes the entropy-temperature coefficient.

The centralized critic is trained using the entropy-regularized target

(94) $$y_{t}=r_{t}^{L}+\gamma E_{A_{t+1}∼\pi }\left[Q_{\bar{\phi }}\left(O_{t+1},A_{t+1}\right)-\tau \log\pi \left(A_{t+1}|O_{t+1}\right)\right].$$

where $Q_{\bar{\phi }}$ denotes the target centralized critic. The critic and actor objectives are

(95) $$\begin{array}{cc} L_{Q} & =E\left[{\left(Q_{\phi }\left(O_{t},A_{t}\right)-y_{t}\right)}^{2}\right]. \end{array}$$

(96) $$\begin{array}{cc} J_{\pi } & =E\left[Q_{\phi }\left(O_{t},A_{t}\right)-\tau \log\pi \left(A_{t}|O_{t}\right)\right]. \end{array}$$

Because the dual variables are updated more slowly than the actor parameters, the replay transitions retain the objective reward and constraint costs separately. Therefore, the Lagrangian reward in (85) is recomputed using the current multipliers when a mini-batch is sampled.

Polyak averaging is employed to stabilize the target networks:

(97) $${\bar{\theta }}_{\pi }\leftarrow \xi {\bar{\theta }}_{\pi }+\left(1-\xi \right)\theta_{\pi },\;{\bar{\theta }}_{Q}\leftarrow \xi {\bar{\theta }}_{Q}+\left(1-\xi \right)\theta_{Q}.$$

where ξ∈(0,1) denotes the Polyak coefficient. The update was applied to the corresponding master and slave target networks.

The entropy temperature is automatically adapted using

(98) $$L_{\tau }=E_{a_{t}∼\pi }\left[-\tau \left(\log\pi \left(a_{t}|o_{t}\right)+\bar{H}\right)\right],\;\tau \leftarrow \tau -\eta_{\tau }\nabla_{\tau }L_{\tau }.$$

where $\bar{H}$ is the target entropy, and $\eta_{\tau }$ is the temperature learning rate.

The resulting HDRL-ISAC framework combines hierarchical CTDE, constraint-aware decentralized execution, adaptive constraint guidance, and entropy-regularized policy optimization. The actors generate decentralized candidate actions, whereas the centralized critic accounts for inter-agent coupling during training. During execution, the feasibility layer enforces the applicable association, QoS, power, RIS, and interference constraints, whereas compact neighboring-cell summaries provide inter-cell coordination information. Therefore, the resulting hierarchical policy provides an approximate solution to the sequential counterpart of Problem (44). The complete learning and execution procedure is summarized in Algorithm 1, while the neighboring-cell message exchange procedures are presented separately in Algorithms 2 and 3.

#### 3.4.6. Ping-Pong Handover Definition and Evaluation Metric

The handover term in the original reward function, $1(a_{k,t}\neq a_{k,t-1})$, identifies any change in the serving BS. However, an association change does not necessarily represent a ping-pong handover, since a legitimate handover caused by user mobility may also change the serving cell only once. Therefore, we distinguish a general handover from a short-term back-and-forth handover and explicitly define the ping-pong event used in the evaluation.

A handover event for user *k* at slot *t* is defined as

(99) $${\mathrm{HO}}_{k,t}=1\;\left(a_{k,t}\neq a_{k,t-1}\right).$$

where $a_{k,t}$ denotes the serving BS of user *k* at slot *t*.

Let $t_{k}^{\left(n\right)}$ denote the slot at which the *n*-th handover of user *k* occurs, and let $c_{k}^{\left(n\right)}=a_{k,t_{k}^{\left(n\right)}}$ denote the BS selected after that handover. A ping-pong event is defined as a return to the BS occupied two handovers earlier within a predefined short-term window $T_{\mathrm{PP}}$. Accordingly,

(100) $${\mathrm{PP}}_{k}^{\left(n\right)}=1\;\left(c_{k}^{\left(n\right)}=c_{k}^{\left(n-2\right)}\;\wedge \;t_{k}^{\left(n\right)}-t_{k}^{\left(n-1\right)}\leq T_{\mathrm{PP}}\right).$$

where $T_{\mathrm{PP}}$ denotes the maximum intermediate residence time used to classify the A → B → A transition as a ping-pong event.

Thus, for a sequence

A→B→A,

the second handover is classified as a ping-pong event only when the user returns to cell *A* within $T_{\mathrm{PP}}$. In contrast, an isolated handover caused by sustained user mobility is counted as a handover but not as a ping-pong event.
**Algorithm 1** CTDE-Based Hierarchical HDRL-ISAC**Require:** Discount factor γ, entropy temperature τ, Gumbel–Softmax temperature $\tau_{g}$, target-network coefficient ξ, replay buffer D, centralized critic learning rate $\eta_{Q}$, master actor learning rate α, slave actor learning rate β, and mini-batch size *B* **Ensure:** Trained master and slave policies
  1:Initialize master actors ${\left\{\pi_{\theta_{i}}^{M}\right\}}_{i=1}^{C}$  2:Initialize slave actors ${\left\{\pi_{\psi_{i,k}}^{S}\right\}}_{i,k}$  3:Initialize centralized critic $Q_{\phi }\left(O_{t},A_{t}\right)$ and target critic $Q_{\bar{\phi }}\left(O_{t},A_{t}\right)$  4:Initialize replay buffer D, target networks, EKF states, RIS/beamforming codebooks, and neighbor-information buffers  5:**for** episode e=1,…,E **do**  6:      Reset user mobility, channel realizations, RIS configurations, and EKF states  7:      **for** time slot t=1,…,T **do**  8:            Acquire ISAC observations and update $\left\{{\hat{x}}_{i,k,t},\Sigma_{i,k,t}\right\}$ using the EKF  9:            Generate and receive neighboring-cell information using Algorithms 2 and 310:            Construct local master and slave observations $o_{i,t}^{M}$ and $o_{i,k,t}^{S}$11:            Generate master actions according to (50)12:            Generate slave actions conditioned on the corresponding master decisions according to (52)13:            Construct the joint action $A_{t}$ from all master and slave actions14:            Apply the deterministic feasibility layer to obtain the executable joint action $A_{t}^{\mathrm{exec}}$15:            Execute $A_{t}^{\mathrm{exec}}$ and obtain throughput, energy efficiency, interference, localization covariance, and reward measurements16:            Construct the centralized observation $O_{t}$ using the global training information17:            Store the transition $\left(O_{t},A_{t}^{\mathrm{exec}},r_{t},O_{t+1}\right)$ in D18:            **if** the replay buffer contains at least *B* transitions **then**19:                  Sample a mini-batch of *B* transitions from D20:                  Construct the next joint actions $A_{t+1}^{\prime }$ using the target master and slave policies21:                  Compute the entropy-regularized centralized Bellman target according to (94)22:                  Update the centralized critic by minimizing the temporal-difference loss in (95)23:                  Update all slave actors using the fixed learning rate β and the centralized critic according to (96)24:                  **if** tmod3=0 **then**25:                        Update all master actors using the fixed learning rate α and the centralized critic according to (96)26:                  **end if**27:                  Soft-update the target actor and critic networks according to (97)28:                  Update the entropy temperature according to (98)29:                  Update the interference Lagrange multiplier according to (85)30:            **end if**31:      **end for**32:**end for**33:**return** Trained master policies $\left\{\pi_{\theta_{i}}^{M}\right\}$ and slave policies $\left\{\pi_{\psi_{i,k}}^{S}\right\}$

**Algorithm 2** Inter-Cell Message Packing and Transmission**Require:** Neighbor set N(i), border-user sets $\left\{B_{i↔j}\right\}$, EKF estimates $\left\{{\hat{x}}_{b,t},\Sigma_{b,t}\right\}$, edge-load $\rho_{b,t}^{\mathrm{edge}}$, predicted interference $\eta_{b,t}^{\mathrm{int}}$, quantizer Q, rate limiter ${\mathrm{RL}}_{i}$  1:**for** each neighboring BS j∈N(i) **do**  2:      **for** each border user $b\in B_{i↔j}$ **do**  3:            Construct $m_{b,t}$ according to (158)  4:            Quantize ${\tilde{m}}_{b,t}\leftarrow Q\left(m_{b,t}\right)$  5:            **if** keyframe transmission is due **then**  6:                  Set $p_{b,t}\leftarrow \mathrm{Compress}\left({\tilde{m}}_{b,t}\right)$  7:                  Mark packet as keyframe  8:            **else**  9:                  Set $p_{b,t}\leftarrow \mathrm{Compress}\left({\tilde{m}}_{b,t}-{\tilde{m}}_{b,t}^{\mathrm{base}}\right)$10:                  Mark packet as delta update11:            **end if**12:            Generate ${\mathrm{crc}}_{b,t}$ and optional ${\mathrm{tag}}_{b,t}$13:            Form ${\mathrm{pkt}}_{b,t}$ according to (160)14:            **if** ${\mathrm{RL}}_{i}$ permits transmission **then**15:                  Transmit ${\mathrm{pkt}}_{b,t}$ to BS *j*16:                  Update the acknowledged/baseline state when the corresponding transmission is accepted17:            **else**18:                  Merge or defer the low-priority update19:            **end if**20:      **end for**21:**end for**

##### Ping-Pong Rate

**Figure 7:**
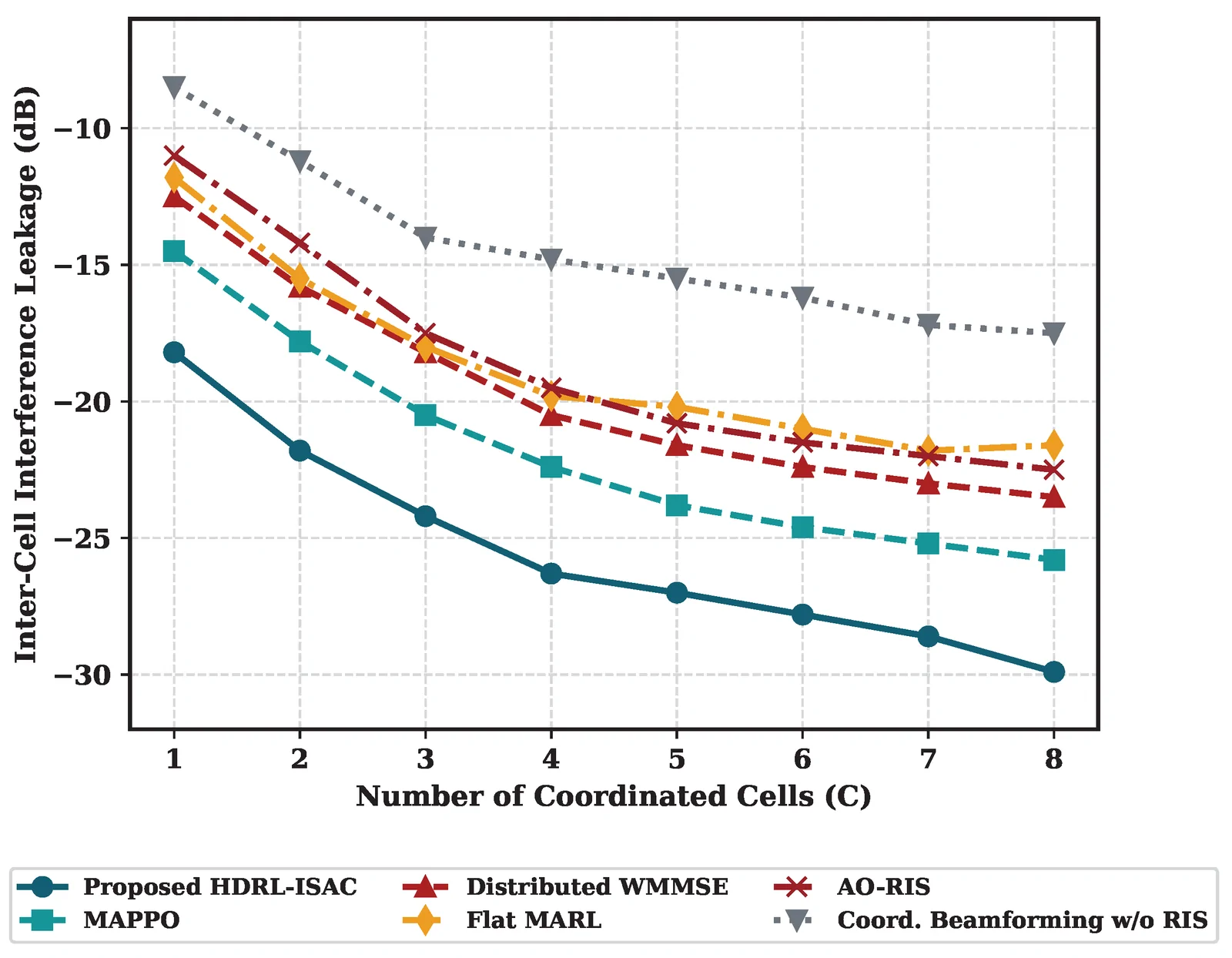
Inter-cell interference leakage versus the number of coordinated cells.

The ping-pong rate reported in Figure 7 is defined as the fraction of observed handover events that are classified as ping-pong events:

(101) $$\mathrm{PPHR}=\frac{\sum_{k=1}^{U}\sum_{n}{\mathrm{PP}}_{k}^{\left(n\right)}}{\sum_{k=1}^{U}\sum_{n}1\;\left[t_{k}^{\left(n\right)}\leq T_{\mathrm{eval}}\right]}\times 100\%.$$

where *U* is the number of active users and $T_{\mathrm{eval}}$ is the evaluation interval.

Hence, PPHR measures the proportion of handovers that result in a short-term return to a previously serving cell rather than measuring the overall frequency of association changes. This distinction prevents legitimate mobility-driven handovers from being directly treated as ping-pong events.

For the simulations, the ping-pong classification window was set to $T_{\mathrm{PP}}=5$ scheduling intervals. The same value is used for all compared methods to ensure consistent evaluation.
**Algorithm 3** Inter-Cell Message Reception and Staleness-Aware Aggregation**Require:** Neighbor set N(i), maximum information age $\Delta_{\max}$, staleness coefficient κ  1:Initialize neighbor buffer $Z_{i,t}^{\mathrm{nbr}}\leftarrow \emptyset$  2:**for** each neighboring BS j∈N(i) **do**  3:      **while** a packet from *j* is available **do**  4:            Parse payload, CRC/HMAC fields, timestamp, sequence number, and packet type  5:            **if** CRC or optional HMAC verification fails **then**  6:                  Discard packet  7:            **else**  8:                  Check the sequence number against the latest accepted sequence number  9:                  **if** packet is out of order **then**10:                        Discard packet11:                  **else**12:                        Reconstruct the quantized message from the keyframe or acknowledged delta baseline13:                        Compute$$\Delta_{j,t}=\frac{t_{i}-t_{j}^{\mathrm{gen}}}{\Delta t}$$14:                        **if** $\Delta_{j,t}>\Delta_{\max}$ **then**15:                              Discard the packet as stale16:                        **else**17:                              Dequantize ${\hat{x}}_{j,t}$, $\Sigma_{j,t}$, $\rho_{j,t}$, and $\eta_{j,t}$18:                              Propagate ${\hat{x}}_{j,t}$ and $\Sigma_{j,t}$ using the EKF prediction model for the elapsed information age19:                              Store the valid neighboring estimate and update the latest accepted sequence number20:                        **end if**21:                  **end if**22:            **end if**23:      **end while**24:**end for**25:**for** each border user *b* **do**26:      Compute$$\lambda_{u,t}=\frac{\rho_{u,t}}{\mathrm{Tr}(\Sigma_{u,t})+\epsilon }\exp\left(-\kappa \Delta_{u,t}\right)$$27:      Normalize$$\alpha_{u,t}=\frac{\lambda_{u,t}}{\sum_{v}\lambda_{v,t}}$$28:      Compute ${\hat{x}}_{b,t}^{\mathrm{agg}}$ using (166)29:    Compute $\Sigma_{b,t}^{\mathrm{agg}}$ using (167)30:**end for**31:Construct $z_{i,t}^{\mathrm{nbr}}$ according to (168)32:**return** $z_{i,t}^{\mathrm{nbr}}$

## 4. Theoretical Analysis

This section establishes the theoretical properties of the proposed HDRL-ISAC framework under the explicit boundedness, regularity, feasibility, and stochastic approximation assumptions. The analysis considers the entropy-regularized Bellman operator over the feasible hybrid action space, finite-resolution RIS implementation, delayed neighboring-cell information, hierarchical action decomposition, imperfect CSI, exact entropy-regularized policy improvement, and two-timescale master–slave learning dynamics.

The results provide operator-level convergence and conditional sensitivity guarantees for the constrained decision process. They do not imply global convergence or global optimality of the parameterized neural actor–critic implementation, which additionally depends on function approximation, stochastic optimization, exploration, replay sampling, and finite-sample effects. Practical convergence and performance are therefore evaluated empirically in Section 8.

### 4.1. Contraction of the Entropy-Regularized Bellman Operator over the Feasible Hybrid Action Space

The proposed HDRL-ISAC framework operates over a hybrid action space containing discrete RIS codebook and user-association decisions and continuous beamforming and transmit-power variables. Let the implementable action space at state *s* be

(102) $$A_{P}\left(s\right)=\Pi_{s}\left(A\right).$$

where $\Pi_{s}\left(\cdot \right)$ maps the original action space onto actions satisfying the finite-resolution RIS, transmit-power, interference, association, and QoS constraints. The analysis is performed directly over $A_{P}\left(s\right)$; the convexity and differentiability of $\Pi_{s}\left(\cdot \right)$ are not required, but the feasible set is assumed measurable and nonempty for every admissible state.

For a stationary feasible policy, π(a|s), the hybrid action measure is

(103) $$d\mu \left(a\right)=d\mu_{D}\left(a_{D}\right)d\mu_{C}\left(a_{C}\right).$$

where $\mu_{D}$ is the counting measure, and $\mu_{C}$ is the Lebesgue measure. The entropy-regularized policy-evaluation operator is [34]

(104) $$\begin{array}{cc} \left(T_{\tau }^{\pi }Q\right)\left(s,a\right) & =r\left(s,a\right)+\gamma E_{s^{\prime }∼P\left(\cdot |s,a\right)}\left[E_{a^{\prime }∼\pi \left(\cdot |s^{\prime }\right)}\left[Q\left(s^{\prime },a^{\prime }\right)-\tau \log\pi \left(a^{\prime }|s^{\prime }\right)\right]\right]. \end{array}$$

where τ>0 and 0<γ<1. Assume that

(105) $$\left|r\left(s,a\right)\right|\leq R_{\max},\;\left|E_{a∼\pi (\cdot |s)}\left[\log\pi \left(a|s\right)\right]\right|<\infty .$$

and that $P(s^{\prime }|s,a)$ is stationary.

**Theorem** **1** (Contraction of the Entropy-Regularized Bellman Operator)**.** *Under the above assumptions, $T_{\tau }^{\pi }$ is a γ-contraction under the supremum norm:*

(106) $${∥T_{\tau }^{\pi }Q_{1}-T_{\tau }^{\pi }Q_{2}∥}_{\infty }\leq \gamma {∥Q_{1}-Q_{2}∥}_{\infty }.$$

*Consequently, it has a unique fixed point, $Q_{\tau }^{\pi }$, and the exact Bellman evaluation converges to this fixed point [35].*

**Proof.**  For any feasible (s,a), the reward and entropy terms cancel each other when the two operator evaluations are subtracted. Therefore,

(107) $$\begin{array}{cc} \left|\left(T_{\tau }^{\pi }Q_{1}\right)\left(s,a\right)-\left(T_{\tau }^{\pi }Q_{2}\right)\left(s,a\right)\right|\; & \leq \gamma E_{s^{\prime },a^{\prime }}\left[|Q_{1}\left(s^{\prime },a^{\prime }\right)-Q_{2}\left(s^{\prime },a^{\prime }\right)|\right]\\ \; & \leq \gamma ∥Q_{1}-Q_{2}{∥}_{\infty }. \end{array}$$

Taking the supremum and applying the Banach fixed-point theorem proves the result. In particular,

(108) $$Q^{\left(n+1\right)}=T_{\tau }^{\pi }Q^{\left(n\right)}\;⟹\;Q^{\left(n\right)}\to Q_{\tau }^{\pi }.$$

The result directly applies to the implementable feasible action space and does not require the convexity of that space. It concerns exact fixed-policy evaluation and not the convergence of the parameterized neural critic. □

### 4.2. Conditional Performance Bound Under Finite-Resolution RIS Quantization

Let $\Theta^{★}$ denote an ideal continuous RIS configuration, and let $\hat{\Theta }$ denote its finite-resolution codebook projection:

(109) $$\hat{\Theta }=\Pi_{\Theta }\left(\Theta^{★}\right).$$

Assume that the one-step reward is Lipschitz-continuous with respect to the RIS reflection matrix [36]:

(110) $$|r\left(s,a,\Theta_{1}\right)-r\left(s,a,\Theta_{2}\right)|\leq L_{\Theta }{∥\Theta_{1}-\Theta_{2}∥}_{F}.$$

And define the maximum quantization error as

(111) $$\delta_{\Theta }=\underset{\Theta^{★}\in A_{\Theta }}{\sup}{∥\Theta^{★}-\hat{\Theta }∥}_{F}.$$

For this result, the transition kernel was assumed to be unchanged by RIS quantization for a fixed state and action, with any transition effect absorbed into the reward model.

**Theorem** **2** (Conditional Value Bound under Finite-Resolution RIS Quantization)**.** *For a fixed feasible policy,*

(112) $$|V_{\Theta^{★}}^{\pi }\left(s\right)-V_{\hat{\Theta }}^{\pi }\left(s\right)|\leq \frac{L_{\Theta }\delta_{\Theta }}{1-\gamma },\;\forall s.$$

**Proof.**  Equation (110) gives the one-step deviation

(113) $$|r\left(s,a,\Theta^{★}\right)-r\left(s,a,\hat{\Theta }\right)|\leq L_{\Theta }\delta_{\Theta }.$$

Summing this bounded perturbation over the discounted horizon yields

(114) $$|V_{\Theta^{★}}^{\pi }\left(s\right)-V_{\hat{\Theta }}^{\pi }\left(s\right)|\leq \sum_{t=0}^{\infty }\gamma^{t}L_{\Theta }\delta_{\Theta }=\frac{L_{\Theta }\delta_{\Theta }}{1-\gamma }.$$

Thus, increasing RIS phase resolution reduces $\delta_{\Theta }$ and tightens the conditional bound. □

### 4.3. Conditional Value Bound Under Delayed Neighboring-Cell Information

Let $z_{i,t}^{\mathrm{nbr}}$ and $z_{i,t-d_{t}}^{\mathrm{nbr}}$ denote the current and delayed neighboring-cell information, respectively. Assume bounded temporal variation,

(115) $$∥z_{i,t}^{\mathrm{nbr}}-z_{i,t-d_{t}}^{\mathrm{nbr}}∥\leq L_{z}d_{t},\;0\leq d_{t}\leq d_{\max}.$$

And Lipschitz reward sensitivity,

(116) $$|r\left(s,a,z_{1}^{\mathrm{nbr}}\right)-r\left(s,a,z_{2}^{\mathrm{nbr}}\right)|\leq L_{s}∥z_{1}^{\mathrm{nbr}}-z_{2}^{\mathrm{nbr}}∥.$$

The transition model is assumed common to the current- and delay-information comparisons.

**Theorem** **3** (Conditional Value Bound under Delayed Neighboring-Cell Information)**.** *For a fixed feasible policy,*

(117) $${∥V_{\mathrm{delay}}^{\pi }-V_{\mathrm{current}}^{\pi }∥}_{\infty }\leq \frac{L_{s}L_{z}d_{\max}}{1-\gamma }.$$

**Proof.**  Combining (115) and (116) gives

(118) $$|r_{\mathrm{delay}}-r_{\mathrm{current}}|\;\leq \;L_{s}L_{z}d_{t}\;\leq \;L_{s}L_{z}d_{\max}.$$

Discounted accumulation therefore gives

(119) $${∥V_{\mathrm{delay}}^{\pi }-V_{\mathrm{current}}^{\pi }∥}_{\infty }\leq \sum_{t=0}^{\infty }\gamma^{t}L_{s}L_{z}d_{\max}=\frac{L_{s}L_{z}d_{\max}}{1-\gamma }.$$

This bound characterizes the direct value sensitivity to stale neighboring-cell information; additional policy-induced action changes were evaluated experimentally. □

### 4.4. Local Approximation Error of the Hierarchical Decomposition

The proposed hierarchy decomposes the joint decision into master-level and slave-level actions. Let the continuous action vector be

(120) $$x={\left[\begin{array}{cc} x_{M}^{T} & x_{S}^{T} \end{array}\right]}^{T}.$$

And let $x^{★}$ and $x_{H}$ denote a locally optimal centralized continuous action and its hierarchical counterpart, respectively. The discrete RIS-codebook and association decisions remain in the feasible policy class but are not covered by the differentiability argument.

Assume that the reward has an $L_{r}$-Lipschitz gradient:

(121) $$∥\nabla_{x}r\left(s,x_{1}\right)-\nabla_{x}r\left(s,x_{2}\right)∥\leq L_{r}∥x_{1}-x_{2}∥.$$

And that

(122) $$\nabla_{x}r\left(s,x^{★}\right)=0.$$

Define

(123) $$\varepsilon_{H}=\underset{s}{\sup}∥x_{H}\left(s\right)-x^{★}\left(s\right)∥.$$

For the discounted interpretation, the centralized and hierarchical actions are assumed to induce the same state-transition distribution over the comparison horizon.

**Theorem** **4** (Local Approximation Bound of the Hierarchical Decomposition)**.** *Under the above assumptions,*

(124) $$|r\left(s,x^{★}\right)-r\left(s,x_{H}\right)|\;\leq \;\frac{L_{r}}{2}{∥x_{H}-x^{★}∥}^{2}.$$

*And consequently,*

(125) $$|J\left(\pi^{★}\right)-J\left(\pi_{H}\right)|\;\leq \;\frac{L_{r}\varepsilon_{H}^{2}}{2(1-\gamma )}.$$

**Proof.**  The $L_{r}$-smoothness inequality gives

(126) $$|r\left(s,x_{H}\right)-r\left(s,x^{★}\right)|\;\leq \;\frac{L_{r}}{2}{∥x_{H}-x^{★}∥}^{2}.$$

Because the first-order term vanishes by (122). Using the definition of $\varepsilon_{H}$ and summing over the discounted horizon yields (125). The result is therefore a local continuous-action approximation bound rather than a global optimality guarantee over the complete hybrid action space. □

### 4.5. Conditional Value Sensitivity Under Imperfect CSI

Let the estimated channel be

(127) $$\hat{H}=H+E.$$

with bounded estimation error

(128) $${∥E∥}_{F}\leq \varepsilon_{\mathrm{CSI}}.$$

Assume the one-step reward is Lipschitz-continuous with respect to the channel state:

(129) $$|r\left(s,a,H_{1}\right)-r\left(s,a,H_{2}\right)|\;\leq \;L_{H}{∥H_{1}-H_{2}∥}_{F}.$$

The transition kernel is assumed unchanged for fixed state and action, so the result characterizes direct policy-evaluation sensitivity.

**Theorem** **5** (Conditional Value Sensitivity under Imperfect CSI)**.** *Under *(128)* and *(129)*,*

(130) $${∥V_{H}^{\pi }-V_{\hat{H}}^{\pi }∥}_{\infty }\leq \frac{L_{H}\varepsilon_{\mathrm{CSI}}}{1-\gamma }.$$

**Proof.**  From (127) and (128),

$$∥H-\hat{H}{∥}_{F}\;\leq \;\varepsilon_{\mathrm{CSI}}.$$

And therefore

$$|r\left(s,a,H\right)-r\left(s,a,\hat{H}\right)|\;\leq \;L_{H}\varepsilon_{\mathrm{CSI}}.$$

Discounted accumulation gives

(131) $${∥V_{H}^{\pi }-V_{\hat{H}}^{\pi }∥}_{\infty }\leq \sum_{t=0}^{\infty }\gamma^{t}L_{H}\varepsilon_{\mathrm{CSI}}=\frac{L_{H}\varepsilon_{\mathrm{CSI}}}{1-\gamma }.$$

Thus, the direct value sensitivity grows linearly with the CSI error. This does not constitute a global robustness guarantee for neural actor–critic training. □

### 4.6. Monotonic Improvement of Exact Entropy-Regularized Policy Iteration

Let $\pi_{k}$ be a stationary feasible policy on $A_{P}\left(s\right)$. Exact policy evaluation gives [37]

(132) $$Q_{\tau }^{\pi_{k}}=T_{\tau }^{\pi_{k}}Q_{\tau }^{\pi_{k}}.$$

with entropy-regularized value

(133) $$V_{\tau }^{\pi }\left(s\right)=E_{a∼\pi (\cdot |s)}\left[Q_{\tau }^{\pi }\left(s,a\right)-\tau \log\pi \left(a|s\right)\right].$$

The exact soft policy-improvement step is

(134) $$\pi_{k+1}\left(a|s\right)=\frac{\exp\;\left(Q_{\tau }^{\pi_{k}}\left(s,a\right)/\tau \right)}{\int_{A_{P}\left(s\right)}\exp\;\left(Q_{\tau }^{\pi_{k}}\left(s,\tilde{a}\right)/\tau \right)d\mu \left(\tilde{a}\right)}.$$

Provided that

(135) $$0<\int_{A_{P}\left(s\right)}\exp\;\left(Q_{\tau }^{\pi_{k}}\left(s,\tilde{a}\right)/\tau \right)d\mu \left(\tilde{a}\right)<\infty .$$

**Theorem** **6** (Monotonic Improvement of Exact Entropy-Regularized Policy Iteration)**.** *If the feasible action space is measurable, the entropy-regularized value is finite, the partition function in *(135)* is finite, and policy evaluation is exact, then*

(136) $$V_{\tau }^{\pi_{k+1}}\left(s\right)\;\geq \;V_{\tau }^{\pi_{k}}\left(s\right),\;\forall s.$$

*If, additionally, the entropy-regularized Bellman optimality operator is a contraction, exact soft policy iteration converges to the entropy-regularized optimal value over the considered centralized feasible policy class.*

**Proof.**  Define

(137) $$F_{Q}\left(s,\pi \right)=E_{a∼\pi (\cdot |s)}\left[Q(s,a)-\tau \log\pi (a|s)\right].$$

By the Gibbs variational principle, the Gibbs distribution in (134) maximizes $F_{Q_{\tau }^{\pi_{k}}}\left(s,\pi \right)$. Hence,

(138) $$F_{Q_{\tau }^{\pi_{k}}}\left(s,\pi_{k+1}\right)\geq F_{Q_{\tau }^{\pi_{k}}}\left(s,\pi_{k}\right).$$

which gives

(139) $$V_{\tau }^{\pi_{k+1}}\left(s\right)\geq V_{\tau }^{\pi_{k}}\left(s\right).$$

For completeness, the entropy-regularized optimality operator is

(140) $$\begin{array}{cc} \left(T_{\tau }^{★}Q\right)\left(s,a\right)\; & =r\left(s,a\right)+\gamma E_{s^{\prime }∼P\left(\cdot |s,a\right)}\left[\tau \log\int_{A_{P}\left(s^{\prime }\right)}\exp\;\left(\frac{Q(s^{\prime },\tilde{a})}{\tau }\right)d\mu \left(\tilde{a}\right)\right]. \end{array}$$

The log-sum-exp functional is non-expansive under the supremum norm, so

(141) $${∥T_{\tau }^{★}Q_{1}-T_{\tau }^{★}Q_{2}∥}_{\infty }\leq \gamma {∥Q_{1}-Q_{2}∥}_{\infty }.$$

Thus, the optimality operator has a unique fixed point and exact soft policy iteration converges to the corresponding entropy-regularized optimal value. These are exact operator-level results and do not imply global convergence of the practical neural actor–critic algorithm. □

### 4.7. Two-Timescale Analysis of the Hierarchical Master–Slave CTDE Learning

The hierarchical CTDE architecture decomposes the joint decision process into master-level cell coordination and slave-level user-specific resource allocation. This subsection analyzes an *idealized* constant-step-size projected two-timescale model of this hierarchy [38,39] and derives two separate, explicitly quantified results: a bound on the slave’s tracking error under scheduled master updates (Proposition 1) and a bound on the master’s stationarity gap (Proposition 2). They characterize how the errors depend on the step sizes, update schedule, noise, and bias.

Let θ∈Θ collect the master-policy parameters and let ψ∈Ψ collect the slave-policy parameters:

(142) $$\psi ={\left\{\psi_{i,k}\right\}}_{i=1,\ldots ,C;\;k\in U_{i}},$$

where Θ and Ψ are compact and convex parameter domains. The hierarchical joint policy is expressed as

(143) $$\pi_{\theta ,\psi }\left(a^{M},a^{S}|o\right)=\prod_{i=1}^{C}\pi_{\theta_{i}}^{M}\left(a_{i}^{M}|o_{i}^{M}\right)\prod_{i=1}^{C}\prod_{k\in U_{i}}\pi_{\psi_{i,k}}^{S}\left(a_{i,k}^{S}|o_{i,k}^{S},a_{i}^{M}\right),$$

and the master and slave agents maximize the entropy-regularized joint objective

(144) $$J_{\tau }\left(\theta ,\psi \right)=E_{\pi_{\theta ,\psi }}\left[\sum_{t=0}^{\infty }\gamma^{t}\left(r_{t}+\tau H\left(\pi_{\theta ,\psi }\left(\cdot |o_{t}\right)\right)\right)\right].$$

#### 4.7.1. Model and Indexing

Let $g_{S}=\partial_{\psi }J_{\tau }$ and $g_{M}=\partial_{\theta }J_{\tau }$ be the mean update directions, and let $\psi^{★}\left(\theta \right)\in \Psi$ satisfy $g_{S}\left(\theta ,\psi^{★}\left(\theta \right)\right)=0$. The reduced master objective is

(145) $$\bar{J}\left(\theta \right)=J_{\tau }\left(\theta ,\psi^{★}\left(\theta \right)\right).$$

With constant step sizes α,β>0 and master update interval $K_{\mathrm{upd}}\in N$ ($K_{\mathrm{upd}}=3$ in Algorithm 1 and Table 1), the idealized recursions at mini-batch update index *n* are

(146) $$\begin{array}{cc} \psi_{n+1} & =\Pi_{\Psi }\;\left[\psi_{n}+\beta \left(g_{S}\left(\theta_{n},\psi_{n}\right)+b_{n}^{S}+M_{n+1}^{S}\right)\right], \end{array}$$

(147) $$\begin{array}{cc} \theta_{n+1} & =\Pi_{\Theta }\;\left[\theta_{n}+\alpha \;I_{\left\{n\;\mathrm{mod}\;K_{\mathrm{upd}}=0\right\}}\left(g_{M}\left(\theta_{n},\psi_{n}\right)+b_{n}^{M}+M_{n+1}^{M}\right)\right], \end{array}$$

where $\Pi_{\Psi }$ and $\Pi_{\Theta }$ denote Euclidean projections onto Ψ and Θ, $I_{\left\{\cdot \right\}}$ is the indicator function, $b_{n}^{S},b_{n}^{M}$ are biases (critic approximation, replay), and $M_{n+1}^{S},M_{n+1}^{M}$ are mini-batch noise terms. The master changes only at indices $n=mK_{\mathrm{upd}}$, so $\theta_{n}$ is constant for $mK_{\mathrm{upd}}+1\leq n\leq \left(m+1\right)K_{\mathrm{upd}}$. Writing ${\bar{\theta }}_{m}=\theta_{mK_{\mathrm{upd}}}$,

(148) $${\bar{\theta }}_{m+1}=\Pi_{\Theta }\;\left[{\bar{\theta }}_{m}+\alpha \left(g_{M}\left({\bar{\theta }}_{m},\psi_{mK_{\mathrm{upd}}}\right)+b_{mK_{\mathrm{upd}}}^{M}+M_{mK_{\mathrm{upd}}+1}^{M}\right)\right],$$

so ${\bar{\theta }}_{m}$ is the master parameter in force when the *m*-th master update is computed, and the intervening slave updates do not change it. The ratios

(149) $$\rho =\frac{\alpha }{\beta },\;\rho_{\mathrm{eff}}=\frac{\alpha }{K_{\mathrm{upd}}\beta }=\frac{\rho }{K_{\mathrm{upd}}}$$

describe the nominal and effective per-cycle step-size separation.

**Assumption** **1.** *The following assumptions establish the regularity conditions for the theoretical analysis, covering slave-response stability (A1–A2), master-objective regularity (A3), and bounded stochastic noise and approximation bias (A4). Together, they support the derivation of the subsequent theoretical bounds.*

*(A1)*  *$\psi^{★}\left(\theta \right)\in \Psi$ for all θ∈Θ.*
*(A2)*  *(Slave regularity, on all of *Ψ*.) For all $\theta ,\theta^{\prime }\in \Theta$ and $\psi ,\psi^{\prime }\in \Psi$: $\langle \psi -\psi^{\prime },\;g_{S}\left(\theta ,\psi \right)-g_{S}\left(\theta ,\psi^{\prime }\right)\rangle \leq -\mu {∥\psi -\psi^{\prime }∥}^{2}$ and $∥g_{S}\left(\theta ,\psi \right)-g_{S}\left(\theta ,\psi^{\prime }\right)∥\leq L∥\psi -\psi^{\prime }∥$ with 0<μ≤L; moreover, $∥g_{S}\left(\theta ,\psi \right)-g_{S}\left(\theta^{\prime },\psi \right)∥\leq L_{\theta }∥\theta -\theta^{\prime }∥$.*
*(A3)*  *(Master regularity.) $∥g_{M}∥\leq G_{M}$ on Θ×Ψ; $g_{M}$ is jointly $L_{g}$-Lipschitz; $\bar{J}$ is differentiable and satisfies the envelope identity $\nabla \bar{J}\left(\theta \right)=g_{M}\left(\theta ,\psi^{★}\left(\theta \right)\right)$; and $\Delta_{J}=\sup_{\Theta }\bar{J}-\inf_{\Theta }\bar{J}<\infty$.*
*(A4)*  *(Noise and bias.) $b_{n}^{S},b_{n}^{M}$ are $F_{n}$-measurable with $∥b_{n}^{S}∥\leq \epsilon_{S}$, $∥b_{n}^{M}∥\leq \epsilon_{M}$; $E\left[M_{n+1}^{S}|F_{n}\right]=E\left[M_{n+1}^{M}|F_{n}\right]=0$; $E[∥M_{n+1}^{S}{∥}^{2}|F_{n}]\leq \nu_{S}^{2}$ and $E[∥M_{n+1}^{M}{∥}^{2}|F_{n}]\leq \nu_{M}^{2}$.*

**Step-size conditions.** $0<\beta \leq \mu /L^{2}$ and $K_{\mathrm{upd}}\;\beta \mu \leq 4$ (for Proposition 1), and additionally $\alpha \leq 1/(2L_{J})$ (for Proposition 2), with $L_{J}$ defined in Lemma 1. Since μ≤L, $\beta \leq \mu /L^{2}$ implies βμ≤1, so $K_{\mathrm{upd}}\beta \mu \leq 4$ holds automatically for $K_{\mathrm{upd}}\leq 4$, including $K_{\mathrm{upd}}=3$.

**Remark** **1** (Remarks on the assumptions)**.** *(i) For $g_{S}=\partial_{\psi }J_{\tau }$ on the convex set *Ψ*, (A2) is equivalent to μ-strong concavity of $J_{\tau }\left(\theta ,\cdot \right)$ with an L-Lipschitz gradient; it also guarantees a unique zero $\psi^{★}\left(\theta \right)$. It is a regularity condition on the idealized model and is not claimed for neural policies. (ii) The envelope identity in (A3) is an *assumption*. It is implied, for example, when $J_{\tau }$ is $C^{2}$, $\psi^{★}\left(\theta \right)$ lies in the interior of *Ψ*, and $\nabla_{\psi \psi }^{2}J_{\tau }$ is nonsingular (the implicit-function theorem then makes $\psi^{★}$ $C^{1}$, and the envelope theorem applies since $\partial_{\psi }J_{\tau }\left(\theta ,\psi^{★}\left(\theta \right)\right)=0$). Its validity depends on the regularity of $\bar{J}$ and on the selected best-response branch, and it is not guaranteed merely because the slave gradient vanishes.*

**Lemma** **1** (Lipschitz best response and smooth reduced objective)**.** *Under (A1)–(A2), $\psi^{★}$ is $L_{\psi }$-Lipschitz with $L_{\psi }=L_{\theta }/\mu$. Under (A3), $\nabla \bar{J}$ is $L_{J}$-Lipschitz with $L_{J}=L_{g}\left(1+L_{\psi }\right)$.*

**Proof.**  Let $\Delta =\psi^{★}\left(\theta \right)-\psi^{★}\left(\theta^{\prime }\right)$. Since $g_{S}\left(\theta ,\psi^{★}\left(\theta \right)\right)=0$, (A2) gives ${\mu ∥\Delta ∥}^{2}\leq \langle \Delta ,g_{S}\left(\theta ,\psi^{★}\left(\theta^{\prime }\right)\right)\rangle =\langle \Delta ,\;g_{S}\left(\theta ,\psi^{★}\left(\theta^{\prime }\right)\right)-g_{S}\left(\theta^{\prime },\psi^{★}\left(\theta^{\prime }\right)\right)\rangle \leq ∥\Delta ∥L_{\theta }∥\theta -\theta^{\prime }∥$, so $∥\Delta ∥\leq \left(L_{\theta }/\mu \right)∥\theta -\theta^{\prime }∥$. By the envelope identity, $∥\nabla \bar{J}\left(\theta \right)-\nabla \bar{J}\left(\theta^{\prime }\right)∥\leq L_{g}(∥\theta -\theta^{\prime }∥+∥\Delta ∥)$. □

#### 4.7.2. Slave Tracking Under Scheduled Master Jumps

Let $e_{n}=\psi_{n}-\psi^{★}\left(\theta_{n}\right)$, $V_{n}=E[∥e_{n}{∥}^{2}]$, and $D^{2}={\left(G_{M}+\epsilon_{M}\right)}^{2}+\nu_{M}^{2}$.

**Proposition** **1** (Slave tracking)**.** *Under (A1)–(A4) and the step-size conditions, with q=1−βμ/4, for every n≥0:*

(150) $$V_{n}\leq q^{\;n}V_{0}+\bar{V},\;\bar{V}=\frac{5\beta \nu_{S}^{2}}{\mu }+\frac{15\;\epsilon_{S}^{2}}{\mu^{2}}+\frac{40\;L_{\psi }^{2}D^{2}}{\mu^{2}}\;\frac{\alpha^{2}}{K_{\mathrm{upd}}\;\beta^{2}}.$$

*The last term equals $40L_{\psi }^{2}D^{2}K_{\mathrm{upd}}\;\rho_{\mathrm{eff}}^{2}/\mu^{2}$ [38,39].*

**Proof.**  *Step 1 (slave step with $\theta_{n}$ fixed).* Let $u_{n}=\psi_{n+1}-\psi^{★}\left(\theta_{n}\right)$ and $g=g_{S}\left(\theta_{n},\psi_{n}\right)$. Since $\psi^{★}\left(\theta_{n}\right)\in \Psi$ is a fixed point of the non-expansive map $\Pi_{\Psi }$, $∥u_{n}∥\;\leq \;∥e_{n}+\beta g+\beta b_{n}^{S}+\beta M_{n+1}^{S}∥$. As $e_{n},g,b_{n}^{S}$ are $F_{n}$-measurable and $M_{n+1}^{S}$ has zero conditional mean, $E[∥u_{n}{∥}^{2}|F_{n}]\leq ∥e_{n}+\beta g+\beta b_{n}^{S}{∥}^{2}+\beta^{2}\nu_{S}^{2}$. By $g_{S}\left(\theta_{n},\psi^{★}\left(\theta_{n}\right)\right)=0$ and (A2), $\langle e_{n},g\rangle \leq -\mu {∥e_{n}∥}^{2}$ and $∥g∥\leq L∥e_{n}∥$, so $∥e_{n}{+\beta g∥}^{2}\leq \left(1-2\beta \mu +\beta^{2}L^{2}\right)∥e_{n}{∥}^{2}\leq \left(1-\beta \mu \right){∥e_{n}∥}^{2}$ for $\beta \leq \mu /L^{2}$, i.e., $∥e_{n}+\beta g∥\leq \left(1-\beta \mu /2\right)∥e_{n}∥$. Apply ${\left(A+B\right)}^{2}\leq \left(1+c\right)A^{2}+\left(1+1/c\right)B^{2}$ with c=βμ/2, use $\left(1+x\right){\left(1-x\right)}^{2}\leq 1-x$ for x=βμ/2, and $\beta^{2}\epsilon_{S}^{2}\leq \beta \epsilon_{S}^{2}/\mu$ (as β≤1/μ):

(151) $$E[∥u_{n}{∥}^{2}|F_{n}]\leq \left(1-\frac{\beta \mu }{2}\right){∥e_{n}∥}^{2}+s_{0},\;s_{0}=\frac{3\beta \epsilon_{S}^{2}}{\mu }+\beta^{2}\nu_{S}^{2}.$$

*Step 2 (master jump).* Write $e_{n+1}=u_{n}+d_{n+1}$ with $d_{n+1}=\psi^{★}\left(\theta_{n}\right)-\psi^{★}\left(\theta_{n+1}\right)$. Then $d_{n+1}=0$ unless $n\;\mathrm{mod}\;K_{\mathrm{upd}}=0$. At such *n*, by Lemma 1 and non-expansiveness of $\Pi_{\Theta }$, $∥d_{n+1}∥\leq L_{\psi }\alpha ∥g_{M}+b_{n}^{M}+M_{n+1}^{M}∥$, hence $E[∥d_{n+1}{∥}^{2}|F_{n}]\leq L_{\psi }^{2}\alpha^{2}D^{2}=:\omega^{2}$.

*Step 3 (recursion).* At master steps, apply the same Young inequality with c=βμ/4: (1+c)(1−βμ/2)≤1−βμ/4, 1+c≤5/4, and 1+1/c≤5/(βμ) (using βμ≤1). At non-master steps $d_{n+1}=0$ and the bound of Step 1 is smaller still. In both cases:

(152) $$V_{n+1}\leq q\;V_{n}+\frac{5}{4}s_{0}+I_{\left\{n\;\mathrm{mod}\;K_{\mathrm{upd}}=0\right\}}\;\Xi ,\;\Xi =\frac{5\omega^{2}}{\beta \mu }.$$

*Step 4 (unrolling).* Iterating, $V_{n}\leq q^{n}V_{0}+\sum_{j<n}q^{\;n-1-j}\left(\frac{5}{4}s_{0}+I_{\left\{j\;\mathrm{mod}\;K_{\mathrm{upd}}=0\right\}}\Xi \right)$. The $s_{0}$ part is at most $\frac{5}{4}s_{0}/\left(1-q\right)=5s_{0}/\left(\beta \mu \right)=5\beta \nu_{S}^{2}/\mu +15\epsilon_{S}^{2}/\mu^{2}$. The impulses occur at $j=mK_{\mathrm{upd}}$, and their weights, $q^{\;n-1-mK_{\mathrm{upd}}}$, form a geometric progression with ratio $q^{K_{\mathrm{upd}}}$ whose largest term is at most 1, so, for *every n* (including the step right after a jump), the impulse part is at most $\Xi /(1-q^{K_{\mathrm{upd}}})$. Set x=βμ/4; since $K_{\mathrm{upd}}x\leq 1$,

(153) $$1-q^{K_{\mathrm{upd}}}=1-{\left(1-x\right)}^{K_{\mathrm{upd}}}\geq 1-e^{-K_{\mathrm{upd}}x}\geq 1-\left(1-K_{\mathrm{upd}}x+\frac{{\left(K_{\mathrm{upd}}x\right)}^{2}}{2}\right)\geq \frac{K_{\mathrm{upd}}x}{2}=\frac{K_{\mathrm{upd}}\beta \mu }{8},$$

so $\Xi /\left(1-q^{K_{\mathrm{upd}}}\right)\leq \frac{5\omega^{2}}{\beta \mu }\cdot \frac{8}{K_{\mathrm{upd}}\beta \mu }=\frac{40L_{\psi }^{2}D^{2}\alpha^{2}}{K_{\mathrm{upd}}\beta^{2}\mu^{2}}$. Adding the three parts gives (150). □

#### 4.7.3. Master Stationarity Gap

Since Θ is a constrained feasible set, stationarity is quantified using the projected gradient mapping

$$G_{\alpha }\left(\theta \right)=\frac{\Pi_{\Theta }\;\left[\theta +\alpha \nabla \bar{J}\left(\theta \right)\right]-\theta }{\alpha },$$

where $\Pi_{\Theta }\left[\cdot \right]$ denotes projection onto Θ and α>0 is a step size. At interior points, the mapping reduces to $\nabla \bar{J}\left(\theta \right)$, while its vanishing characterizes constrained first-order stationary points.

**Proposition** **2** (Master stationarity gap)**.** *Under (A1)–(A4), the step-size conditions (including $\alpha \leq 1/(2L_{J})$), and with ${\bar{\theta }}_{m}=\theta_{mK_{\mathrm{upd}}}$ as in *(148)*:*

(154) $$\underset{T\to \infty }{\mathrm{lim sup}}\frac{1}{T}\sum_{m=0}^{T-1}E\left[∥G_{\alpha }\left({\bar{\theta }}_{m}\right){∥}^{2}\right]\leq 12L_{g}^{2}\;\bar{V}+12\;\epsilon_{M}^{2}+6\;\nu_{M}^{2}.$$

**Proof.**  Fix *m*, and write $\theta ={\bar{\theta }}_{m}$, $\theta^{+}={\bar{\theta }}_{m+1}=\Pi_{\Theta }\left[\theta +\alpha \hat{g}\right]$ with $\hat{g}=g_{M}\left(\theta ,\psi_{mK_{\mathrm{upd}}}\right)+b_{mK_{\mathrm{upd}}}^{M}+M_{mK_{\mathrm{upd}}+1}^{M}$ and $\mathrm{err}=\hat{g}-\nabla \bar{J}\left(\theta \right)$. Since θ∈Θ, the projection property $\langle y-\Pi_{\Theta }\left(y\right),\;\theta -\Pi_{\Theta }\left(y\right)\rangle \leq 0$ with $y=\theta +\alpha \hat{g}$ reads $∥\theta -\theta^{+}{∥}^{2}+\alpha \langle \hat{g},\theta -\theta^{+}\rangle \leq 0$, i.e., $\langle \hat{g},\theta^{+}-\theta \rangle \geq {∥\theta^{+}-\theta ∥}^{2}/\alpha$. Therefore, $\langle \nabla \bar{J},\theta^{+}-\theta \rangle \geq ∥\theta^{+}{-\theta ∥}^{2}/\alpha -∥\mathrm{err}∥∥\theta^{+}-\theta ∥\geq ∥\theta^{+}{-\theta ∥}^{2}/\left(2\alpha \right)-\frac{\alpha }{2}{∥\mathrm{err}∥}^{2}$. The ascent lemma for the $L_{J}$-smooth $\bar{J}$ and $\alpha \leq 1/(2L_{J})$ give

(155) $$\bar{J}\left(\theta^{+}\right)\geq \bar{J}\left(\theta \right)+\frac{1}{4\alpha }∥\theta^{+}{-\theta ∥}^{2}-\frac{\alpha }{2}{∥\mathrm{err}∥}^{2}.$$

By non-expansiveness of $\Pi_{\Theta }$, $∥\alpha G_{\alpha }\left(\theta \right)∥\leq ∥\theta^{+}-\theta ∥+\alpha ∥\mathrm{err}∥$; hence, $∥\theta^{+}{-\theta ∥}^{2}\geq \frac{\alpha^{2}}{2}∥G_{\alpha }{∥}^{2}-\alpha^{2}{∥\mathrm{err}∥}^{2}$ and

(156) $$\bar{J}\left({\bar{\theta }}_{m+1}\right)\geq \bar{J}\left({\bar{\theta }}_{m}\right)+\frac{\alpha }{8}∥G_{\alpha }\left({\bar{\theta }}_{m}\right){∥}^{2}-\frac{3\alpha }{4}{∥{\mathrm{err}}_{m}∥}^{2}.$$

Write ${\mathrm{err}}_{m}=\delta_{m}+b_{mK_{\mathrm{upd}}}^{M}+M_{mK_{\mathrm{upd}}+1}^{M}$ with $\delta_{m}=g_{M}\left({\bar{\theta }}_{m},\psi_{mK_{\mathrm{upd}}}\right)-g_{M}\left({\bar{\theta }}_{m},\psi^{★}\left({\bar{\theta }}_{m}\right)\right)$, so $∥\delta_{m}∥\leq L_{g}∥e_{mK_{\mathrm{upd}}}∥$ by (A3). Here, $\delta_{m}$ and $b_{mK_{\mathrm{upd}}}^{M}$ are $F_{mK_{\mathrm{upd}}}$-measurable and $M_{mK_{\mathrm{upd}}+1}^{M}$ has zero conditional mean, so $E[∥{\mathrm{err}}_{m}{∥}^{2}]\leq 2L_{g}^{2}V_{mK_{\mathrm{upd}}}+2\epsilon_{M}^{2}+\nu_{M}^{2}$. Taking expectations, summing over m=0,…,T−1, telescoping $\bar{J}$ (range $\Delta_{J}$), and using $V_{mK_{\mathrm{upd}}}\leq q^{mK_{\mathrm{upd}}}V_{0}+\bar{V}$ from Proposition 1:

(157) $$\frac{1}{T}\sum_{m<T}E\left[∥G_{\alpha }\left({\bar{\theta }}_{m}\right){∥}^{2}\right]\leq \frac{8\Delta_{J}}{\alpha T}+\frac{12L_{g}^{2}V_{0}}{T(1-q^{K_{\mathrm{upd}}})}+12L_{g}^{2}\bar{V}+12\epsilon_{M}^{2}+6\nu_{M}^{2},$$

where the first two terms are O(1/T). Letting T→∞ gives (154). □

##### Interpretation

The slave-tracking bound separates the effects of stochastic noise ($\propto \beta \nu_{S}^{2}$), approximation bias ($\propto \epsilon_{S}^{2}$), and master-policy motion ($\propto K_{\mathrm{upd}}\rho_{\mathrm{eff}}^{2}=\rho^{2}/K_{\mathrm{upd}}$). For a fixed update interval, the master-drift contribution decreases with the squared step-size ratio α/β but does not vanish when this ratio is held constant. The master-stationarity bound provides an asymptotic neighborhood bound whose residual depends on the slave-tracking error, master approximation bias, and master-gradient noise. The terms $\nu_{M}^{2}$ and $\epsilon_{M}^{2}$ are not scaled by α, as is typical for constant-step projected stochastic-gradient updates with unit batches.

These bounds characterize the idealized projected recursions and illustrate how the tracking error and stationarity gap depend on the step sizes, update schedule, stochastic noise, and approximation bias. They motivate the master–slave timescale design but do not establish convergence of the implemented neural actor–critic algorithm or identify an empirically optimal learning-rate ratio. The constants are worst-case quantities and are not intended to provide numerical predictions for the implemented system. Accordingly, the behavior of the implemented algorithm is assessed separately through the sensitivity experiments in Section 8.10.

### 4.8. Neighboring-Cell Information Exchange and Delay-Aware Execution

Efficient neighboring-cell coordination is required for inter-cell interference mitigation, mobility management, and stable decentralized decision-making. The proposed HDRL-ISAC framework therefore employs a lightweight asynchronous exchange of compact border-user statistics instead of full CSI or complete local observations. At each scheduling interval, each BS identifies border users relevant to neighboring-cell mobility or interference conditions and constructs

(158) $$m_{b,t}=\left[{\hat{x}}_{b,t},\mathrm{vech}\left(\Sigma_{b,t}\right),\rho_{b,t}^{\mathrm{edge}},\eta_{b,t}^{\mathrm{int}},{\mathrm{ts}}_{b,t},{\mathrm{seq}}_{b,t}\right],$$

where ${\hat{x}}_{b,t}$ and $\Sigma_{b,t}$ denote the EKF state estimate and covariance, respectively, while $\rho_{b,t}^{\mathrm{edge}}$, $\eta_{b,t}^{\mathrm{int}}$, ${\mathrm{ts}}_{b,t}$, and ${\mathrm{seq}}_{b,t}$ represent the edge-load, predicted-interference, timestamp, and sequence information. Thus, the message contains only the mobility, uncertainty, load, and interference statistics required for coordination, with dimension independent of the full antenna-by-user CSI matrix.

To reduce signaling overhead, the summary is quantized and delta-encoded as [40,41]

(159) $${\tilde{m}}_{b,t}=Q\left(m_{b,t}\right),\;p_{b,t}=\mathrm{Compress}\left({\tilde{m}}_{b,t}-{\tilde{m}}_{b,t}^{\mathrm{base}}\right).$$

where ${\tilde{m}}_{b,t}^{\mathrm{base}}$ is the acknowledged quantized baseline. Periodic full keyframes prevent permanent desynchronization after packet loss. The transmitted packet is

(160) $${\mathrm{pkt}}_{b,t}=\left[p_{b,t},{\mathrm{crc}}_{b,t},{\mathrm{tag}}_{b,t},{\mathrm{QoS}}_{b,t}\right].$$

where ${\mathrm{crc}}_{b,t}$ provides error detection, ${\mathrm{tag}}_{b,t}$ denotes an optional HMAC field, and ${\mathrm{QoS}}_{b,t}$ specifies packet priority [42]. A token-based rate limiter bounds control-plane signaling; when the budget is exhausted, low-priority updates are merged or deferred, while high-priority border-user information is retained.

#### 4.8.1. Asynchronous Xn Transport and Staleness-Aware Reception

The Xn/backhaul link is modeled with non-zero transport delay and jitter:

(161) $$\tau_{j\to i,t}={\bar{\tau }}_{j\to i}+\epsilon_{j\to i,t},\;t_{j\to i,t}^{\mathrm{arr}}=t_{j,t}^{\mathrm{gen}}+\tau_{j\to i,t}.$$

where ${\bar{\tau }}_{j\to i}$ and $\epsilon_{j\to i,t}$ denote the mean delay and random jitter, respectively. Using the timestamp and sequence number, the receiver computes the information age in scheduling intervals as

(162) $$\Delta_{j,t}=\frac{t_{i}-t_{j}^{\mathrm{gen}}}{\Delta t}.$$

After CRC/HMAC validation [43,44,45,46,47,48], the compressed message is reconstructed as

(163) $${\tilde{m}}_{b,t}=\mathrm{Decompress}\left(p_{b,t}\right)+{\tilde{m}}_{b,t}^{\mathrm{base}},$$

followed by inverse quantization. A message is accepted only when its sequence number is newer, and its age does not exceed the admissible staleness:

(164) $$\chi_{j,t}=1\left[q_{j,t}>q_{j\to i}^{\mathrm{last}}\right]1\left[\Delta_{j,t}\leq \Delta_{\max}\right].$$

Accordingly, out-of-order or excessively stale packets are discarded, whereas valid delayed information is retained and used without blocking local decisions. If no new valid packet arrives, the latest accepted summary is retained until its age exceeds $\Delta_{\max}$, after which its contribution is removed, and the local observation is used. Valid delayed mobility estimates are additionally propagated through the EKF prediction model to compensate for information age, with the associated uncertainty increasing with the prediction horizon.

For neighboring estimate, *u*, confidence is determined from the estimation covariance and information freshness:

(165) $$\lambda_{u,t}^{\mathrm{st}}=\frac{\rho_{u,t}}{\mathrm{Tr}(\Sigma_{u,t})+\epsilon }\exp\left(-\kappa \Delta_{u,t}\right),\;\alpha_{u,t}=\frac{\lambda_{u,t}^{\mathrm{st}}}{\sum_{v}\lambda_{v,t}^{\mathrm{st}}}.$$

where ϵ>0 prevents numerical instability, and κ>0 controls the staleness penalty. Thus, fresh and low-uncertainty information receives greater weight. The aggregated state and conservative mixture covariance are

(166) $${\hat{x}}_{b,t}^{\mathrm{agg}}=\sum_{u}\alpha_{u,t}{\hat{x}}_{u,t}.$$

(167) $$\Sigma_{b,t}^{\mathrm{agg}}=\sum_{u}\alpha_{u,t}\left[\Sigma_{u,t}+\left({\hat{x}}_{u,t}-{\hat{x}}_{b,t}^{\mathrm{agg}}\right){\left({\hat{x}}_{u,t}-{\hat{x}}_{b,t}^{\mathrm{agg}}\right)}^{T}\right].$$

This formulation captures both individual estimation uncertainty and disagreement among neighboring estimates.

The resulting border-user statistics are compressed into the neighborhood representation

(168) $$z_{i,t}^{\mathrm{nbr}}=\mathrm{Agg}\left({\left\{{\hat{x}}_{b,t}^{\mathrm{agg}},\Sigma_{b,t}^{\mathrm{agg}},{\bar{\rho }}_{b,t},{\bar{\eta }}_{b,t}\right\}}_{b}\right).$$

where ${\bar{\rho }}_{b,t}$ and ${\bar{\eta }}_{b,t}$ are the aggregated edge-load and interference indicators. This representation is provided to the master-agent observation and to the centralized critic during CTDE training.

#### 4.8.2. Two-Timescale Delay-Aware Execution

The hierarchical architecture separates fast local resource control from slower inter-cell coordination. Slave agents make user-level decisions from current local observations, whereas master agents use the latest valid neighboring summaries for cell-level coordination. Hence, a fresh Xn message is not required for every scheduling decision. The complete execution path is

(169) $$\begin{array}{c} \begin{array}{c} \mathrm{Message}\;\mathrm{Generation}\to \mathrm{Quantization}/\mathrm{Compression}\to \mathrm{Xn}\;\mathrm{Transmission}\to \mathrm{Delay}/\mathrm{Jitter}\\ \to \mathrm{Integrity}\;\mathrm{Check}\to \mathrm{Timestamp}/\mathrm{Sequence}\;\mathrm{Check}\to \mathrm{Staleness}\;\mathrm{Filtering}\\ \to \mathrm{Age}\;\mathrm{Compensation}\to \mathrm{Confidence}-\mathrm{Aware}\;\mathrm{Fusion}\to \mathrm{Neighborhood}\;\mathrm{Representation}\\ \to \mathrm{Local}/\mathrm{Hierarchical}\;\mathrm{Policy}\to \mathrm{Feasibility}\;\mathrm{Enforcement}. \end{array} \end{array}$$

When Xn information is delayed, the receiving BS continues using the latest accepted summary; therefore, backhaul delay affects information freshness rather than blocking the local decision path. The execution budget is strictly bounded by the control interval:

(170) $$T_{\mathrm{local}}\leq T_{\mathrm{ctrl}},\;T_{\mathrm{local}}=T_{\mathrm{pre}}+T_{\mathrm{actor}}^{M}+T_{\mathrm{actor}}^{S}+T_{\mathrm{decode}}+T_{\mathrm{proj}}+T_{\mathrm{FE}},$$

where the inter-cell communication delay $T_{\mathrm{Xn}}$ enters the system exclusively through the information age $\Delta_{j,t}$ evaluated in Equation (162). Thus, local execution is evaluated against the 1 ms scheduling interval, while Xn transport is handled entirely asynchronously through the staleness-aware mechanism. The detailed procedures are given in Algorithms 2 and 3; theoretical sensitivity to delayed information is analyzed in Section 4.3, with empirical delay results reported in Section 8.

## 5. Proposed Algorithm

This section describes the implementation of the proposed CTDE-based hierarchical HDRL-ISAC framework. Algorithm 1 summarizes the overall training procedure, while Algorithms 2 and 3 implement the inter-cell information exchange described in Section 4.8.

At each scheduling interval, ISAC observations are processed using the EKF to estimate the user mobility states and associated uncertainty. The resulting state estimates, local wireless information, and valid neighboring-cell summaries were incorporated into decentralized observations. Master agents then generate cell-level coordination decisions, whereas slave agents generate user-level association, transmit-power, and beam-selection decisions conditioned on the master action.

The resulting actions are passed through the deterministic feasibility layer described in Section 3.3. This step ensures that the executed actions satisfy the applicable RIS, association, transmit power, QoS, and interference constraints before interacting with the wireless environment.

During centralized training, critics exploit global CTDE observations and joint actions, whereas actors retain their decentralized information structure. The master and slave actor updates follow the constant-step-size two-timescale formulation of Section 4.7, where $\beta =2\times 10^{-4}$ denotes the fast slave learning rate, $\alpha =1\times 10^{-4}$ denotes the slower master learning rate, and the master actor updates once every $K_{\mathrm{upd}}=3$ slave updates.

### Algorithmic Execution and CTDE Workflow

**Figure 2:**
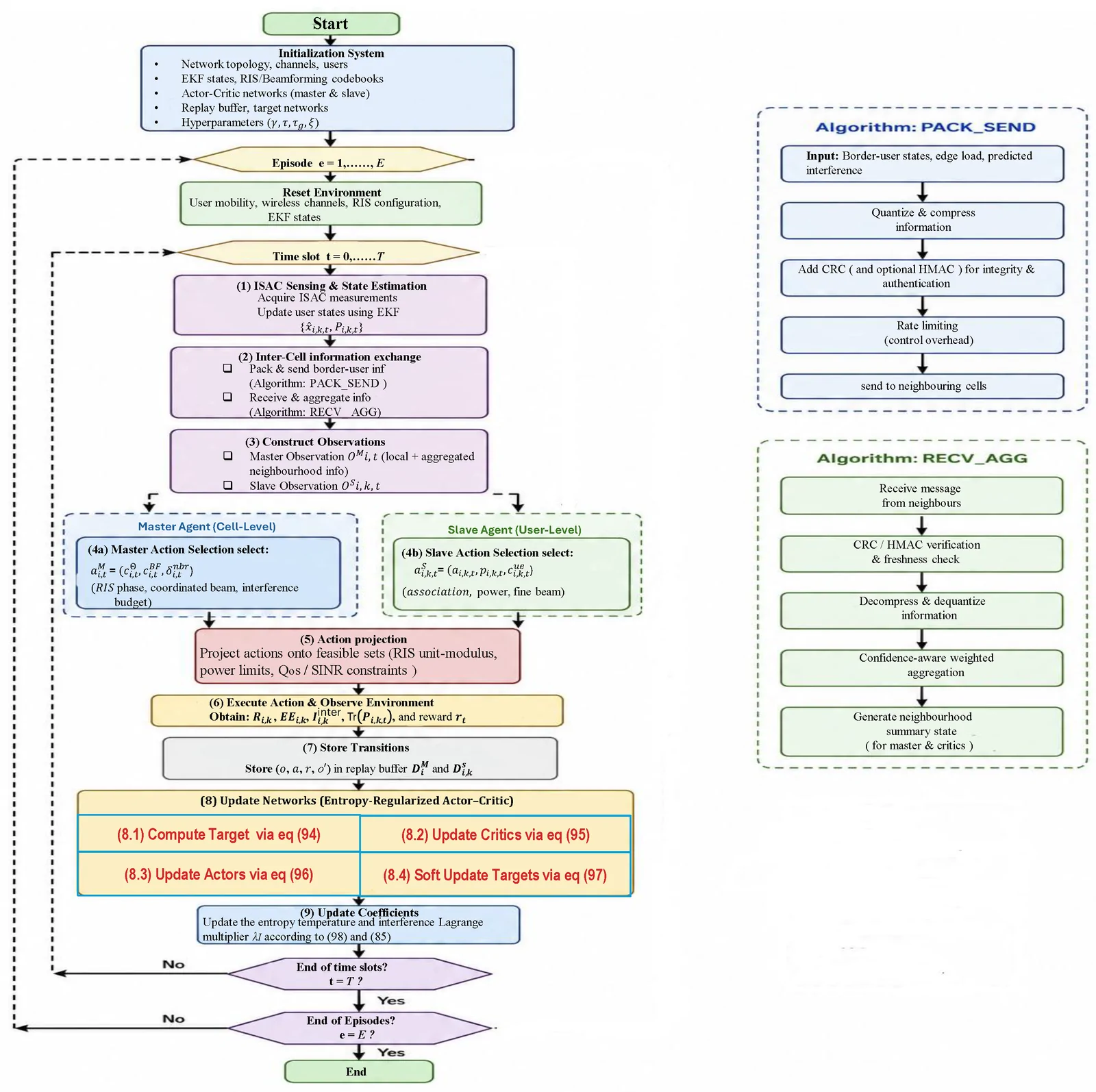
Overall execution and training workflow of the proposed CTDE-based hierarchical HDRL-ISAC framework, including ISAC state estimation, delay-aware neighboring-cell information exchange, hierarchical master–slave decision making, deterministic feasibility enforcement, and centralized training with decentralized execution.

Figure 2 illustrates the overall execution and training workflow of the proposed HDRL-ISAC framework. ISAC measurements are first processed through the EKF to obtain mobility states and uncertainty information. The neighboring-cell module then performs compact message exchange, freshness verification, delay compensation, and confidence-aware aggregation.

The resulting local and neighboring observations are supplied to the hierarchical master and slave actors. The master determines cell-level coordination variables, after which the slave agents generate user-level decisions conditioned on the master action. The deterministic feasibility layer subsequently converts the raw RL outputs into executable actions satisfying the applicable system constraints.

During centralized training, the global observations and joint actions are provided to the centralized critics, whereas the actors retain their decentralized information structures. The master and slave actor updates were performed on the respective learning timescales described in Section 4.7. During deployment, decentralized actors require only local observations and valid compact neighboring summaries.

## 6. Complexity and Communication Analysis

### 6.1. Computational Complexity

The computational complexity of the proposed HDRL-ISAC framework is determined by the online inference of the hierarchical master–slave policies, centralized actor–critic updates during training, and the deterministic feasibility operations used for action enforcement. Let $|U_{i}|$ denote the number of active users in cell *i*. For the master and slave networks, let ${\left\{d_{\ell }^{M}\right\}}_{\ell =0}^{L_{M}}$ and ${\left\{d_{\ell }^{S}\right\}}_{\ell =0}^{L_{S}}$ denote the layer dimensions, including input and output layers.

For fully connected networks, the dominant number of multiply–accumulate operations is proportional to the products of consecutive layer dimensions. Thus, the online actor-inference complexity per scheduling interval is

(171) $$O_{\inf}=O\;\left(\sum_{\ell =0}^{L_{M}-1}d_{\ell }^{M}d_{\ell +1}^{M}+\left|U_{i}\right|\sum_{\ell =0}^{L_{S}-1}d_{\ell }^{S}d_{\ell +1}^{S}\right).$$

For approximately uniform hidden-layer widths $d_{M}$ and $d_{S}$, this becomes

(172) $$O_{\inf}=O\;\left(L_{M}d_{M}^{2}+\left|U_{i}\right|L_{S}d_{S}^{2}\right).$$

Hence, the slave-inference cost scales linearly with the number of served users and becomes increasingly significant as user density increases.

During centralized training, the critics process global observations and joint actions. Let $B_{M}$ and $B_{S}$ denote the mini-batch sizes for the master and slave updates, respectively, and let ${\left\{d_{\ell }^{Q}\right\}}_{\ell =0}^{L_{Q}}$ denote the critic-layer dimensions. The critic-update complexity is approximately

(173) $$O_{\mathrm{critic}}=O\;\left(B_{M}\sum_{\ell =0}^{L_{Q}-1}d_{\ell }^{Q}d_{\ell +1}^{Q}+\left|U_{i}\right|B_{S}\sum_{\ell =0}^{L_{Q}-1}d_{\ell }^{Q}d_{\ell +1}^{Q}\right),$$

or, for approximately uniform critic width $d_{Q}$,

(174) $$O_{\mathrm{critic}}=O\;\left(B_{M}L_{Q}d_{Q}^{2}+\left|U_{i}\right|B_{S}L_{Q}d_{Q}^{2}\right).$$

The corresponding actor-update complexity during training is

(175) $$O_{\mathrm{actor}}=O\;\left(B_{M}\sum_{\ell =0}^{L_{M}-1}d_{\ell }^{M}d_{\ell +1}^{M}+\left|U_{i}\right|B_{S}\sum_{\ell =0}^{L_{S}-1}d_{\ell }^{S}d_{\ell +1}^{S}\right).$$

#### 6.1.1. RIS Codebook Execution

The RIS policy directly outputs a discrete codeword index $c_{i,t}^{\Theta }$ from the predefined finite codebook

(176) $$Q_{B}=\left\{\Theta^{\left(1\right)},\ldots ,\Theta^{\left(B_{\Theta }\right)}\right\}.$$

Therefore, no nearest-codeword search is required during online execution. The selected RIS configuration is obtained directly as

(177) $$\Theta_{i,t}=\Theta^{\left(c_{i,t}^{\Theta }\right)},$$

where the codebook is precomputed offline. The codeword-index selection itself is an indexed lookup with

(178) $$O_{\mathrm{RIS},\mathrm{select}}=O\left(1\right),$$

while constructing or applying the selected *N*-element RIS phase configuration requires

(179) $$O_{\mathrm{RIS},\mathrm{apply}}=O\left(N\right).$$

Consequently, the online RIS operation is

(180) $$O_{\mathrm{RIS}}=O\left(N\right),$$

when the selected phase vector is explicitly constructed or applied. For the adopted 3-bit phase resolution, $B_{\Theta }=2^{3}=8$, but the number of codewords does not introduce a nearest-codeword search cost because the policy directly selects the codeword index.

#### 6.1.2. Constraint-Aware Feasibility Enforcement

The deterministic feasibility layer evaluates candidate association, power, QoS, and interference constraints. If *C* denotes the number of candidate serving cells considered for each user, candidate screening has complexity

(181) $$O_{\mathrm{FE}}=O(|U_{i}\left|C\right),\;$$

apart from the cost of evaluating the underlying rate and interference quantities. The complete online computational cost is therefore

(182) $$O_{\mathrm{online}}=O_{\inf}+O_{\mathrm{RIS}}+O_{\mathrm{FE}}.\;$$

The centralized critics and actor updates are required only during training. Hence, decentralized deployment is dominated by hierarchical actor inference and deterministic feasibility enforcement, while training complexity is dominated by centralized actor–critic optimization.

### 6.2. Communication Overhead

The proposed CTDE framework exchanges compact neighboring-cell summaries rather than complete CSI or full channel matrices. Each border-user summary contains quantized EKF state and covariance information, edge-load and predicted-interference indicators, and packet metadata such as sequence and timestamp fields. Let $B_{\mathrm{msg}}$ denote the resulting message size in bytes, and let $N_{b}$ denote the average number of border-user summaries transmitted over one neighboring-cell link.

For the neighboring-cell graph G=(V,E), the total Xn signaling volume per scheduling interval is

(183) $$H_{\mathrm{Xn}}=\sum_{(i,j)\in E}N_{b}B_{\mathrm{msg}},\;$$

where directed links are counted separately when both transmission directions are active. The corresponding signaling rate is

(184) $$R_{\mathrm{Xn}}=\frac{H_{\mathrm{Xn}}}{\Delta t},\;$$

where Δt is the scheduling interval.

The proposed message-packing mechanism reduces signaling by exchanging compact statistical summaries instead of full CSI. Quantization, delta encoding, and rate limiting are applied before transmission, whereas packet validation and age-based rejection are performed at the receiver. The complete message generation, delay, jitter, and staleness-handling procedures are described in Section 4.8.

Within each cell, the master agent provides slave agents with compact coordination variables, including RIS codeword selections, beamforming configuration indices, sensing-control variables, and other bounded coordination information. Thus, the intra-cell control overhead scales approximately linearly with the number of active users:

(185) $$O_{\mathrm{ctrl}}=O(|U_{i}\left|\right).\;$$

A token-bucket rate limiter further bounds the control-plane signaling rate. In the simulation configuration, the maximum permitted Xn control-plane rate was set to the value reported in Table 1. The effect of Xn/backhaul delay and jitter on the decentralized performance are evaluated separately in Section 8.

## 7. Simulation Setup

The proposed HDRL-ISAC framework is evaluated in a multi-cell RIS-assisted ISAC network with C∈{3,8} wrap-around hexagonal cells. Each cell contains an M=8-antenna ULA BS and an N=128-element RIS serving multiple single-antenna mobile users. Unless otherwise stated, the carrier frequency and bandwidth are $f_{c}=28$ GHz and W=400 MHz, respectively. User mobility follows a Gauss–Markov model with control interval Δt=1 ms, memory correlation factor α=0.75, and velocity range v∈[5,50] km/h. The propagation environment uses distance-dependent path loss and 3GPP-inspired UMi spatial modeling with clustered Rician fading. The Rician factor, active-user count, and user index are denoted by $K_{R}$, $K_{u}$, and *k*, respectively.

The Rician channel model is

(186) $$h\left(d\right)=\sqrt{\beta (d)}\left(\sqrt{\frac{K_{R}}{K_{R}+1}}h_{\mathrm{LoS}}+\sqrt{\frac{1}{K_{R}+1}}h_{\mathrm{NLoS}}\right),$$

where $\beta \left(d\right)=C_{0}d^{-\alpha_{\mathrm{PL}}}$ is the large-scale channel gain. The adopted Rician factor is

(187) $$K_{R}=10^{K_{R,dB}/10}=10,\;K_{R,dB}=10\;\mathrm{dB},\;$$

and the active-user load is varied over $K_{u}\in \left[2,12\right]$ users per cell.

Imperfect CSI is modeled as

(188) $$\hat{H}=H+E,\;E∼\mathrm{CN}\left(0,\sigma_{\mathrm{CSI}}^{2}I\right),\;$$

with

(189) $${\mathrm{SNR}}_{\mathrm{CSI}}=\frac{1}{\sigma_{\mathrm{CSI}}^{2}},\;{\mathrm{SNR}}_{\mathrm{CSI}}\in \left[5,20\right]\;\mathrm{dB}.\;$$

ISAC AoA, ToA, and Doppler observations are fused by the EKF to obtain the kinematic state ${\hat{x}}_{i,k,t}$ and covariance $\Sigma_{i,k,t}$.

### 7.1. Learning Configuration

The proposed hierarchical CTDE architecture employs one master agent per BS and one slave agent per active UE. Master actors coordinate RIS codewords, beamforming, sensing-resource allocation, and neighboring-cell information, while slave actors perform user association, transmit-power, and user-level beamforming decisions conditioned on the master output. Discrete actions use Gumbel–Softmax during training and deterministic feasibility enforcement during execution; continuous power and sensing allocations use squashed Gaussian policies.

The principal learning parameters are τ=0.05, γ=0.99, and ξ=0.995 for the initial entropy temperature, discount factor, and Polyak smoothing coefficient, respectively. Training uses E=200 episodes with T=2000 interaction steps per episode, prioritized experience replay, a replay buffer of $D=2\times 10^{5}$ transitions, and mini-batch size B=128.

RIS operation uses $B_{\mathrm{RIS}}=3$-bit phase resolution, i.e., eight feasible phase levels per RIS element:

(190) $$2^{B_{\mathrm{RIS}}}=8.\;$$

The policy directly selects a predefined RIS codeword index as described in Section 2.1.

### 7.2. Xn/Backhaul Configuration

Neighboring BSs exchange compact border-user summaries through the Xn interface. Token-bucket rate limiting restricts the nominal signaling overhead to below 50 kB/s per neighboring link. The robustness study uses nominal one-way delays

(191) $${\bar{\tau }}_{j\to i}\in \left\{0,5,10,15,20\right\}\;\mathrm{ms},\;$$

with $\sigma_{\tau }=1.5$ ms, maximum accepted information age $\Delta_{\max}=25$ slots, and packet-loss rate $p_{\mathrm{loss}}=2\%$. Since Δt=1 ms, $\Delta_{\max}=25$ corresponds to 25 ms. The reported delay curves evaluate the specified transport-delay sweep; independent quantitative claims for jitter or packet-loss sensitivity require corresponding dedicated sweeps.

### 7.3. Evaluation Metrics

Performance was evaluated using network energy efficiency (EE, Mbits/J), 5th-percentile cell-edge throughput (Mbps), inter-cell interference leakage (dB), ping-pong handover rate (PPHR), training sample efficiency, and EKF covariance trace Tr(Σ). Learning results were averaged over ten independent random seeds and reported with 95% confidence intervals.

### 7.4. Baseline Methods

To evaluate the proposed HDRL-ISAC framework against both contemporary learning-based coordination schemes and conventional wireless optimization methods, the experiments consider the following baselines under identical channel realizations, mobility trajectories, RIS configurations, and resource constraints.

1. Coordinated Beamforming without RIS (CBF w/o RIS): A coordinated beamforming architecture in which the BSs serve mobile users without RIS-assisted passive phase control.
2. Alternating Optimization with RIS (AO-RIS): A classical block-coordinate optimization method that alternates between BS digital beamforming and RIS phase optimization. The resulting continuous RIS phase configuration is projected onto the discrete 3-bit RIS codebook. Since AO-RIS is an iterative offline optimization method rather than a policy-learning algorithm, its performance is reported using converged optimization results rather than RL training episodes.
3. Flat Multi-Agent Reinforcement Learning (Flat-MARL): A non-hierarchical CTDE multi-agent reinforcement learning architecture in which the agents jointly learn beamforming, RIS selection, user association, and power allocation without explicit master–slave decomposition.
4. Multi-Agent PPO (MAPPO): A cooperative multi-agent policy-optimization baseline employing a centralized value function during training and decentralized PPO actors during execution.
5. Distributed WMMSE with Discrete RIS Projection (WMMSE-RIS): A distributed weighted minimum mean-squared error (WMMSE) precoding scheme coordinated through inter-cell message exchange and combined with discrete RIS-codebook selection.

For learning-based methods, convergence was evaluated in terms of training episodes and the $N_{90}$ metric. In contrast, AO-RIS and WMMSE-RIS are iterative optimization methods and are therefore evaluated using their optimization iterations and converged performance rather than RL training episodes. This separation avoids an inappropriate comparison between policy-learning convergence and offline numerical optimization. All simulations and network training pipelines were conducted on an NVIDIA GeForce RTX A5000 GPU (22 GB VRAM) and an Intel Core i9-14900 K (24 cores, 3200 MHz) using Python 3.9.13 and PyTorch 2.0.1 with CUDA 11.7. simulation parameters are summarized in Table 1.

**Table 1:** Simulation, Training, and Baseline Parameters.

Parameter	Value
**System Parameters**	
Number of cells (*C*)	3/8
BS antennas (*M*)	8
RIS elements (*N*)	128
Carrier frequency ($f_{c}$)	28 GHz
Bandwidth (*W*)	400 MHz
Noise spectral density ($N_{0}$)	−174 dBm/Hz
Receiver noise figure	7 dB
Mobility model	Gauss–Markov ($\alpha_{\mathrm{mob}}=0.75$)
User speed (*v*)	5–50 km/h
CSI estimation SNR	5–20 dB
Rician factor ($K_{R,\mathrm{dB}}$)	10 dB
Rician factor ($K_{R}$)	10 (linear)
RIS phase resolution ($B_{\mathrm{RIS}}$)	3 bits
RIS codebook size ($B_{\Theta }$)	8 configurations
Maximum BS power ($P_{\max}$)	30 dBm (1 W)
Circuit power per user ($P_{c}$)	0.1 W
Interference threshold (Γ)	−90 dBm
**HDRL-ISAC Parameters**	
Optimizer	Adam
Master actor learning rate (α)	$1\times 10^{-4}$
Slave actor learning rate (β)	$2\times 10^{-4}$
Master update frequency	Every 3 slave updates
Critic learning rate ($\eta_{Q}$)	$2\times 10^{-4}$
Actor architecture	2 hidden layers, 256 units each
Critic architecture	2 hidden layers, 256 units each
Hidden-layer activation	ReLU
Replay buffer (|D|)	$2\times 10^{5}$ transitions
Mini-batch size (*B*)	128
Discount factor (γ)	0.99
Polyak update factor (ξ)	0.995
Target policy entropy ($\bar{H}$)	−3
Initial entropy temperature (τ)	0.05
Gumbel–Softmax temperature ($\tau_{g}$)	0.5
Entropy learning rate ($\eta_{\tau }$)	$3\times 10^{-4}$
Lagrangian learning rate ($\eta_{\lambda }$)	$10^{-2}$
Aggregation window ($W_{\mathrm{agg}}$)	2
Neighbor-message budget	<50 kB/s per neighbor
EKF process-noise covariance ($Q_{\mathrm{EKF}}$)	0.10 (normalized/model units)
Reward weights (η)	[0.30,0.25,0.30,0.15]
Handover penalty ($\lambda_{\mathrm{HO}}$)	0.15
**MAPPO Baseline Parameters**	
Optimizer	Adam
Actor architecture	2 hidden layers, 256 units each
Critic architecture	2 hidden layers, 256 units each
Activation function	ReLU
Actor learning rate ($\eta_{\pi }$)	$3\times 10^{-4}$
Critic learning rate ($\eta_{V}$)	$5\times 10^{-4}$
Discount factor (γ)	0.99
GAE parameter ($\lambda_{\mathrm{GAE}}$)	0.95
PPO clipping parameter ($\epsilon_{\mathrm{clip}}$)	0.20
Entropy coefficient ($c_{\mathrm{ent}}$)	0.01
Value-loss coefficient ($c_{V}$)	0.50
Optimization epochs per update	10
Mini-batch size	256
Gradient clipping norm	0.5
Observation normalization	Running mean and variance
Advantage normalization	Enabled
Reward normalization	Disabled
Evaluation actions	Deterministic policy
**WMMSE-RIS Baseline Parameters**	
Precoder initialization	MRT with power normalization
RIS initialization	Best feasible of 8 configurations
Precoder update	WMMSE receiver, weight, and precoder updates
RIS update	Discrete codebook search with fixed precoder
Optimization strategy	Alternating precoder/RIS updates
Relative stopping tolerance ($\epsilon_{\mathrm{WMMSE}}$)	$10^{-3}$
Maximum outer iterations ($I_{\max}$)	100
Power constraint	Enforce BS power limit
Interference constraint	Same applicable system threshold
**Communication and Robustness**	
Xn one-way delay (${\bar{\tau }}_{j\to i}$)	0/5/10/15/20 ms
Xn delay jitter ($\sigma_{\tau }$)	1.5 ms
Xn packet loss ($p_{\mathrm{loss}}$)	2%
Maximum information age ($\Delta_{\max}$)	25 slots (25 ms)
Staleness coefficient (κ)	0.15
Control interval ($T_{\mathrm{ctrl}}$)	1 ms
**Training and Evaluation**	
Training episodes (*E*)	200
Steps per episode (*T*)	2000
Replay sampling	Prioritized Experience Replay
Numerical stability (ε)	0.005
Random seeds	10 independent runs
Confidence interval	95%

## 8. Results and Discussion

The numerical evaluation benchmarks the proposed HDRL-ISAC framework against Flat-MARL, MAPPO, CBF without RIS, AO-RIS, and WMMSE-RIS under varying user loads, mobility conditions, RIS quantization levels, CSI quality, and Xn/backhaul transport delays. Unless otherwise stated, learning-based results use identical training budgets and random-seed settings.

### 8.1. Energy Efficiency and Convergence Behavior

**Figure 3:**
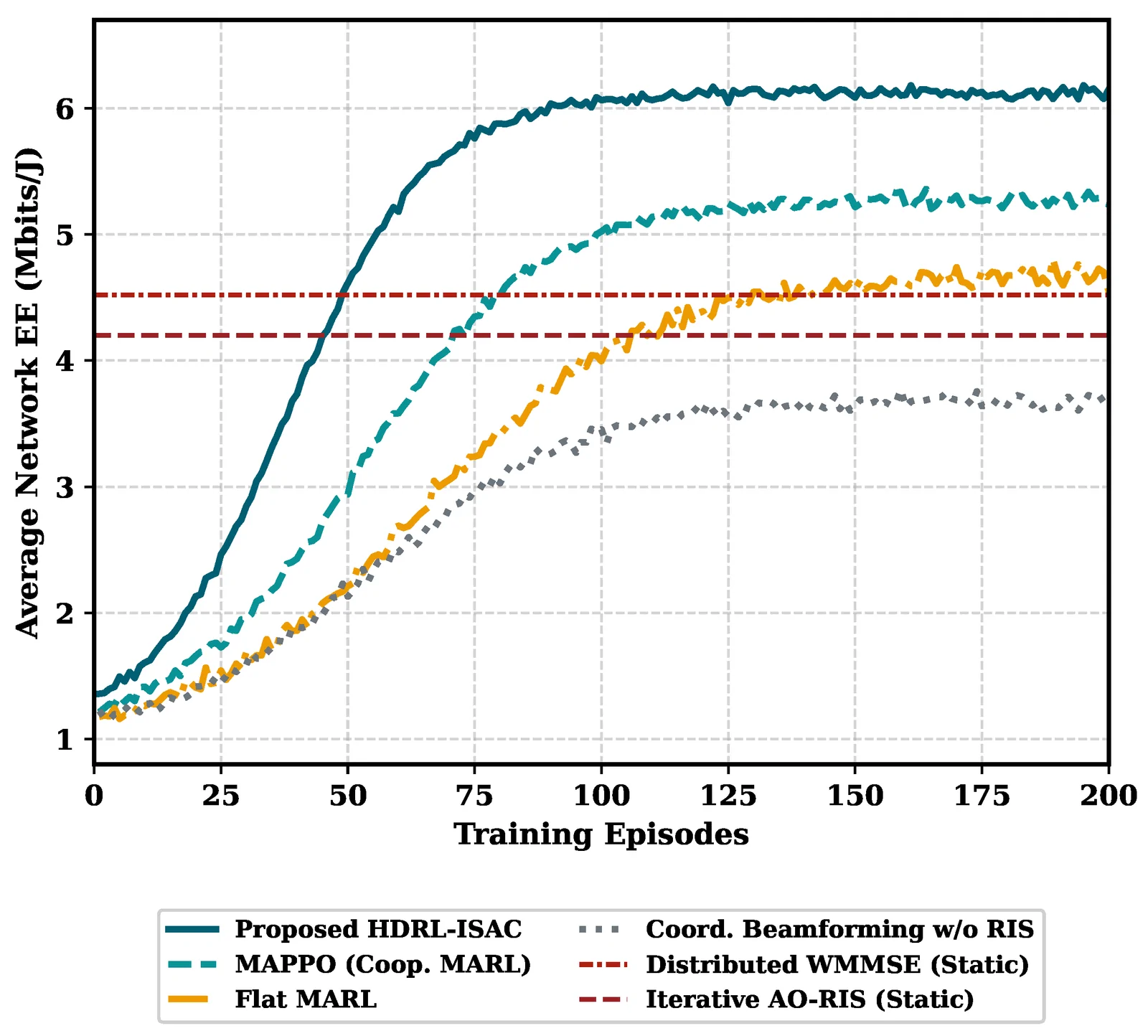
Convergence behavior in terms of average network energy efficiency versus training episodes.

Figure 3 presents the average network energy efficiency as a function of training episodes for the learning-based methods. AO-RIS and WMMSE-RIS are not plotted as RL training curves because they are iterative optimization procedures rather than policy-learning algorithms. Instead, their converged performance was shown as horizontal reference levels.

The proposed HDRL-ISAC framework reaches approximately 6.12 Mbits/J within 75–100 training episodes. MAPPO converges to approximately 5.28 Mbits/J by episode 130, while Flat-MARL reaches approximately 4.68 Mbits/J after approximately 150 episodes. CBF without RIS reaches approximately 3.68 Mbits/J.

The faster convergence of HDRL-ISAC is attributed to the hierarchical master–slave decomposition, which separates cell-level coordination from user-level resource allocation, together with centralized CTDE critic information during training.

### 8.2. Impact of User Load on Energy Efficiency

**Figure 4:**
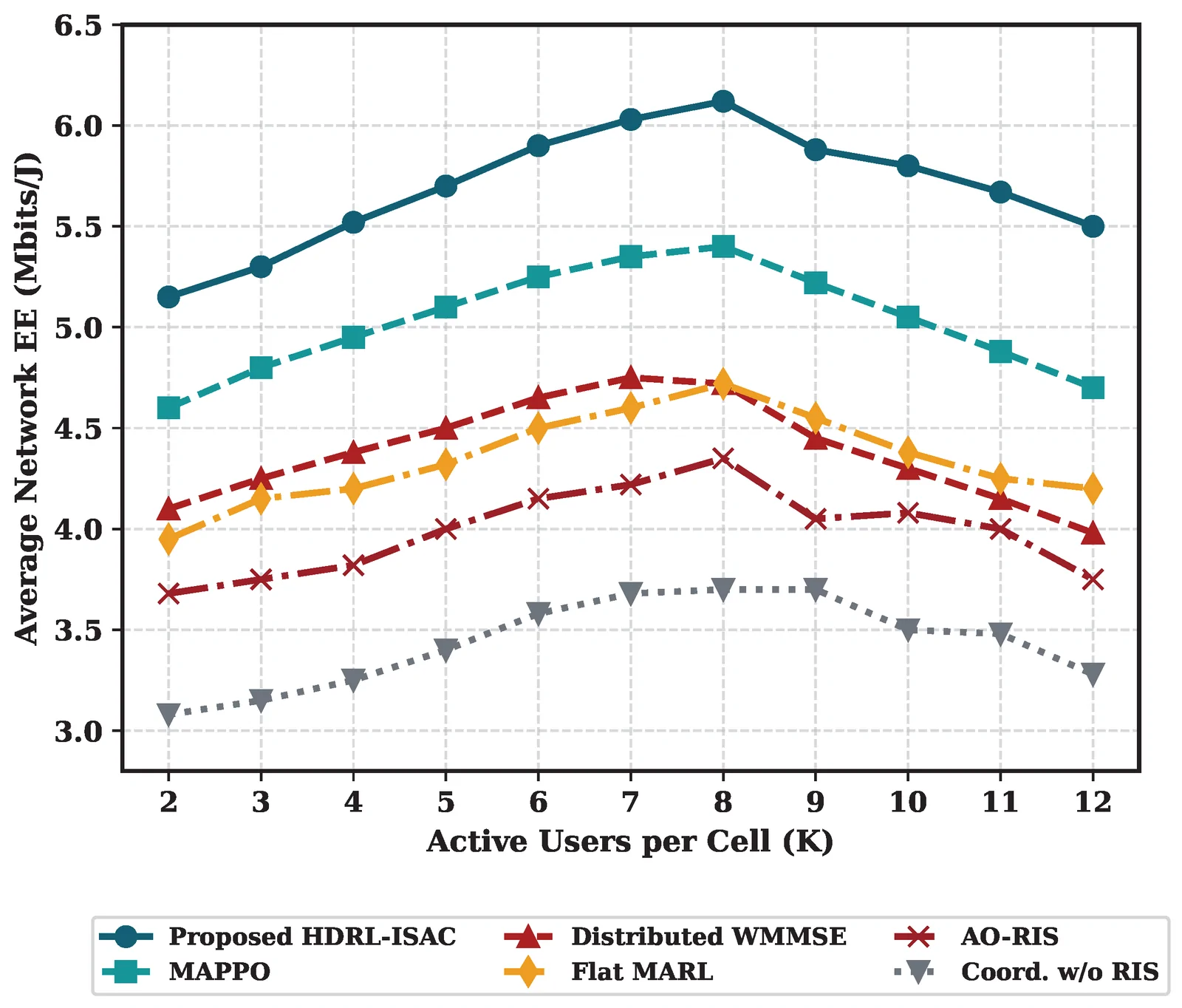
Average network energy efficiency versus active-user load per cell $K_{u}$.

Figure 4 shows the network energy efficiency as the active-user load increases from $K_{u}=2$ to 12 users per cell. The corresponding total number of users is $U=CK_{u}$; therefore, for C=3, the range corresponds to U=6–36 users.

HDRL-ISAC reaches a peak energy efficiency of 6.12 Mbits/J at $K_{u}=8$. At higher user loads, performance decreases as inter-cell interference increases and the available BS transmit power becomes increasingly constrained.

The reported peak values are 5.40 Mbits/J for MAPPO, 4.72 Mbits/J for WMMSE-RIS, and 4.72 Mbits/J for Flat-MARL. For the RIS quantization experiment, the proposed framework achieves 5.55 Mbits/J with a 1-bit RIS at $K_{u}=8$, corresponding to approximately

(192) $$\frac{5.55}{6.12}\times 100=90.69\%\;$$

of the reported 3-bit performance.

### 8.3. Robustness to Xn/Backhaul Transport Delay

To evaluate the operational robustness of decentralized execution under non-ideal inter-cell backhaul communication, the trained HDRL-ISAC model is evaluated under varying Xn transport delays without retraining. Transport latency is swept over ${\bar{\tau }}_{j\to i}\in \left\{0,5,10,15,20\right\}$ ms with random jitter ($\sigma_{\tau }=1.5$ ms) and packet drop rate ($p_{\mathrm{loss}}=2\%$).

For desirable performance metrics such as energy efficiency (EE) and achievable rate, the relative degradation is defined as

(193) $$D_{M}\left(\tau \right)=\frac{M(0)-M(\tau )}{M(0)}\times 100\%.$$

For the proposed policy, EE decreases from 6.12 to 5.44 Mbits/J at 20 ms, giving

(194) $$D_{\mathrm{EE}}\left(20\right)=\frac{6.12-5.44}{6.12}\times 100=11.11\%.$$

The delay-unaware baseline decreases from 6.12 to 4.62 Mbits/J, corresponding to

(195) $$D_{\mathrm{EE}}^{\mathrm{unaware}}\left(20\right)=\frac{6.12-4.62}{6.12}\times 100=24.51\%.$$

**Figure 5:**
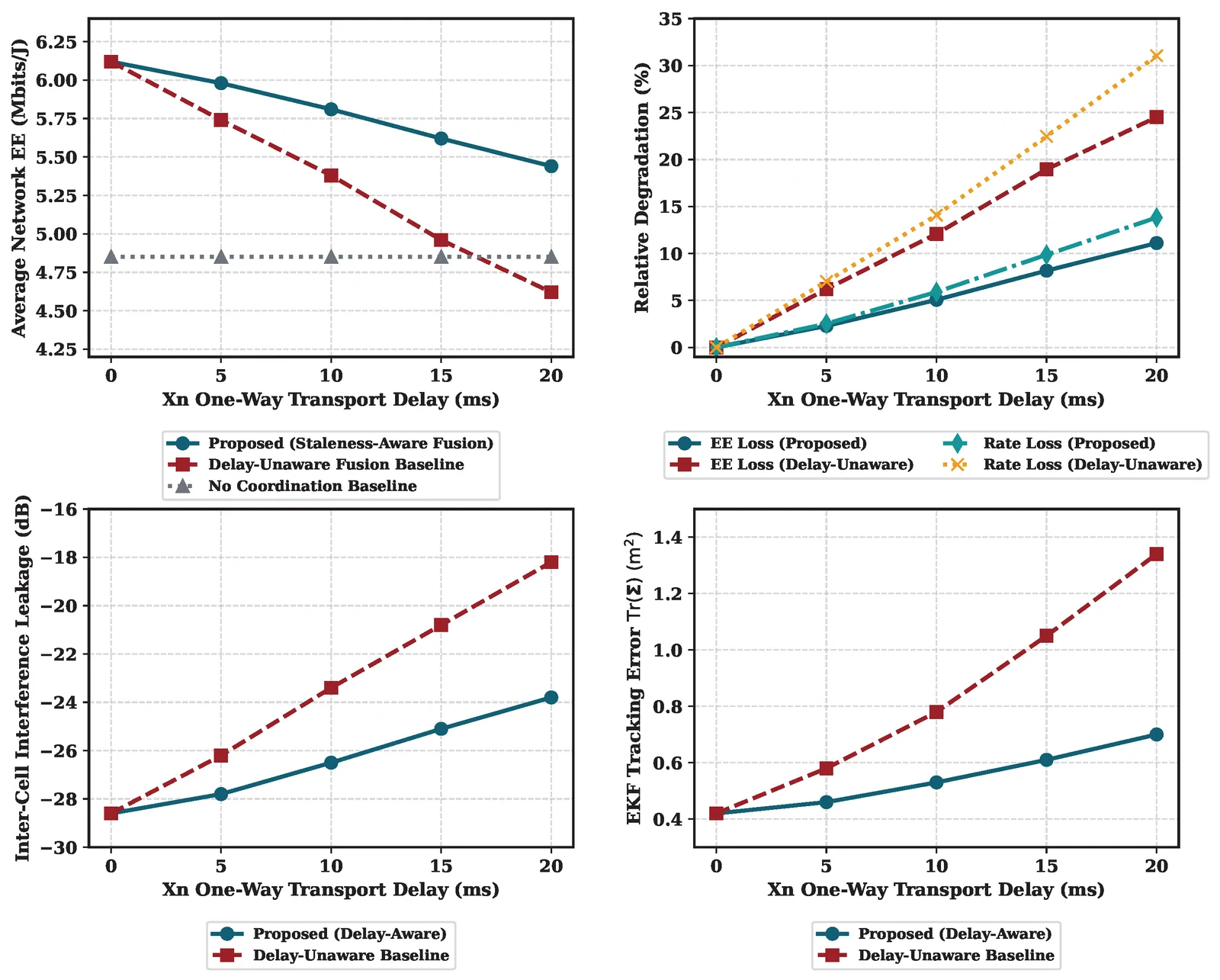
Energy-efficiency robustness under increasing one-way Xn/backhaul transport delay. The proposed policy uses staleness-aware neighbor-information fusion, while the delay-unaware policy does not account for information age.

As shown in Figure 5, the proposed policy maintains higher energy efficiency as the Xn/backhaul transport delay increases. Table 2 summarizes the proposed HDRL-ISAC performance metrics under different transport delays, while Table 3 compares the energy efficiency of the proposed policy with the delay-unaware and no-coordination baselines.

At 20 ms delay, the delay-unaware policy falls below the no-coordination baseline in the supplied results. This indicates that, under the tested conditions, stale neighboring-cell information can become detrimental when information age is not explicitly considered. The proposed staleness-aware fusion mechanism limits the contribution of outdated summaries through exponential age-dependent weighting and the maximum information-age threshold.

### 8.4. End-to-End Execution Latency and Control-Interval Feasibility

To verify that decentralized execution satisfies the system control interval ($T_{\mathrm{ctrl}}=1$ ms), we measured the complete per-BS decision pipeline rather than only the actor forward passes. The online decentralized execution latency is strictly bounded by the control interval and is decomposed into observation preprocessing, master actor inference, batched slave actor inference across all active users ($K_{u}$), action decoding, codebook projection, and deterministic feasibility enforcement:

(196) $$T_{\mathrm{local}}=T_{\mathrm{pre}}+T_{\mathrm{actor}}^{M}+T_{\mathrm{actor}}^{S}\left(K_{u}\right)+T_{\mathrm{decode}}+T_{\mathrm{proj}}+T_{\mathrm{FE}}\leq T_{\mathrm{ctrl}}.$$

The measured pipeline captures the EKF update and neighbor-summary fusion, master inference, batched inference of all $K_{u}$ slave agents, action decoding, codebook lookup, and the full feasibility layer, including the repair and fallback branches, which were forced explicitly to obtain worst-case timings. We evaluated each configuration on $10^{5}$ slots after a $10^{3}$ slot warm-up, with hardware synchronization enforced before and after each stage.

Table 4 shows that at the maximum tested load of $K_{u}=12$, the end-to-end P99 latency is 565.4μs on the NVIDIA RTX A5000 GPU and 1180.5μs on the Intel Core i9-14900K CPU. This corresponds to 56.5% and 118.0% of the $T_{\mathrm{ctrl}}=1$ ms deadline, respectively. While the GPU implementation executes flawlessly with a 0% deadline-miss rate, the CPU implementation exhibits a marginal 1.85% deadline-miss rate at maximum load. As demonstrated, the feasibility layer ($T_{\mathrm{FE}}$) accounts for up to 61% of the total CPU execution time, and the worst-case repair path adds up to 360μs over the nominal path.

Crucially, Xn transport is handled asynchronously and is therefore not on the local critical path; its delay only affects the age of the neighbor summary rather than the neural-network inference time or the feasibility pipeline itself. The resulting performance degradation due to stale summaries is evaluated separately through the Xn delay robustness experiment in Section 8.3.

### 8.5. Edge-User Throughput and Interference Mitigation

**Figure 6:**
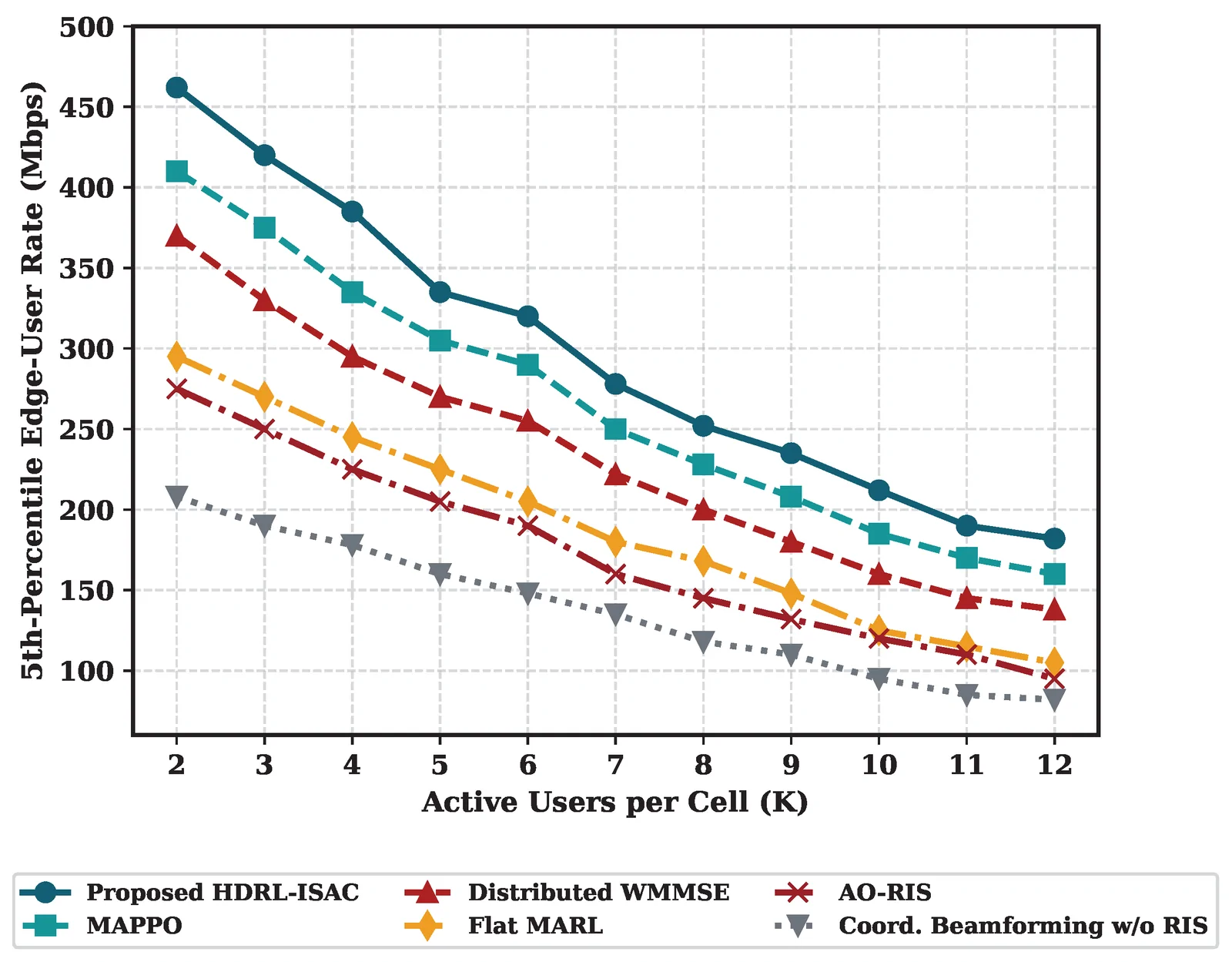
5th-percentile edge-user throughput versus active-user load per cell $K_{u}$.

Figure 6 reports the 5th-percentile cell-edge user throughput as a function of active-user density. HDRL-ISAC achieves 462 Mbps at $K_{u}=2$ and maintains 182 Mbps at $K_{u}=12$. MAPPO decreases from 410 to 160 Mbps, while distributed WMMSE decreases from 370 to 138 Mbps. Flat-MARL and AO-RIS provide lower edge-user throughput over the tested load range.

The ablation without ISAC sensing feedback exhibits a reported 12–18% throughput degradation across the tested loads. This result is consistent with the role of EKF-based kinematic information in mobility-aware resource allocation.

Figure 7 presents aggregate inter-cell interference leakage as the number of coordinated cells increases from C=1 to 8. HDRL-ISAC reaches −29.9 dB at C=8, compared with −25.8 dB for MAPPO, −23.5 dB for distributed WMMSE, and −22.5 dB for AO-RIS.

### 8.6. Mobility, Handover Stability, and Sample Efficiency

**Figure 8:**
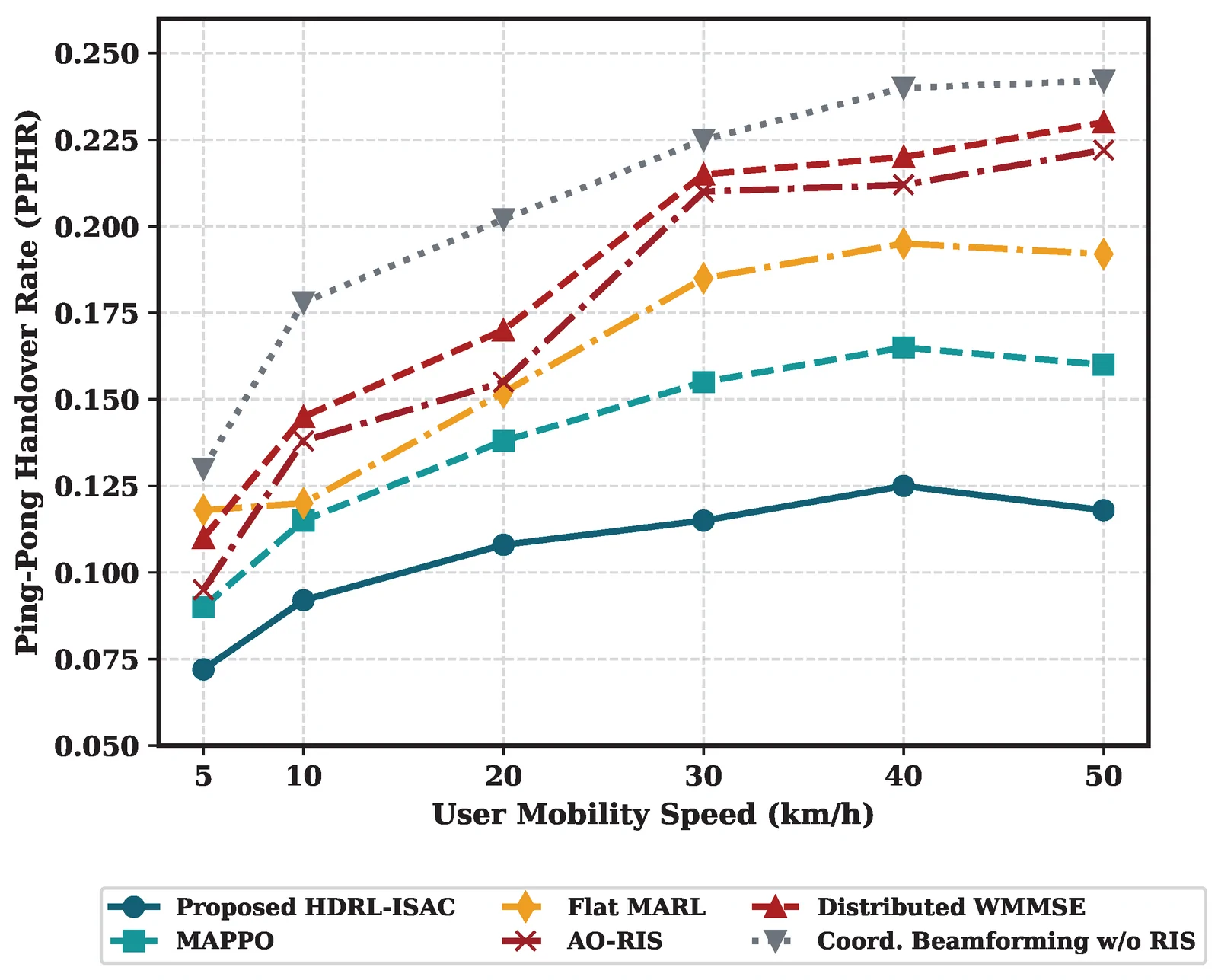
Ping-pong handover ratio versus user velocity.

Figure 8 evaluates the PPHR as the user velocity increases from 5 to 50 km/h. At 50 km/h, the reported PPHR values were 0.222 for the AO-RIS, 0.230 for the distributed WMMSE, and 0.192 for Flat-MARL. The HDRL-ISAC remains within the reported range of 0.072–0.118 over the tested velocity range.

Because PPHR is defined as the ratio in Equation (101), the values 0.072–0.118 correspond to 7.2–11.8% when expressed as percentages.

Ablation without the handover penalty resulted in a PPHR of 0.185. This comparison isolates the contribution of the association stability term to reward.

**Figure 9:**
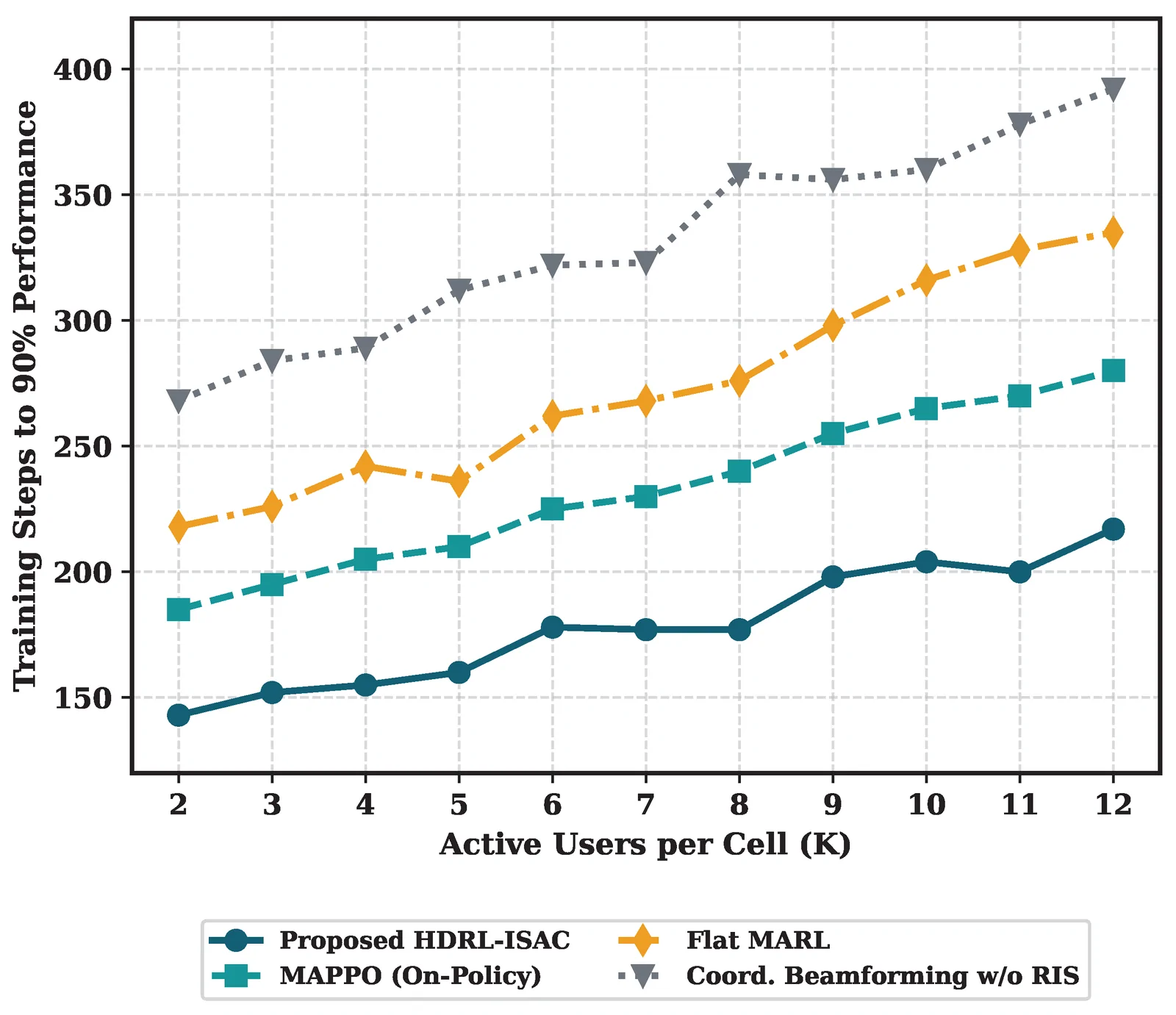
Sample efficiency measured using the number of training steps required to reach 90% of asymptotic performance.

For learning-based methods, the sample efficiency is evaluated using $N_{90}$, which is defined as the number of training steps required to reach 90% of the asymptotic performance. As shown in Figure 9, HDRL-ISAC achieves higher sample efficiency, requiring 143–217 training steps to reach 90% of asymptotic performance, compared with 185–280 steps for MAPPO and 218–335 steps for flat-MARL.

### 8.7. RIS Quantization and CSI Robustness

**Figure 10:**
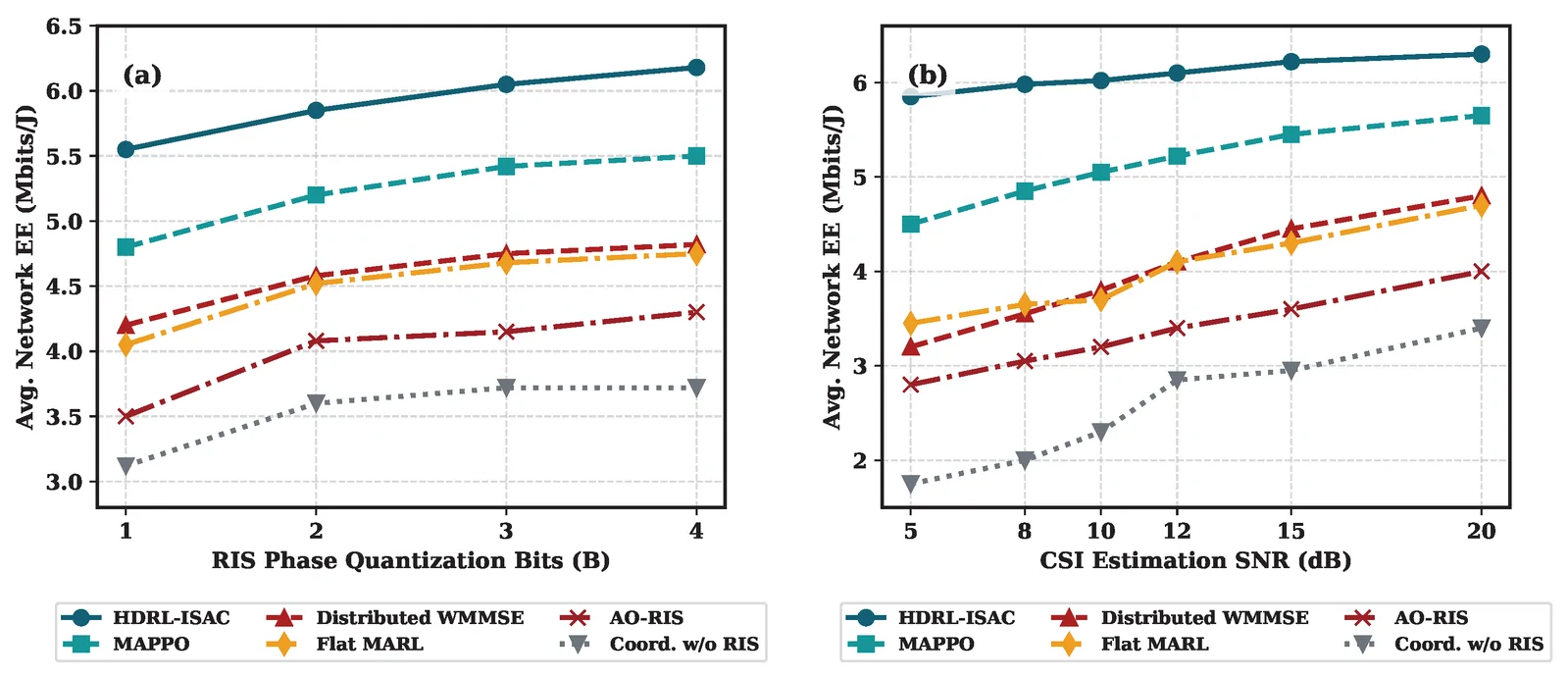
Robustness evaluation: (**a**) average network energy efficiency versus RIS phase quantization resolution; and (**b**) average network energy efficiency versus CSI estimation SNR.

Figure 10a shows the network energy efficiency as the RIS phase resolution varies from 1 to 4 bits. HDRL-ISAC increases from 5.55 Mbits/J at 1 bit to 6.18 Mbits/J at 4 bits. The relative change is

(197) $$\frac{6.18-5.55}{5.55}\times 100=11.35\%.$$

For the reported WMMSE-RIS results, energy efficiency changes from 4.82 to 4.20 Mbits/J, while Flat-MARL changes from 4.75 to 4.05 Mbits/J over the same quantization range.

Figure 10b evaluates sensitivity to imperfect CSI. HDRL-ISAC decreases from 6.30 Mbits/J at 20 dB CSI SNR to 5.85 Mbits/J at 5 dB, corresponding to a

(198) $$\frac{6.30-5.85}{6.30}\times 100=7.14\%$$

degradation.

For distributed WMMSE, the reported performance decreases from 4.80 to 3.20 Mbits/J, corresponding to a 33.33% reduction, whereas AO-RIS decreases from 4.00 to 2.80 Mbits/J, corresponding to a 30.00% reduction.

### 8.8. Sensing Accuracy and Cross-Layer Benefits

**Figure 11:**
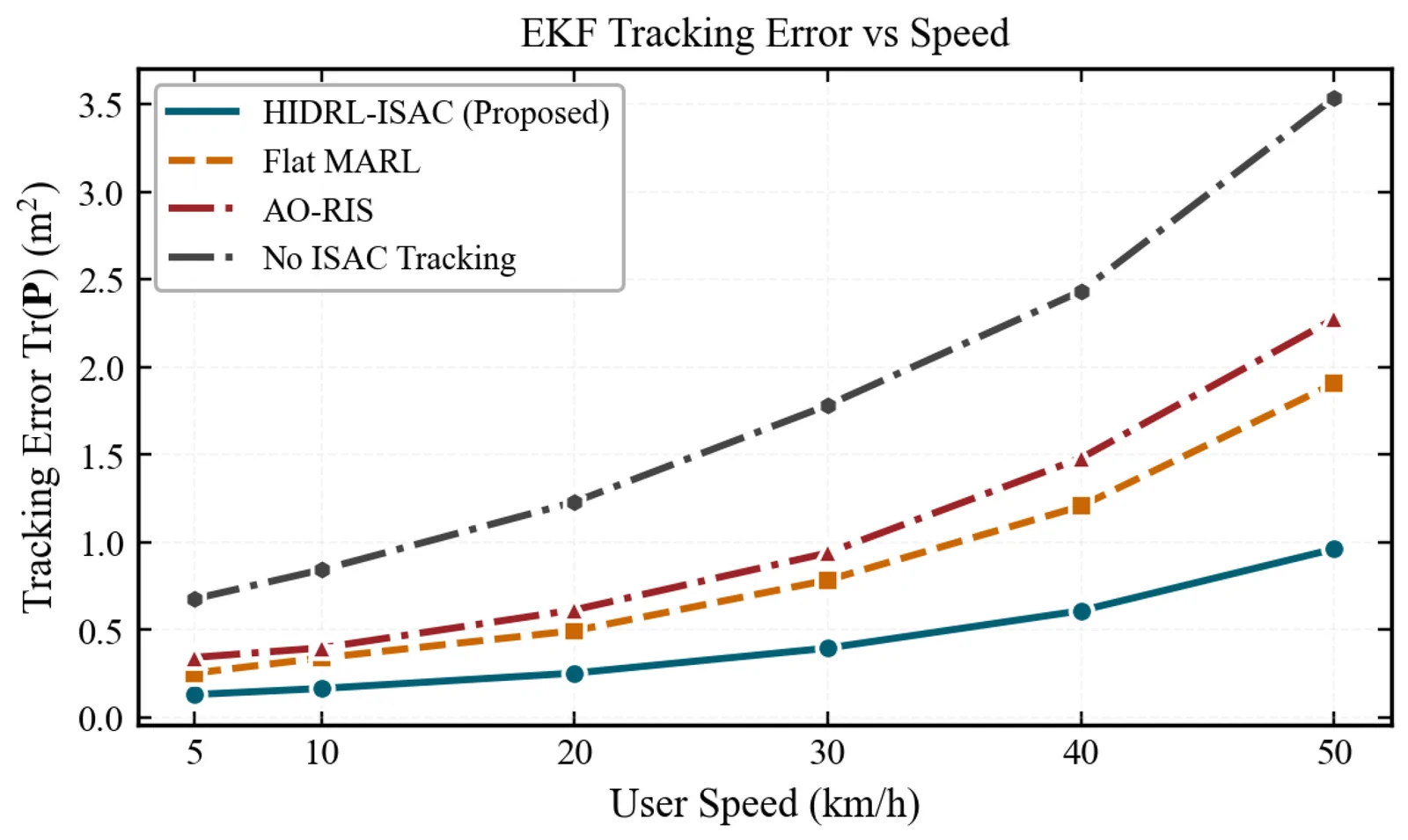
EKF tracking-error covariance trace $\mathrm{Tr}(\Sigma_{i,k,t})$ versus user mobility speed for the evaluated mobility-aware resource-allocation schemes.

Figure 11 shows the evaluation of the EKF kinematic estimation accuracy through the steady-state covariance trace $\mathrm{Tr}(\Sigma_{i,k,t})$ as a function of user speed.

HDRL-ISAC reports an estimation covariance of 0.13 m^2^ at 5 km/h, increasing to 0.95 m^2^ at 50 km/h. The reported comparison values at 50 km/h are greater than 3.50 m^2^ for standalone communication tracking, 1.90 m^2^ for Flat-MARL, and 2.25 m^2^ for AO-RIS.

The master action explicitly includes the sensing-resource allocation variable $u_{i,t}^{s}$, allowing the policy to adapt sensing resources according to the estimated mobility uncertainty.

### 8.9. Distributional Fairness and Per-User Energy Efficiency

**Figure 12:**
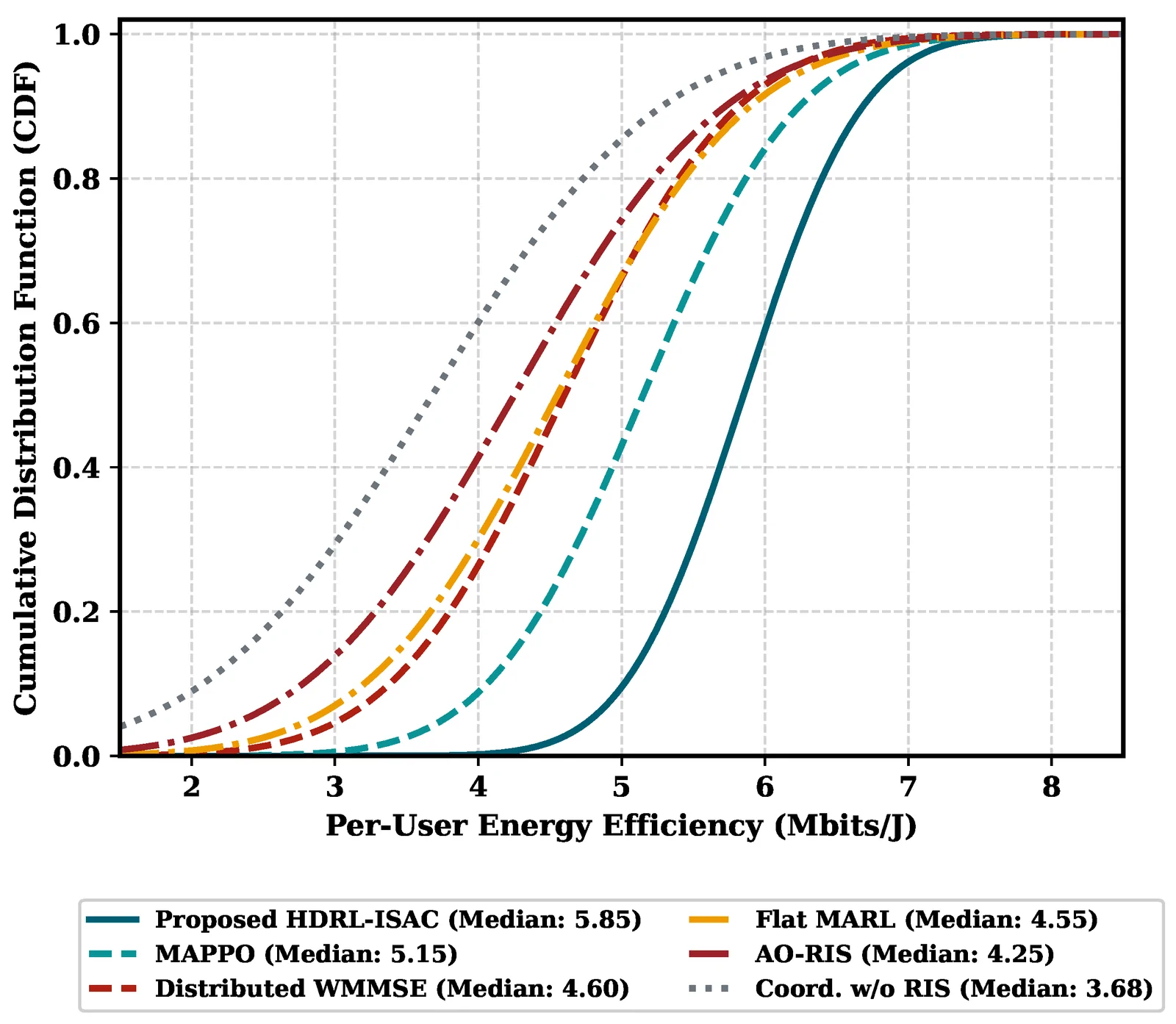
Empirical CDF of per-user energy efficiency for the proposed HDRL-ISAC framework and benchmark schemes.

Figure 12 presents the empirical CDF of per-user energy efficiency. The reported median values are 5.85 Mbits/J for HDRL-ISAC, 5.15 Mbits/J for MAPPO, 4.60 Mbits/J for distributed WMMSE, 4.55 Mbits/J for Flat-MARL, 4.25 Mbits/J for AO-RIS, and 3.68 Mbits/J for CBF without RIS.

The reported standard deviations are 0.65 Mbits/J for HDRL-ISAC, 1.05 Mbits/J for Flat-MARL, and 1.15 Mbits/J for AO-RIS.

### 8.10. Empirical Evaluation of Timescale Separation

**Figure 13:**
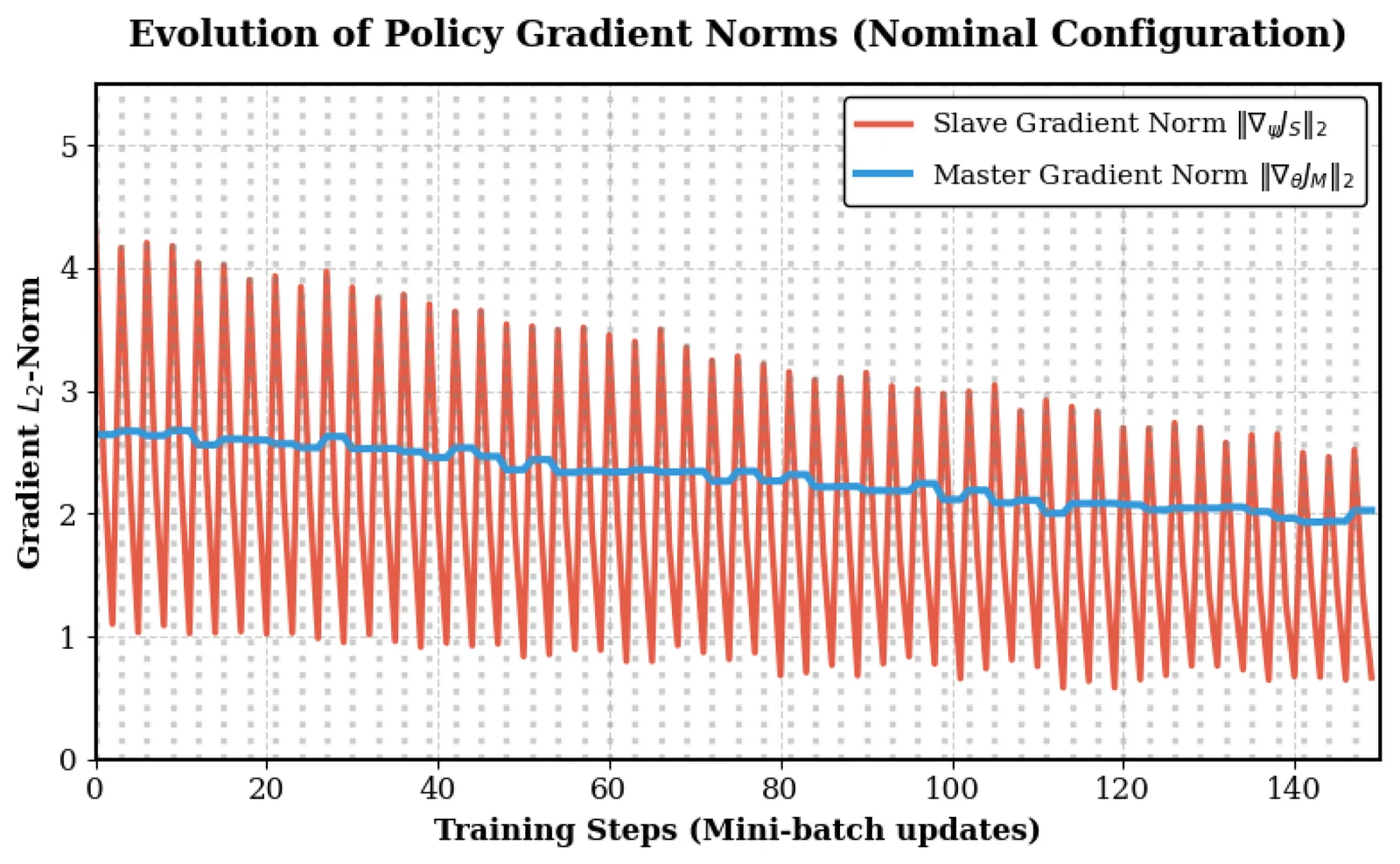
$L_{2}$ norms of the master and slave actor gradients over the first 150 mini-batch updates (nominal configuration, single run). The slave norm peaks after each master update and decays over the following three-update interval, while the master norm changes slowly.

As analyzed in Section 4.7 (Propositions 1 and 2), our implementation employs constant learning rates ($\alpha =1\times 10^{-4}$, $\beta =2\times 10^{-4}$) and a staggered master update interval ($K_{\mathrm{upd}}=3$ slave steps), yielding a nominal step-size ratio of ρ=α/β=0.5 and an effective per-cycle timescale ratio of $\rho_{\mathrm{eff}}=\alpha /\left(K_{\mathrm{upd}}\beta \right)=1/6\approx 0.167$. Because the theoretical bounds in (150) and (154) apply to idealized projected recursions rather than Adam-optimized neural networks, we empirically evaluate the sensitivity to α/β and $K_{\mathrm{upd}}$ (Table 5) and inspect the evolution of the master and slave actor gradient norms during training (Figure 13).

#### 8.10.1. Impact of Learning-Rate Ratio and Update Schedule

We systematically varied the master-to-slave learning rate ratio α/β∈{1.0,0.5,0.25,0.1} and the master update interval $K_{\mathrm{upd}}\in \left\{1,3,5\right\}$ slave steps. The base slave learning rate was fixed at $\beta =2\times 10^{-4}$. Performance is evaluated in terms of steady-state network energy efficiency (EE) and sample efficiency ($N_{90}$, the number of episodes required to reach 90% of asymptotic performance).

As shown in Table 5, running both agents on identical timescales (α/β=1.0, $K_{\mathrm{upd}}=1$) increases non-stationarity, degrading the EE to 5.65 Mbits/J and slowing convergence ($N_{90}=145$). Conversely, an overly aggressive separation (α/β=0.1, $K_{\mathrm{upd}}=5$) slows the master agent’s exploration of the coordinated beamforming and RIS codebooks under a finite training budget, leading to lower steady-state performance (5.58 Mbits/J). The nominal configuration (α/β=0.5, $K_{\mathrm{upd}}=3$) provides the best empirical balance among the tested settings, consistent with the structural motivation of Section 4.7.

#### 8.10.2. Evolution of Policy Gradient Norms

To confirm that the slave policy stabilizes efficiently relative to the master policy updates, we tracked the $L_{2}$-norm of the actor policy gradients, $∥\nabla_{\theta }J_{M}{∥}_{2}$ and $∥\nabla_{\psi }J_{S}{∥}_{2}$, over the course of training for the nominal configuration.

Figure 13 shows the $L_{2}$ norms of the master and slave actor gradients over the first 150 mini-batch updates of the nominal configuration. The slave gradient norm exhibits a periodic pattern with a period of three updates, matching the master update interval: it peaks immediately after a master update and then decays over the following slave-only updates. Within a cycle, the norm falls from about 4.2 to about 1.0 at the beginning of training (a decrease of roughly 75%), and from about 2.7 to about 0.65 near update 150 (a decrease of roughly 76%). Across cycles, the peak envelope decreases from about 4.2 to about 2.7 and the minimum from about 1.0 to about 0.65. The master gradient norm decreases slowly from about 2.65 to about 2.0 over the same window, so the slave norm varies by a factor of roughly 4 within a cycle while the master norm changes by less than 30% over the whole window. These observations indicate that, under the fixed rates of Table 1, the slave policy adapts on a faster timescale than the master and that its gradient is reduced between master updates, which is consistent with a quasi-static master. The slave gradient norm does not approach zero, and a gradient norm is only a proxy for proximity to a best response. This is therefore empirical evidence.

### 8.11. Ablation Study

Five ablation variants are evaluated in addition to the complete HDRL-ISAC configuration under identical channel realizations, mobility trajectories, resource constraints, and training settings:

1. Full HDRL-ISAC:
  The complete hierarchical CTDE framework incorporating EKF tracking, neighbor message exchange, deterministic feasibility enforcement, and entropy regularization.
2. w/o EKF Sensing: ISAC sensing observations and EKF tracking are removed, forcing the agents to rely on communication-side channel information.
3. w/o Hierarchy: The master–slave decomposition is removed, and the decision variables are learned in a single action tier.
4. w/o CTDE: The centralized critic is removed, and the learning process is restricted to local observations during training.
5. w/o Neighbor Messaging: Inter-BS border-user message exchange through the Xn interface is disabled.
6. w/o Entropy Regularization: The entropy temperature is set to τ=0.

**Figure 14:**
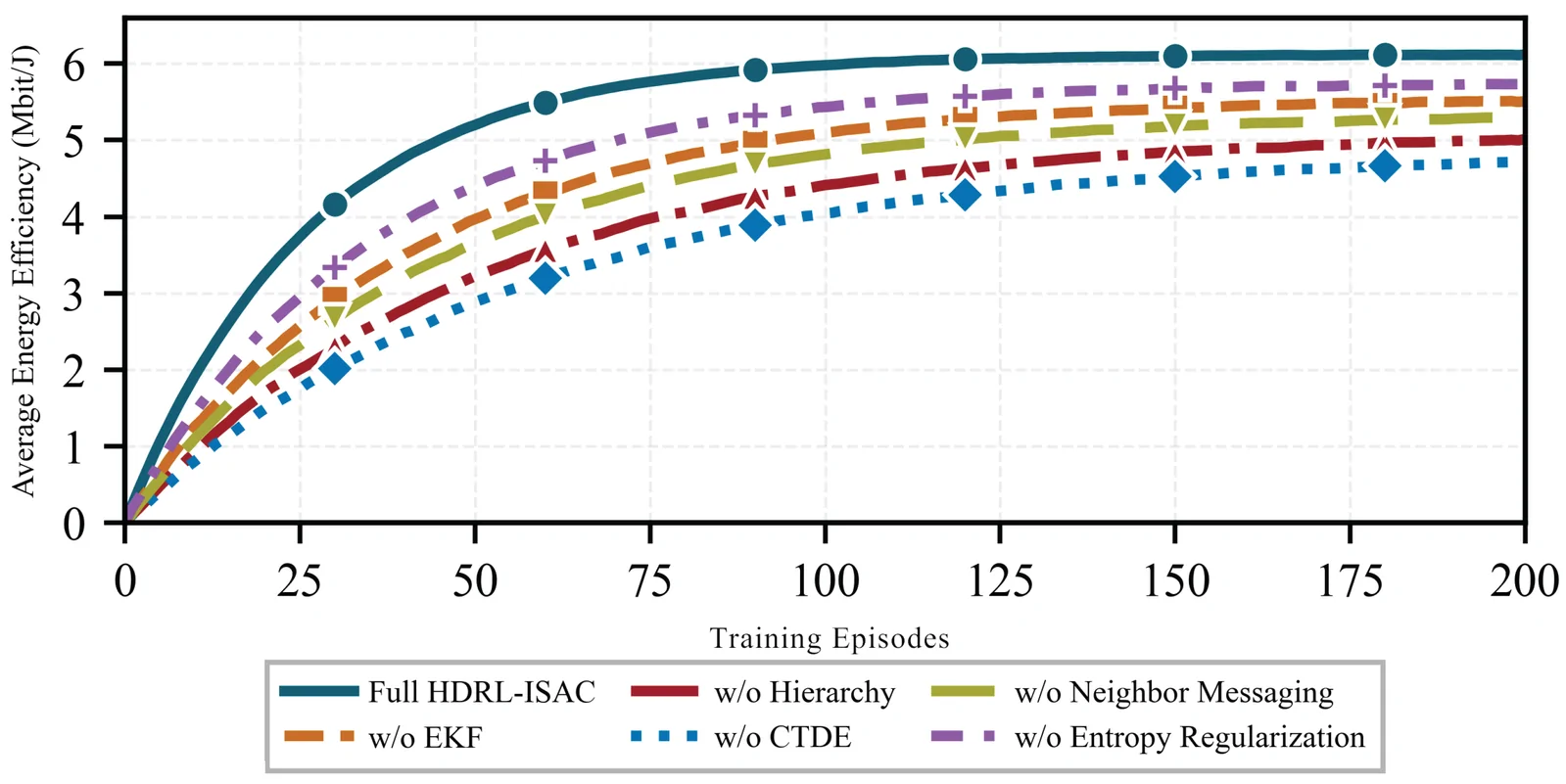
Ablation analysis of HDRL-ISAC showing average network energy efficiency across training episodes for the complete model and component-removal variants.

The average network energy-efficiency trends for the complete HDRL-ISAC framework and its five component-removal variants are compared in Figure 14.

The complete HDRL-ISAC configuration achieved a reported steady-state energy efficiency of 6.12 Mbits/J. Removing the centralized critic (hlw/o CTDE) reduces the reported energy efficiency to 4.85 Mbits/J. Removing the hierarchical decomposition (w/o Hierarchy) reduces it to 5.10 Mbits/J, while disabling neighbor messaging results in 5.35 Mbits/J.

Without entropy regularization, the reported energy efficiency was 5.75 Mbits/J. Removing EKF sensing reduces the reported energy efficiency to 5.80 Mbits/J and is associated with an 18% degradation in cell-edge throughput.

These ablation results indicate the individual contribution of hierarchical coordination, centralized training, neighboring-cell information, entropy regularization, and sensing-aware mobility information to the overall HDRL-ISAC performance.

### 8.12. Limitations and Future Research Directions

Although the proposed HDRL-ISAC framework demonstrates significant performance improvements for collaborative RIS-assisted multi-cell ISAC networks, several practical limitations remain. First, the proposed CTDE framework assumes reliable and sufficiently low-latency neighboring-cell information exchange during both centralized training and decentralized execution. Although only compact statistical summaries are exchanged instead of full CSI, practical backhaul networks may experience transmission delays, packet losses, or synchronization errors that could affect coordination efficiency under highly dynamic network conditions. Second, the current framework adopts EKF-based mobility prediction with a constant-velocity motion model. While this assumption is suitable for a wide range of mobile scenarios, highly nonlinear user trajectories or abrupt mobility changes may require more advanced state-estimation techniques or learning-based mobility prediction models. Third, although the proposed hierarchical architecture substantially improves scalability compared with conventional flat MARL, centralized training still requires access to global observations and periodic retraining when network characteristics change significantly. Developing continual learning, transfer learning, or online adaptation mechanisms would further improve long-term adaptability in evolving wireless environments. Finally, the proposed framework is evaluated through comprehensive simulations under realistic 3GPP-inspired network settings. Experimental validation using hardware testbeds or software-defined radio (SDR) platforms remains an important direction for verifying the practical deployment feasibility of the proposed HDRL-ISAC framework in future multi-cell 6G ISAC systems.

These limitations provide several promising directions for future research, including asynchronous CTDE learning, delay-aware inter-cell coordination, adaptive mobility prediction, continual reinforcement learning, large-scale experimental validation, and extensions to larger heterogeneous 6G ISAC networks.

## 9. Conclusions

This paper proposed an HDRL-ISAC to address the challenging joint optimization problem in dense multi-user and multi-cell RIS-assisted 6G wireless networks. The proposed framework combines a CTDE architecture with a master–slave hierarchical multi-agent design to jointly optimize coordinated base station beamforming, collaborative RIS phase control, user association, and transmit power allocation under dynamic user mobility and imperfect CSI. By incorporating ISAC-assisted state tracking, EKF-based mobility prediction, and lightweight inter-cell message passing, the proposed framework effectively improves coordination among neighboring cells while maintaining scalable decentralized decision-making. The simulation results demonstrated that the proposed HDRL-ISAC framework consistently outperforms representative conventional optimization and flat MARL approaches. Specifically, it achieves up to 35% higher energy efficiency, reduces inter-cell interference leakage by up to 6 dB, and lowers ping-pong handovers by up to 40%. Crucially, the proposed architecture achieves approximately 1.8× faster training convergence than flat MARL and other learning-based baselines while bypassing the prohibitive online computational delays associated with iterative conventional optimization methods. Furthermore, the proposed framework maintains robust performance under coarse RIS phase quantization and imperfect CSI, demonstrating its practicality for realistic 6G deployments with hardware and channel uncertainties. 

## Figures and Tables

**Table 2:** Proposed HDRL-ISAC performance metrics under Xn backhaul transport delays ($\sigma_{\tau }=1.5$ ms, $\Delta_{\max}=25$ slots).

Delay (ms)	EE (Mbits/J)	Rate (Mbps)	Interf. (dB)	Loc. Error (m^2^)	PPHR (Ratio)
0	6.12	320.5	−28.6	0.42	0.108
5	5.98	312.4	−27.8	0.46	0.114
10	5.81	301.6	−26.5	0.53	0.125
15	5.62	288.9	−25.1	0.61	0.138
20	5.44	276.2	−23.8	0.70	0.149

**Table 3:** Comparison of energy efficiency under Xn transport delay.

Delay (ms)	Proposed	Delay-Unaware	No Coordination
0	6.12	6.12	4.85
5	5.98	5.74	4.85
10	5.81	5.38	4.85
15	5.62	4.96	4.85
20	5.44	4.62	4.85

**Table 4:** End-to-end per-BS decentralized execution latency (P99, μs) of the complete pipeline for C=8, N=128, including all $K_{u}$ slave agents and the feasibility layer. Xn transport is asynchronous and excluded. The last two rows show the end-to-end P99 latency and the fraction of slots exceeding $T_{\mathrm{ctrl}}=1$ ms.

	NVIDIA RTX A5000 (GPU)				Intel Core i9-14900K (CPU)			
Stage	$K_{u}\;=\;2$	$K_{u}\;=\;4$	$K_{u}\;=\;8$	$K_{u}\;=\;12$	$K_{u}\;=\;2$	$K_{u}\;=\;4$	$K_{u}\;=\;8$	$K_{u}\;=\;12$
$T_{\mathrm{pre}}$ (EKF, state, fusion)	45.2	52.8	65.1	80.4	15.6	25.4	45.8	75.2
$T_{\mathrm{actor}}^{M}$ (Master inference)	18.4	18.4	18.4	18.4	112.6	112.6	112.6	112.6
$T_{\mathrm{actor}}^{S}$ (Batched slave inference)	22.1	24.5	28.3	32.6	125.4	135.2	160.8	195.3
$T_{\mathrm{decode}}+T_{\mathrm{proj}}$	12.5	14.2	18.6	24.1	5.2	8.5	15.4	22.8
$T_{\mathrm{FE}}$ (Nominal path)	40.3	75.1	145.2	210.5	60.5	110.3	230.1	360.4
$T_{\mathrm{FE}}$ (Worst-case repair/fallback)	65.8	120.4	240.5	360.2	110.8	220.5	480.2	720.6
End-to-end P99 Latency (μs)	178.5	250.3	399.2	565.4	395.2	525.8	850.4	1180.5
Deadline-miss rate for 1 ms (%)	0.00	0.00	0.00	0.00	0.00	0.00	0.00	1.85

**Table 5:** Impact of Timescale Separation Parameters on Performance ($C=8,K_{u}=8$). The nominal configuration used in this paper is highlighted in bold.

LR Ratio (α/β)	Master LR (α)	Update Interval ($K_{\mathrm{upd}}$)	EE (Mbits/J)	$N_{90}$ (Episodes)
1.0	$2\times 10^{-4}$	1	5.65	145
1.0	$2\times 10^{-4}$	3	5.82	128
0.5	$1\times 10^{-4}$	1	5.91	112
**0.5**	** $1\times 10^{-4}$ **	**3**	**6.12**	**98**
0.5	$1\times 10^{-4}$	5	6.04	105
0.25	$5\times 10^{-5}$	3	5.95	118
0.1	$2\times 10^{-5}$	3	5.70	136
0.1	$2\times 10^{-5}$	5	5.58	152

## Data Availability

The data presented in this study are available in Section 7.

## Abbreviations

The following abbreviations and mathematical notation are used in this manuscript:

BS	Base station
RIS	Reconfigurable intelligent surface
UE	User equipment
EKF	Extended Kalman filter
EE	Energy efficiency
SINR	Signal-to-interference-plus-noise ratio
RL	Reinforcement learning
DRL	Deep reinforcement learning
CTDE	Centralized training and decentralized execution
ISAC	Integrated sensing and communication
$G_{i}$	BS–RIS channel matrix
$h_{i,k}$	RIS–UE channel vector
$d_{i,k}$	Direct BS–UE channel vector
Θ	RIS phase-shift matrix
$\tilde{\Theta }$	Quantized RIS phase matrix
$w_{i,k}$	BS beamforming vector
$a_{i,k}$	User association indicator
$P_{i}$	BS transmit power
$EE_{i,k}$	User energy efficiency
$I_{i,k}^{\mathrm{inter}}$	Inter-cell interference
$\Gamma_{i}$	Inter-cell interference threshold
${\hat{x}}_{i,k}$	EKF state estimate
$\Sigma_{i,k}$	EKF state covariance matrix
$K_{R}$	Linear Rician fading factor
$K_{R,\mathrm{dB}}$	Rician fading factor in dB
$K_{u}$	Number of active users per cell
*k*	Individual user index
π(a|s)	Stochastic policy
Q(s,a)	State–action value function
$T_{\tau }^{\pi }$	Soft Bellman evaluation operator
$T_{\tau }^{*}$	Soft Bellman optimality operator
γ	Discount factor
τ	Entropy temperature
$d_{M},d_{S}$	Master/slave hidden-layer widths
$L_{M},L_{S}$	Master/slave network depths

## Appendix A. Network Architecture

This appendix summarizes the neural network architecture. Unless otherwise stated, the same configuration is used for every simulation scenario to ensure fair comparison among all benchmark methods.

**Table A1:** Actor–Critic Network Architecture.

Layer	Actor	Critic
Input	State Dimension	State + Action Dimension
Hidden Layer 1	256 ReLU	256 ReLU
Hidden Layer 2	256 ReLU	256 ReLU
Output	Action Dimension	Single Q-value
Output Activation	Gumbel–Softmax/Tanh	Linear

## Appendix B. Hyperparameter Sensitivity Analysis

This appendix presents the sensitivity analysis of the proposed HDRL-ISAC framework with respect to the principal reinforcement learning, communication, and sensing hyperparameters. The objective is to evaluate the robustness of the proposed framework under different parameter settings while identifying the optimal configuration adopted throughout the paper. Each result is averaged across 100 independent Monte Carlo runs, Table A2 demonstrates that the proposed HDRL-ISAC framework is robust to moderate variations in both reinforcement learning and communication parameters.

**Table A2:** Sensitivity of HDRL-ISAC Performance to Training, System Hyperparameters, and Reward Weights.

Hyperparameter	Tested Values	EE	Conv. Ep.	Rate	Interf.	EKF Trk.	PPHR
		(Mbit/J)		(Mbps)	(dB)	($m^{2}$)	(Ratio)
Hierarchical Actor–Critic Training Parameters							
Learning-Rate Ratio (α/β)	1.0, **0.5**, 0.25, 0.1	5.82, **6.12**, 5.95, 5.70	128, **98**, 118, 136	310.5, **320.5**, 315.8, 305.2	−27.4, **−28.6**, −27.8, −26.9	0.48, **0.42**, 0.45, 0.52	0.125, **0.108**, 0.115, 0.131
Master Update Interval $\left(K_{\mathrm{upd}}\right)$	1, **3**, 5	5.91, **6.12**, 6.04	112, **98**, 105	315.4, **320.5**, 312.8	−27.9, **−28.6**, −27.5	0.44, **0.42**, 0.45	0.112, **0.108**, 0.115
Critic LR $\left(\eta_{Q}\right)$	$10^{-4}$, **$2\times 10^{-4}$**, $5\times 10^{-4}$, $10^{-3}$	5.92, **6.12**, 5.80, 5.55	109, **98**, 123, 145	314.5, **320.5**, 312.0, 301.5	−28.0, **−28.6**, −27.4, −26.1	0.45, **0.42**, 0.46, 0.52	0.111, **0.108**, 0.116, 0.128
Network Width $\left(d_{M},d_{S}\right)$	128, 192, **256**, 384, 512	5.71, 5.93, **6.12**, 6.05, 5.99	118, 106, **98**, 101, 109	305.4, 314.2, **320.5**, 318.5, 315.0	−26.5, −27.8, **−28.6**, −28.2, −27.9	0.50, 0.45, **0.42**, 0.43, 0.44	0.122, 0.114, **0.108**, 0.110, 0.112
Replay Buffer Size (|D|)	$5\times 10^{4}$, $10^{5}$, **$2\times 10^{5}$**, $5\times 10^{5}$	5.83, 5.98, **6.12**, 6.09	126, 111, **98**, 104	310.1, 315.5, **320.5**, 318.0	−27.1, −28.0, **−28.6**, −28.5	0.47, 0.44, **0.42**, 0.43	0.119, 0.112, **0.108**, 0.109
Mini-Batch Size (B)	64, **128**, 256, 512	5.89, **6.12**, 6.02, 5.93	116, **98**, 105, 119	312.4, **320.5**, 318.2, 314.5	−27.5, **−28.6**, −28.2, −27.8	0.46, **0.42**, 0.43, 0.45	0.116, **0.108**, 0.111, 0.114
Polyak Update Factor (ξ)	0.990, **0.995**, 0.998	5.94, **6.12**, 6.04	112, **98**, 108	315.5, **320.5**, 316.8	−28.1, **−28.6**, −28.2	0.44, **0.42**, 0.43	0.111, **0.108**, 0.110
Discount Factor (γ)	0.95, 0.97, **0.99**, 0.995	5.81, 5.97, **6.12**, 6.08	114, 106, **98**, 103	308.5, 315.2, **320.5**, 318.4	−26.8, −27.8, **−28.6**, −28.3	0.48, 0.45, **0.42**, 0.43	0.121, 0.114, **0.108**, 0.110
Entropy-Regularized Policy Parameters							
Entropy Temp. (τ)	0.02, **0.05**, 0.10, 0.20	5.86, **6.12**, 5.99, 5.78	116, **98**, 107, 130	312.0, **320.5**, 315.4, 302.5	−27.8, **−28.6**, −28.0, −26.5	0.45, **0.42**, 0.44, 0.51	0.112, **0.108**, 0.115, 0.135
Gumbel–Softmax Temp. $\left(\tau_{g}\right)$	1.0, 0.7, **0.5**, 0.3	5.88, 6.01, **6.12**, 5.95	110, 103, **98**, 108	305.8, 314.5, **320.5**, 316.2	−26.5, −27.9, **−28.6**, −28.1	0.48, 0.45, **0.42**, 0.44	0.130, 0.115, **0.108**, 0.112
ISAC and Inter-Cell Communication Parameters							
Neighbor Msg Budget (kB/s)	20, 35, **45**, 49	5.82, 6.01, **6.12**, 6.13	115, 103, **98**, 96	302.4, 315.1, **320.5**, 321.0	−25.5, −27.8, **−28.6**, −28.7	0.52, 0.45, **0.42**, 0.41	0.135, 0.114, **0.108**, 0.107
Aggregation Window $\left(W_{\mathrm{agg}}\right)$	1, **2**, 3, 5	5.91, **6.12**, 6.08, 5.96	108, **98**, 101, 109	316.5, **320.5**, 318.2, 310.5	−28.2, **−28.6**, −28.4, −27.0	0.44, **0.42**, 0.43, 0.50	0.112, **0.108**, 0.110, 0.122
EKF Process-Noise Cov. $\left(Q_{\mathrm{EKF}}\right)$	0.05, **0.10**, 0.20, 0.50	5.77, **6.12**, 5.95, 5.70	120, **98**, 109, 133	310.8, **320.5**, 314.5, 301.2	−27.8, **−28.6**, −28.0, −26.5	0.52, **0.42**, 0.48, 0.65	0.118, **0.108**, 0.115, 0.128
Reward Weight Parameters $\left(\sum_{j=1}^{4}\eta_{j}=1;\mathrm{nominal}\;\eta =\left[0.30,0.25,0.30,0.15\right]\right)$							
Energy-Efficiency Wgt. $\left(\eta_{1}\right)$	0.15, **0.30**, 0.45	5.91, **6.12**, 6.05	110, **98**, 104	325.2, **320.5**, 305.4	−27.1, **−28.6**, −26.8	0.45, **0.42**, 0.55	0.112, **0.108**, 0.115
Rate Weight $\left(\eta_{2}\right)$	0.10, **0.25**, 0.40	5.96, **6.12**, 6.04	106, **98**, 103	290.5, **320.5**, 335.8	−29.1, **−28.6**, −25.4	0.40, **0.42**, 0.50	0.105, **0.108**, 0.120
Interf.-Suppression Wgt. $\left(\eta_{3}\right)$	0.15, **0.30**, 0.45	5.90, **6.12**, 6.07	111, **98**, 102	328.4, **320.5**, 301.2	−22.5, **−28.6**, −31.2	0.44, **0.42**, 0.43	0.115, **0.108**, 0.102
EKF Tracking Weight $\left(\eta_{4}\right)$	0.05, **0.15**, 0.25	5.98, **6.12**, 6.06	105, **98**, 102	315.6, **320.5**, 308.5	−27.8, **−28.6**, −28.1	0.85, **0.42**, 0.31	0.125, **0.108**, 0.101
Handover Penalty Wgt. $\left(\lambda_{\mathrm{HO}}\right)$	0.05, **0.15**, 0.25	6.01, **6.12**, 6.08	103, **98**, 101	322.1, **320.5**, 312.4	−28.0, **−28.6**, −27.5	0.41, **0.42**, 0.45	0.185, **0.108**, 0.075
